# The Discovery of a New Mimivirus Isolate in Association with Virophage-Transpoviron Elements in Brazil Highlights the Main Genomic and Evolutionary Features of This Tripartite System

**DOI:** 10.3390/v14020206

**Published:** 2022-01-21

**Authors:** Bruna Luiza de Azevedo, João Pessoa Araújo Júnior, Leila Sabrina Ullmann, Rodrigo Araújo Lima Rodrigues, Jônatas Santos Abrahão

**Affiliations:** 1 Laboratório de Vírus, Departamento de Microbiologia, Universidade Federal de Minas Gerais (UFMG), Belo Horizonte 31270-901, MG, Brazil; azvdobruna@gmail.com; 2 Laboratório de Virologia, Departamento de Microbiologia e Imunologia, Instituto de Biotecnologia, Universidade Estadual Paulista (Unesp), Alameda das Tecomarias s/n, Chácara Capão Bonito, Botucatu 18607-440, SP, Brazil; jpessoa@ibb.unesp.br (J.P.A.J.); leila_ullmann@yahoo.com.br (L.S.U.)

**Keywords:** giant viruses, mimivirus, virophage, transpoviron

## Abstract

Mimiviruses are giant viruses of amoeba that can be found in association with virophages. These satellite-like viruses are dependent on the mimivirus viral factory to replicate. Mimiviruses can also be associated with linear DNA molecules called transpovirons. Transpovirons and virophages are important drivers of giant virus evolution although they are still poorly studied elements. Here, we describe the isolation and genomic characterization of a mimivirus/virophage/transpoviron tripartite system from Brazil. We analyzed transmission electron microscopy images and performed genome sequencing and assembly, gene annotation, and phylogenetic analysis. Our data confirm the isolation of a lineage A mimivirus (1.2 Mb/1012 ORFs), called mimivirus argentum, and a sputnik virophage (18,880 bp/20 ORFs). We also detected a third sequence corresponding to a transpoviron from clade A (6365 bp/6 ORFs) that presents small terminal inverted repeats (77 nt). The main genomic features of mimivirus argentum and of its virophage/transpoviron elements corroborates with what is described for other known elements. This highlights that this triple genomic and biological interaction may be ancient and well-conserved. The results expand the basic knowledge about unique and little-known elements and pave the way to future studies that might contribute to a better understanding of this tripartite relationship.

## 1. Introduction

Viruses are known as abundant, diverse, and ubiquitous entities. They are usually defined considering criteria such as their small particles and genomes and their dependence on the host-cell machinery to synthesize energy and proteins [1]. However, some of these concepts started to be questioned in 2003 with the discovery of the amoeba-infecting virus named Acanthamoeba polyphaga mimivirus (APMV) [2]. Mimiviruses caught the attention of virologists because of their large particles (~750 nm) and genomes (~1.2 megabases) as well as their capacity to code several genes involved in functions never described in the virosphere before (e.g., protein translation) [2,3,4]. In addition, the most part of their genes have completely unknown functions and no matches with any other sequences in databases (ORFans) [2,3,4].

APMV was the first member of a new family of viruses named *Mimiviridae*. In recent years, new types of amoeba-infecting mimiviruses have been described, forming different phylogenetic lineages, such as the so-called lineages A, B, and C of mimiviruses [5,6,7]; the tupanviruses [8,9]; and the klosneuviruses [10]. Currently, *Mimiviridae* is classified into a phylum called *Nucleocytoviricota* [11]. This taxon is composed of different families of large DNA viruses, such as *Poxviridae* and *Phycodnaviridae*, and of other groups of giant viruses of amoeba, such as *Marseilleviridae*, pandoraviruses, and pithoviruses [12,13,14,15]. Besides the description of new giant virus groups, the first isolation of mimiviruses opened the doors to a world of intriguing discoveries.

One of these discoveries was the isolation of sputnik, the first described virophage. In 2008, during the isolation of Acanthamoeba castellanii mamavirus (a mimivirus isolate), several smaller icosahedral particles (~50 nm) were observed to be associated with the viral factory (VF) of the giant virus [16]. It was claimed that sputnik can affect mamavirus progeny productivity by hijacking important virus-encoded elements, such as transcription-related proteins [17]. Furthermore, virophages can integrate their genomes into the giant virus or host cell genomes, forming provirophages [18,19]. These virophages now compose their own viral family, the *Lavidaviridae* [20], which has been expanded recently. Besides the sputnik-like isolates, this family clusters other lineages of some isolated virophages (e.g., mavirus and zamilon) [21,22] and several virophages detected through metagenomic or genomic studies [23,24].

Virophages can be considered important players in giant virus evolution. A different type of mobile genetic element, called transpovirons, can also be involved in this scenario [25]. Together with provirophages, transpovirons are proposed to be part of the unique mimiviruses mobilome [18]. Mobilome is classically defined as the set of all mobile genetic elements (MGEs) encoded by a cell, and the presence of MGEs in viral genomes is usually proposed to be rare [26,27]. Transpovirons are linear DNA sequences (~7 kilobase pairs) found in association with different mimivirus lineages (A, B, and C). It is suggested that they are dependent on the mimivirus VF and proteins to replicate [18]. The relationship between transpovirons, virophages, giant viruses, and eukaryotic host cells seems to be a rich and complex biological interaction involving the virosphere. This can be exemplified by a study showing that virophages act as transpoviron propagation vehicles between giant viruses, forming a complex tripartite system [28]. To our knowledge, six transpovirons sequences were described in previous works. However, only two of them were described in a tripartite system with both mimivirus and virophage after isolation assays [18,28]. Thus, research describing or characterizing this type of triple interaction is still scarce. In this work, we report the detection of a mimivirus-virophage-transpoviron tripartite system for the first time in Brazil and describe the morphological, genomic, and phylogenetic characteristics of these three elements.

## 2. Materials and Methods

### 2.1. Viral Isolation, Multiplication, and Purification

The isolates were obtained from water samples collected in 2015 at Pampulha Lagoon (Belo Horizonte, Minas Gerais, Brazil), using a prospecting protocol that consists in a direct inoculation of the collected samples on amoeba cultures [29]. To control the assay, we used wells for a non-inoculated cell control. After the isolation, the virus was inoculated at a multiplicity of infection (MOI) of 0.01 in cell culture roller bottles containing 1.4 × 10^7^ *Acanthamoeba castellanii* cells and 35 mL of peptone-yeast extract-glucose (PYG) medium supplemented with penicillin (100 U/mL; Cellofarm, Brazil), streptomycin (100 µg/mL; Sigma-Aldrich, Burlington, MA, USA), and amphotericin B (0.25 µg/mL; Cultilab, Brazil). The cells were incubated at 32 °C under slow rotation (0.2 rpm) on a roller. After the observation of cytopathic effects caused by viral infection (i.e., rounding cells and cellular lysis), the flask’s content was collected. This content was subjected to freezing and thawing for three times, aiming to lyse the cells that remained intact. Then, it was ultracentrifuged (36,000× *g*) in a 22% sucrose cushion for 30 min. The pellet containing purified viral particles was resuspended in phosphate-buffered-saline (PBS 1×), and the viral titers were obtained through the end-point method [30]. Note: the viruses and sequences obtained or accessed during this work are registered under the SISGEN (national system for the management of genetic heritage and associated traditional knowledge) numbers A473BD3/A702EB8/ABF23CC/A580BBD and SISBIO (national system for authorization and information on biodiversity) number 34293.

### 2.2. Transmission Electron Microscopy

To analyze the morphology of the isolated viral particles, we prepared the samples for observation through transmission electron microscopy (TEM). Thus, 7 × 10^6^ *A. castellanii* cells cultured in 25 mL of PYG medium were inoculated with the virus at an MOI of 0.01. After the observation of cytopathic effect (rounding cells), the cells were washed twice with 0.1 M sodium phosphate buffer and fixed with a solution containing 0.1 M sodium phosphate buffer and 2.5% glutaraldehyde for 2 h under rotation at room temperature. The cells were then fixed with osmium tetroxide (2%) and embedded in Epon resin, which allowed ultramicrotomy (60-nm thick) and observation using a transmission electron microscope (Spirit Biotwin FEI-120 kV) at the Center of Microscopy of Federal University of Minas Gerais (CM-UFMG). During one TEM analysis, we observed at least 20 different cells, in different sections, all containing mimiviruses and virophages particles.

### 2.3. Sequencing, Assembly, and Annotation

The samples containing the purified virus were sequenced using an Illumina MiSeq instrument with a paired-end library using the kit Illumina DNA Prep (Illumina Inc., San Diego, CA, USA). The FastQC program was used to quality control of the obtained reads, which were trimmed using the Trimmomatic tool [31]. For genome de-novo assembly, we used Spades 3.12 with default parameters [32,33]. The assembled scaffolds corresponding to the giant virus were ordered based on a reference genome using MeDuSa online [34]. The reference genomes used were the APMV or the mamavirus sequences, obtained at NCBI database (GenBank Accession Numbers: HQ336222.2 and JQ063128.1, respectively). Open reading frames (ORFs) were predicted with the GeneMarkS tool [35], considering only proteins that were bigger than 50 amino acids. Additionally, tRNA coding sequences were predicted using ARAGORN [36]. The predicted ORFs were annotated using BLASTp (expect threshold: 10-3) against the NCBI non-redundant protein sequences (nr) database. Eventually, we also used the HHpred server [37] to predict functions and/or structures for ORFs (databases: PDB_mmCIF70, PDB_mmCIF30 and SCOPe70_2.07), considering as valid hits those that presented probabilities greater than 95% or e-values equal to or smaller than 1. The search for terminal inverted repeats (TIRs) was performed using the Inverted Repeat Finder program with default parameters [38]. Representative genome maps were elaborated using CG view server [39] and the SnapGene (Insightful Science) and Geneious Prime 2021.0.3 softwares.

### 2.4. Phylogeny Analysis

Maximum-likelihood phylogenetic trees were constructed through IQtree software (version 1.6.12) using 1000 bootstrap replicates as branch support [40]. The datasets containing the sequences used for alignments were prepared using BLASTp (expected threshold: 10^−3^) against NCBI non-redundant protein sequences (nr) database. To align the sequences, we used the MUSCLE algorithm executed through the MEGA X program [41,42]. The best-fit substitution models were selected by the ModelFinder algorithm implemented in IQtree [43]. Finally, the phylogenetic trees were visualized and edited using MEGA X software and iTOL [42,44].

### 2.5. DNA Extraction and PCR

The sample containing purified viruses was submitted to DNA extraction and polymerase chain reaction (PCR) to verify the integration of a virophage into the giant virus genome. For this, 200 µL of the sample was separated for DNA extraction performed with the High Pure Viral Nucleic Acid Kit (Roche) according to manufacturer’s instructions. Primers were designed through a manual search in the sequence of interest, and their quality was evaluated using the OlygoAnalyzer tool. Primer sequences are targeted to both ends of the putative virophage integration site and to the initial region of ORF12, encoded by the virophage (Table 1). For each PCR reaction, 3 µL of extracted DNA (~50 ng/µL) was added into a mix containing 3 µL of buffer (10 µM), 0.9 µL of MgCl2 (50 µM), 0.6 µL of dNTP mix (10 µM), 0.3 µL of Taq DNA polymerase, and 1.5 µL of forward and reverse primers (10 µM). The final volume of the reaction was adjusted to 30 µL using ultrapure water. PCR assays were conducted in a MasterCycler thermal cycler (Eppendorf, Germany) with the following conditions: 95 °C for 10 min, followed by 30 cycles of 1 min at 95 °C, 1 min at 45 °C, and 1 min at 72 °C, followed by a final step of 10 min at 72 °C and 4 °C until storage. The results were read after electrophoresis (120 V) on a 2% agarose gel (Sigma-Aldrich, Burlington, MA, USA). DNA extracted from purified APMV particles was used as the negative control.

## 3. Results and Discussion

### 3.1. Isolation of a Tripartite Mimivirus-Virophage-Transpoviron System in Brazil

**Figure 1:**
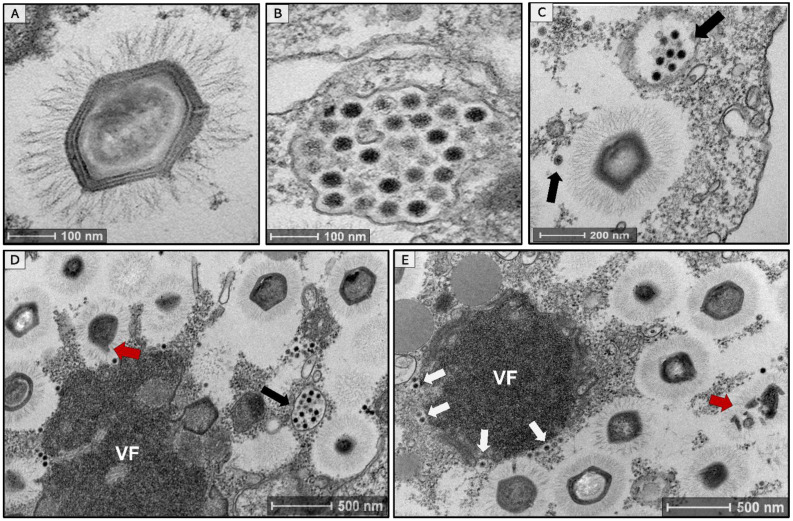
An overview of mimivirus argentum and its associated virophage (sputnik argentum) inside *Acanthamoeba castellanii* cells. Transmission electron microscopy images showing (**A**) a mimivirus-like particle (~673 nm) with a capsid surrounded by fibrils; (**B**) a vesicle containing virophage-like icosahedral particles (~47 nm); (**C**) virophage-like particles (black arrows) appear grouped near a mature mimivirus particle; and (**D**) the mimivirus viral factory (VF) surrounded by mature mimivirus particles. It is possible to observe virophage-like particles inside a vesicle (black arrow); (**E**) forming virophage-like particles in one of the poles of mimivirus VF (white arrows). The red arrows indicate defective mimivirus particles.

During the prospecting assays, the *Acanthamoeba castellanii* cells presented some cytopathic effects (e.g., rounding and lysis of cells) typically caused by the presence of giant viruses in the sample. We were able to conclude this because we used a non-inoculated cellular control whose cells remained healthy and did not present the mentioned cytopathic effects during the assay. Thus, our first step was to prepare a sample for TEM observation aiming to identify this virus based on its morphological characteristics. As soon as we observed the images, we saw mimivirus-like particles on average 673 nm in size (*n* = 50 particles), which was named mimivirus argentum (Figure 1A). Alongside the mimivirus particles, we also observed smaller icosahedral particles, on average 47 nm in size (*n* = 60 particles), suggesting the presence of virophages in the sample (Figure 1B). These smaller particles were observed in several cells, organized in groups, surrounding mature mimiviruses particles or within vesicles (Figure 1C,D, black arrows). When analyzing the viral factory (VF), we observed mimiviruses particle morphogenesis (Figure 1D); in a late stage of the cycle, it was also possible to observe the virophage particles being formed in one of the poles of the mimivirus VF (Figure 1E, white arrows). This was already described before for sputnik-like virophages [45]. In addition, the formation of defective mimivirus particles can be seen in different cells (Figure 1D,E, red arrows). The presence of these defective particles was previously associated with a decrease in mimivirus productivity caused by the presence of virophages during the replication cycle [16]. However, it has been described that defective particles can also be formed independently of virophage presence [46]. Thus, it is not possible to affirm that the formation of defective mimivirus particles is necessarily caused by the presence of virophage in the cell and more studies are needed to clarify this point.

The second step after virus isolation was genome sequencing. A total of 359,614 reads, ranging from 35 to 251 base pairs (bp), were generated and de-novo assembled into 10 scaffolds. By analyzing these scaffolds with BLASTn, we observed that eight of them matched with the Acanthamoeba castellanii mamavirus genome. A 18,880-bp scaffold, with a high coverage number (693×) and a circular topology, matched with sputnik virophage 2. This confirmed the presence of a virophage in the sample, as observed in TEM images. The remaining scaffold had 6365 bp (312× coverage) and matched with the mamavirus-associated transpoviron. Then, all the scaffolds were organized based on the APMV genome (GenBank Accession Number: HQ336222.2). Three scaffolds were generated, with one representing the mimivirus argentum complete genome and the other two corresponding to complete sequences of a virophage and a transpoviron, referred to here as sputnik argentum and mimivirus argentum transpoviron, respectively. Thus, we describe for the first time, to our knowledge, a tripartite system involving a mimivirus, a virophage, and a transpoviron, obtained in Brazil.

### 3.2. Mimivirus Argentum Genome

**Figure 2:**
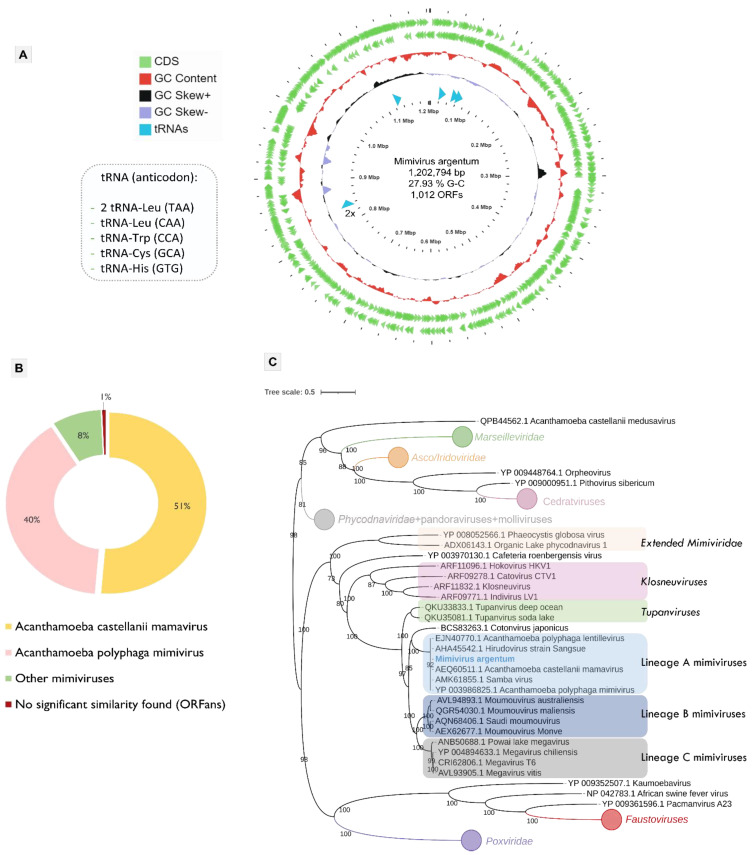
Genomic and phylogenetic features of mimivirus argentum. (**A**) Representative map of the mimivirus argentum genome. In the distribution of ORFs and tRNAs throughout the sequence, the G-C content and G-C skew are represented by different ring colors, as indicated in the color legend. The outer green ring represents the positive sense strand, whereas the inner green ring represents the negative sense strand. The box in the left specifies the six tRNAs coded by mimivirus argentum. The tRNA blue arrow indicated with “2×” shows two tRNAs closely located in genome. (**B**) Characterization of BLASTp best hits obtained during mimivirus argentum genome annotation. (**C**) Maximum-likelihood phylogenetic tree of nucleocytoviricota based on the DNA polymerase B amino acids sequences (ORF678 coded by mimivirus argentum). The results observed in this tree can be reinforced by other phylogenetic trees based on nucleocytoviricota conserved proteins, which are shown in the supplementary material (Figure S1A–C). The mimivirus argentum sequence is labeled with blue and bold font. The best-fit model chosen with ModelFinder (implemented in IQtree) for this tree was VT + F + R5. The tree scale bar represents the number of amino acid substitutions per site. CDS, coding sequences; tRNA, transfer RNA.

The final assembled scaffold of mimivirus argentum genome is composed of 1,202,794 bp with a G-C content of 27.93%. The gene prediction revealed 1012 ORFs distributed in both DNA strands, with coding proteins with sizes that range from 51 to 2959 amino acid residues (Figure 2A). The search for similar sequences showed that the most part of mimivirus argentum proteins matched with mamavirus (51%) and APMV (40%) (Figure 2B). Regarding the protein functions, 56% of them are unknown or hypothetical proteins. In addition, seven coding sequences had no significant similarity found in the database analyzed, which expands the set of mimivirus ORFans (Figure 2B).

Mimivirus argentum encodes several genes involved in different metabolic functions, including the protein translation process (Table S1). These translation-related genes include five types of translation factors, including initiation factors (IF4A, IF4E, and SUI1), an elongation factor eF-Tu, and two chain release factors (erf1). In addition, four types of aminoacyl-tRNA-synthetases (aaRS) were found: arginyl, methlonyl, cysteinyl, and tyrosyl tRNA synthetases. Moreover, we identified six transfer RNAs (tRNAs) genes, indicated in Figure 2A. We did not observe any translation-associated genes that was not described before for other mimiviruses. Therefore, the mimivirus argentum genome shows considerable similarity with other lineage A mimiviruses (3, 4, 28). This fact was reinforced by the phylogenetic analyses based on some nucleocytoviricota conserved proteins, such as the DNA polymerase family B (Figure 2C, Figure S1A), the VV A32 virion packaging ATPase (Figure S1B), and the major capsid protein (Figure S1C). All the phylogenetic trees constructed include mimivirus argentum into the lineage A of mimiviruses within the *Mimiviridae* family. This is interesting because previous works suggested an abundance of mimiviruses, especially from lineage A, in Brazilian samples [29,47]. It is hypothesized that this abundance may be related either to a high affinity of these viruses for *Acanthamoeba* cells or to assays with specific types of samples, such as water from urban lakes, both commonly used in prospecting studies in Brazil [29,47].

### 3.3. Sputnik Argentum Genome

**Figure 3:**
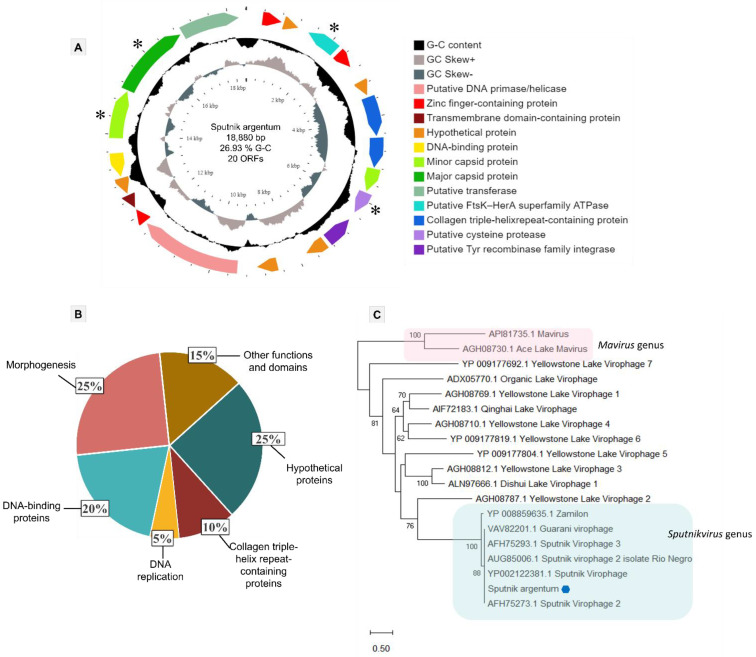
Genome characterization and phylogeny of sputnik argentum. (**A**) Genome representative map of sputnik argentum, showing the G-C content, G-C skew, and the distribution of 20 annotated ORFs throughout the sequence. Predicted function and/or domains are indicated by different arrow colors as described in the color legend. Right-directed arrows represent positive strand ORFs, and left-directed arrows represent negative strand ORFs. Asterisks indicate the core proteins of the Lavidaviridae family. (**B**) Pie chart highlighting the proportion of different protein functions and/or domains and repeats in the sputnik argentum genome. (**C**) *Lavidaviridae* family maximum-likelihood tree based on the major capsid protein (MCP) amino acid sequence encoded by sputnik argentum (ORF19). The sputnik argentum sequence is indicated by a blue hexagon. The best fit model chosen with ModelFinder (implemented in IQtree) for this tree was rtREV + F + I + G4. The tree scale bar represents the number of amino acid substitutions per site.

The sputnik argentum virophage genome is composed of a circular double-strand DNA molecule, with 18,880 bp and a G-C content of 26.93%. It encodes 20 proteins ranging in size between 109 and 779 amino acids residues (Figure 3A). All proteins had BLASTp best hits matching with either sputnik 1 or sputnik 2 virophages, with high amino acid identity (Table S2). The final annotation, including a HHpred analysis, allowed us to identify the functions and/or domains of 15 of the 20 predicted proteins (Figure 3A). This analysis indicates that 25% (5/20) of the sputnik argentum genome encodes for unknown or hypothetical proteins (ORFs 2, 5, 11, 12, and 16). The remaining 75% of the sputnik argentum proteome is predicted to include 20% DNA-binding domain-containing proteins (4/20), 25% morphogenesis-related proteins (5/20), 10% collagen triple-helix repeat-containing proteins (2/20), 5% representing a DNA replication-protein (1/20), and 15% proteins with other functions and/or domains (3/20) (Figure 3B).

The DNA-binding domain-containing proteins category includes zinc finger-containing proteins (ORFs 1,3, and 14) and a helix-turn-helix domain-containing protein (ORF17) that is homologous to a prokaryotic transcriptional regulator. Regarding DNA replication, a putative primase/helicase (ORF13) was found, which was predicted to be associated with this function in virophages [48]. The proteins considered in the category of other functions and/or domains are a putative Tyr recombinase family integrase (ORF10), a transmembrane domain-containing protein (ORF15), and a putative transferase (ORF20), which is homologous to mimiviruses. Differently from our annotation, the homologs of ORF 20 (putative transferase), coded by other previously described virophages, are annotated in databases as a hypothetical protein (i.e., unknown function). The morphogenesis-related protein category is composed of a set of *Lavidaviridae* conserved proteins that includes the superfamily FtsK-HerA packaging ATPase (ORF3), a cysteine protease (ORF9), a major capsid protein (MCP) (ORF19), and two minor virion proteins (mCP) (ORFs 8 and 18). This set of core proteins is typically used for virophages phylogenetic analysis. The phylogenetic tree based on MCP reinforced the clustering of sputnik argentum together with other sputnik-like virophages that compose the *Sputnikvirus* genus (Figure 3C). The same result was observed when analyzing the phylogeny of the other conserved genes (Figure S2).

Some years ago, the first mimivirus (lentillevirus) associated with a provirophage and a transpoviron was described [18]. Based on that, we tested our samples containing mimivirus argentum and its virophage with a protocol known to inactivate virophages. This protocol involves treatment with ethanol, heat, and desiccation [18]. The sample with the putatively inactivated virophage was inoculated into *A. castellanii* cells. Even so, the virophage continued its multiplication, which was confirmed through sequencing. After sequencing and de-novo genome assembly, we organized the obtained scaffolds based on a reference genome as described in Section 3.1 Firstly, we used the mamavirus genome (GenBank Accession Number: JQ063128.1) as a reference since it was the best hit in the BLASTn analysis. This mamavirus-guided assembly generated two scaffolds (mimivirus+virophage and transpoviron). The virophage genome was found in the middle of the mimivirus argentum sequence in a region flanked by collagen-like proteins (between 957,704 and 976,583 nucleotides) (Figure S3A). These collagen-like proteins were already described as putative hot spots for virophage integration [18]. These results suggested that the virophage sequence was fully integrated into the mimivirus argentum genome. However, unlike mamavirus-guided assembly, when we used the APMV genome as a reference, we obtained three scaffolds (mimivirus, virophage, and transpoviron). This is intriguing because the putative virophage integration (two scaffolds assembly) appears only when we use a specific genome as reference (mamavirus). To compare our results, we tried the same scaffold organization protocol with external data: the 10 contigs of lentillevirus available in GenBank (Accession Number: AFYC00000000.1), the sputnik 2 available genome (Accession Number: JN603369.1), and the lentillevirus-associated transpoviron (Accession Number: JQ063127.1) In this analysis, the virophage sequence also appeared integrated into the lentillevirus genome only when the mamavirus genome was used as reference; the same result was obtained with our sequences.

To verify virophage integration biologically, primers were designed to target both flanking regions of the putative integration site in the mimivirus argentum genome (Table 1, Figure S3B). We also designed primers for a region in the middle of the sputnik argentum genome in ORF12 (Table 1, Figure S3B). Surprisingly, after the PCR assays, we only observed amplification of the virophage genome (ORF12) and did not observe any amplification of the putative integration region (Figure S3C). This unexpected result led us to discard the hypothesis of virophage integration in the mimivirus argentum genome. This result suggested that the inactivation protocol usually used to inactivate virophages might not be efficient for all virophages. The resistance of virophages to heat inactivation has been already described [28]. In addition, the insertion of the virophage sequence into the mimivirus argentum genome observed after the reference-guided assembly may have happened due to a methodological bias. We hypothesize that the region containing collagen-like proteins, which shows sequence similarity with virophage genes, guided the insertion during the reference-based assembly. On the other hand, when the reference genome used to assemble is the APMV sequence, the integration is not observed. Similar results were observed when analyzing the lentillevirus genome. This is intriguing because APMV also encodes genes for collagen-like proteins. Therefore, deeper investigations are needed to better understand this question.

### 3.4. Mimivirus Argentum-Associated Transpoviron

**Figure 4:**
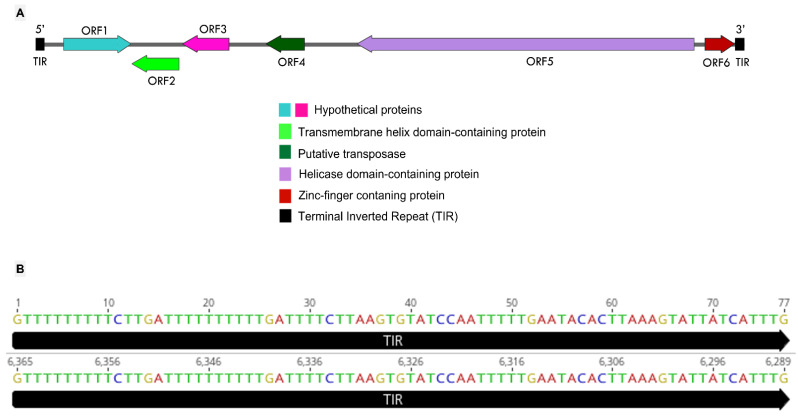
The mimivirus argentum transpoviron genome. (**A**) Mimivirus argentum-associated transpoviron genome map showing the six predicted ORFs and the respective annotation, coded by different arrow colors, as shown in the legend above. Right-directed arrows represent positive strand ORFs, and left-directed arrows represent negative strand ORFs. Terminal inverted repeats (TIRs) are represented by black squares. (**B**) Representation of mimivirus argentum transpoviron TIRs by black arrows, showing the inverted match (100%) of its nucleotides and its position in the genome. The 3′ TIR (6289–6365 nt) is represented by its reverse complement.

The third component of the tripartite system described here is the transpoviron detected in association but apart from the mimivirus and sputnik argentum genomes. Its sequence is composed of 6365 bp, with 25.03% G-C content. A total of six ORFs were predicted to encode proteins ranging between 89 and 999 amino acid residues. The BLASTp analysis revealed the best hits with transpovirons associated with lentillevirus and mamavirus (Table S3). The final annotation of the predicted proteins, including HHpred analysis, allowed us to identify the functions and/or domains of four of the six proteins. These four proteins were composed of a transmembrane helix domain-containing protein (ORF2), a putative transposase (ORF4), a helicase domain-containing protein (ORF5), and a zinc-finger containing protein (ORF6). The two remaining proteins (ORFs 1 and 3) were annotated as hypothetical proteins since their characteristics are unknown (Figure 4A).

As well as the other described transpovirons, the mimivirus argentum transpoviron also presents terminal inverted repeats (TIRs) (Figure 4A). Each of these repeats is composed of 77 nucleotides, which match 100% with each other (Figure 4B). These are much smaller TIRs in comparison with the other known transpovirons, which are described to have TIRs bigger than 300 nucleotides [28]. This contradicts the previously proposed idea that transpoviron TIRs are well-conserved within clades [28]. In typical DNA transposons, one of the roles of TIRs is to be recognized by the transposase, an enzyme responsible for the “cut and paste” transposition mechanism [49]. By using this mechanism, DNA transposons can move from a genome region that has been already replicated to a region that has not, thus increasing the number of copies [49]. Considering that transpovirons from clade A and B encode a transposase homolog, it is possible that their TIRs may also be involved in the replication process that would happen by taking advantage of mimivirus DNA replication. The presence of these smaller TIRs in the mimivirus argentum transpoviron might be explained by the smaller size of its whole sequence. The mamavirus and lentillevirus transpovirons have at least 700 more nucleotides than mimivirus argentum transpoviron although these three sequences present high sequence similarity. The mimivirus argentum transpoviron is also smaller than the other known transpoviron sequences except for partial deposited sequences (moumouvirus monve and megavirus courdo7 transpovirons). Therefore, our results highlight the need for deeper investigations that explore the mechanisms behind transpovirons biology.

**Figure 5:**
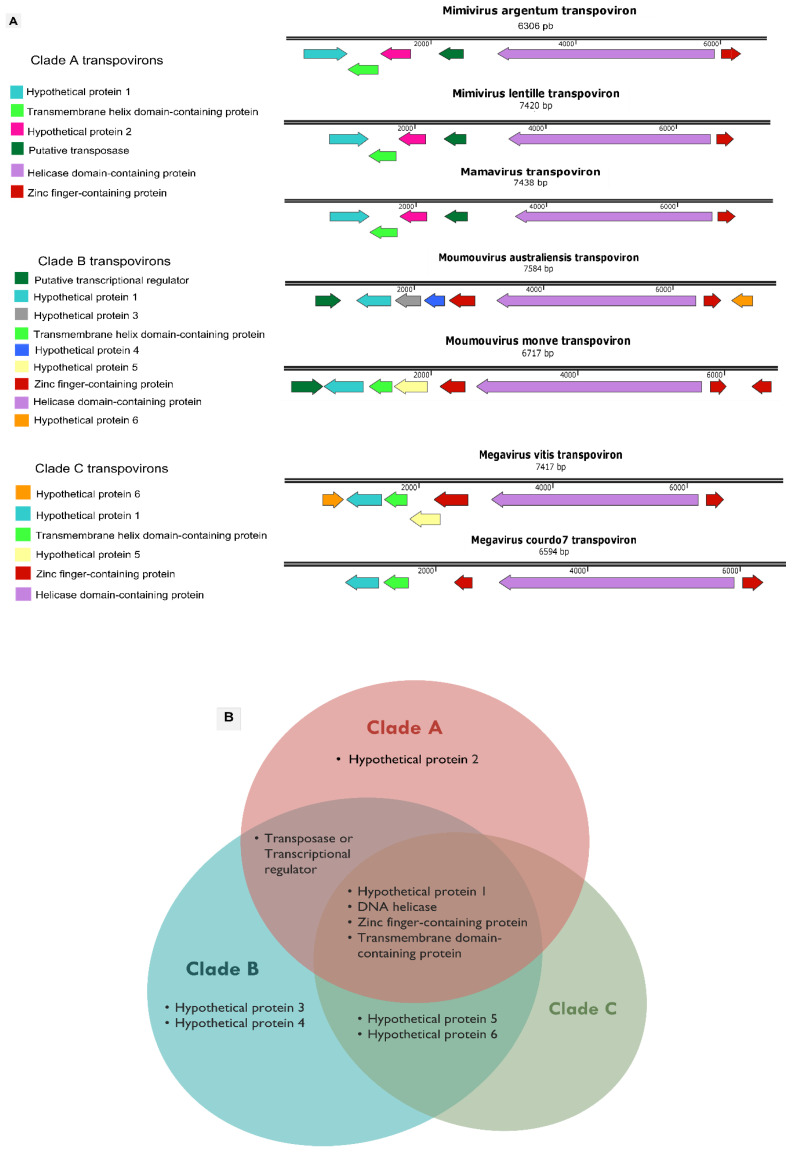
Genomic comparison between known transpoviron sequences. (**A**) Individual maps of the seven known transpovirons. Colored arrows indicate the ORFs distribution throughout genomes. Homolog ORFs are represented by the same color, and each predicted function is indicated in the color legend. Right-directed arrows represent positive strand ORFs and left-directed arrows represent negative strand ORFs. (**B**) Venn diagram summarizing the transpovirons proteins occurrence in each of the three clades (A, B and C). Note: megavirus courdo7 transpoviron is described to encode six ORFs; however, only five were found in the NCBI database. The transpoviron sequences were obtained through the following GenBank accession numbers: JQ063128.1 (mamavirus transpoviron); JQ063127.1 (mimivirus lentille transpoviron), MG807317.1 (moumouvirus australiensis transpoviron), JQ063129.1 (moumouvirus monve transpoviron), MG807316.1 (megavirus vitis transpoviron), and JQ063126.1 (megavirus courdo7 transpoviron).

All known transpovirons were described to form three phylogenetic clades (A, B, and C) according to the mimivirus lineage they are associated with [28]. Despite the mentioned differences, the mimivirus argentum transpoviron presents a gene organization and content that matches two clade A transpovirons (Figure 5A). By analyzing the occurrence of homologous proteins in each clade, it was observed that the seven known transpovirons encode 10 different proteins. Six of them are considered hypothetical proteins (numbered 1 to 6), whereas the other four present some predicted function and/or domain (Figure 5A). This analysis is summarized in Figure 5B, in which the characteristics of the protein distribution among transpovirons clades can be observed. Clades A and B have exclusive proteins, i.e., hypothetical protein 2 in clade A and hypothetical proteins 3 and 4 in clade B. Clade B transpovirons seem to encode more genes and possess a higher number of different proteins in comparison with the other clades. In addition, clade B shares exclusive homologous proteins with clade A (transposase/transcriptional regulator) and with clade C (hypothetical proteins 5 and 6).

**Figure 6:**
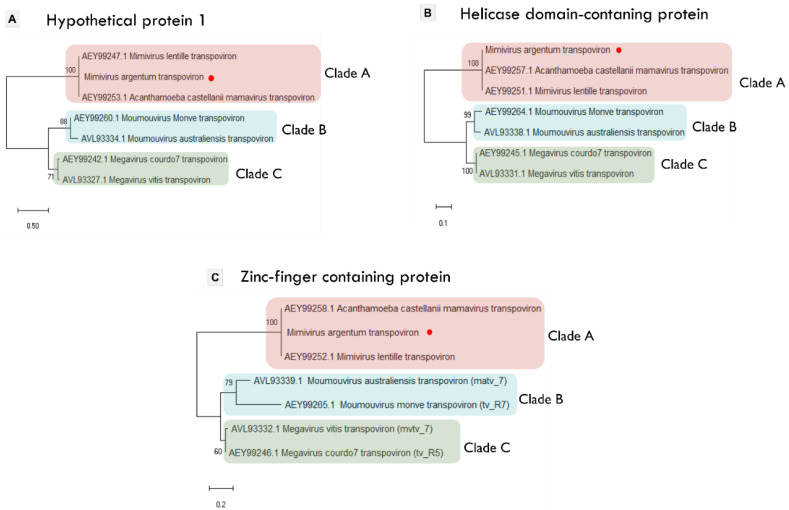
Phylogeny of three transpovirons core proteins. (**A**–**C**) Maximum-likelihood trees based on amino acid sequences from a hypothetical protein (ORF1), helicase domain-containing protein (ORF5), and zinc-finger containing protein (ORF6), respectively. The best-fit models chosen with ModelFinder (implemented in IQtree) for trees A, B, and C was JTTDCMut + F + G4, JTT + F + I, and VT, respectively. Mimivirus argentum transpoviron sequences are indicated by the red circle. Tree scale bars represent the number of amino acid substitutions per site.

In the center of the Venn diagram (Figure 5B), the four core proteins of transpovirons clades can be observed. Three of these proteins (hypothetical protein 1, helicase domain-containing protein, and zinc finger-containing protein) are considered highly conserved since they are present in all the transpoviron sequences, as described before [18,28]. Although the transmembrane domain-containing protein has no homologs in the moumouvirus australiensis transpoviron, it is present in all three clades (Figure 5A). The topology of the three transpoviron clades is represented in the phylogenetic trees based on the three most conserved transpoviron proteins mentioned above (Figure 6). Mimivirus argentum transpoviron clusters with the other clade A transpovirons. Additionally, we performed a phylogenetic analysis with two other mimivirus argentum transpoviron proteins, reinforcing its association with the previously described clade A transpovirons (Figure S4). Although the transmembrane domain-containing protein can be found in all three clades, the phylogenetic analysis did not conserve the topology of the clades except for clade A (Figure S4). Altogether, these phylogenetic analyses suggest that the clade A transpovirons can be considered highly conserved since they are maintained in all phylogenetic trees and encode an exclusive protein. On the other hand, the discovery of new transpoviron sequences could change this scenario. Moreover, the separation of transpovirons into clades that correspond with mimivirus lineages suggests that the relationship between transpovirons and mimiviruses may be ancient.

## 4. Conclusions

The first description of a mimivirus triggered the discovery of several groups of giant viruses, evidencing the wide diversity and ubiquity of these unique components of the virosphere. Giant virus prospecting studies are important because they facilitate the establishment of the basic biology of these viruses. In this context, we obtained an overview of the isolated particles by TEM images, which was useful for identification and particle morphology characterization and to observe some aspects of the mimivirus-virophage replication cycle. These observations raised interest in deeper investigations about some mechanisms that are still not completely established, especially regarding the virophage replication cycle. Thus, more detailed studies considering, for example, different post-infection time points, could be helpful to expand the knowledge of this topic. Most of the virophages known to date as well as some mimiviruses and transpovirons are described from metagenomic or genomic studies. Thus, the isolation of mimivirus argentum and its associated virophage suggests that research into the isolation of virophages around the world can be successful. Although metagenomic and genomic studies are extremely important, the isolation of a virus and, eventually, its associated elements allow virological and microscopical analysis, which is not expected when only metagenomic sequences are available.

We performed an extensive genomic characterization, which allowed for the detection of a transpoviron, the third member of the described tripartite system related to mimiviruses. Additionally, this characterization enabled the prediction of putative protein functions for all three analyzed sequences although these predictions need to be confirmed through deeper bioinformatics analysis and experimental investigations. The genomic characteristics of mimivirus argentum and its virophage and transpoviron are very similar to what has been described for other known sequences, suggesting the existence of selective pressure to conserve the genomic interaction in this tripartite system throughout evolution. Nevertheless, the detection of a new transpoviron sequence is especially interesting because even with the constant expansion of the *Nucleocytoviricota* phylum, there are still only a few reports of this type of element associated with giant viruses and virophages. Transpovirons and virophages have been described mostly in association with *Mimiviridae* family members, and this work reinforces that this relationship may be more common than expected. Therefore, the discovery and characterization of new giant viruses associated with such unique and little-known elements in different locations of the world (e.g., Brazil) can contribute to a better understanding about the diversity and distribution of these entities as well as their interaction with mimiviruses. It is notable that this triple relationship seems to be complex and the result of a long evolutionary history, representing an important source of knowledge that still needs to be explored.

## Figures and Tables

**Table 1:** Primer sequences used in PCR assays.

Target	Forward	Reverse	Expected Amplicon Sizes
Initial integration region (IIR)	5′ TATCACCCTTAGTACCCTTG 3′	5′ GCAGTGACAAAATACCCATT 3′	778 bp
Final integration region (FIR)	5′ CCACAATTAGGGCATTCAC 3′	5′ GGAAGCGAAGGTATTAAAGG 3′	889 bp
Virophage’s ORF 12	5′ GCATACTGAAGAGAGTGCCG 3′	5′ AGGAAAAGAAAGAGGAACACCAG 3′	574 bp

## Data Availability

Genome sequences obtained in this work are deposited in GenBank (NCBI) under the following accession numbers: OL770070 (Mimivirus argentum); OL770071 (Sputnik argentum); OL770072 (Mimivirus argentum-associated transpoviron).

## Supplementary Materials

The following supporting information can be downloaded at: https://www.mdpi.com/article/10.3390/v14020206/s1. Figure S1: Phylogeny of mimivirus argentum using different data sets. Figure S2: Phylogeny of three virophage core proteins. Figure S3: Detection of a provirophage associated to mimivirus argentum genome only during bioinformatics analysis. Figure S4: Phylogenetic trees based on two transpovirons proteins. Table S1: Detailed annotation of mimivirus argentum genome through BLASTp analysis. Table S2: Detailed annotation of sputnik argentum genome through BLASTp and HHpred analysis. Table S3: Detailed annotation of mimivirus argentum transpoviron through BLASTp and HHpred analysis.
