# Supplementary material for: The Discovery of a New Mimivirus Isolate in Association with Virophage-Transpoviron Elements in Brazil Highlights the Main Genomic and Evolutionary Features of This Tripartite System
Source: Viruses. 2022 Jan 21;14(2):206. doi: 10.3390/v14020206 (PMC8877843; doi:10.3390/v14020206)
Supplement: Supplementary file 1 [file viruses-14-00206-s001.zip › viruses-1532156-supplementary figures.pdf]

viruses

MDPI

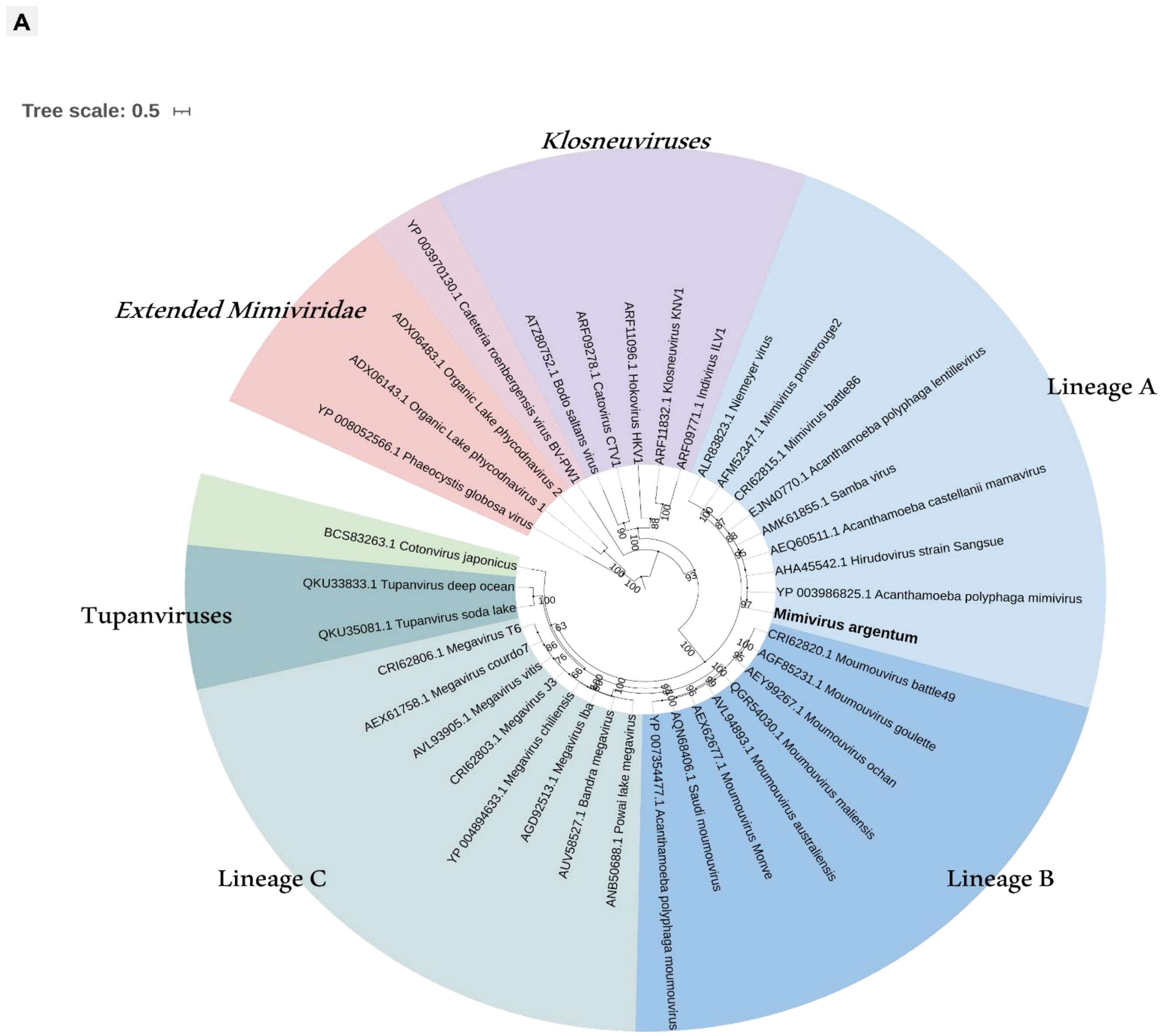

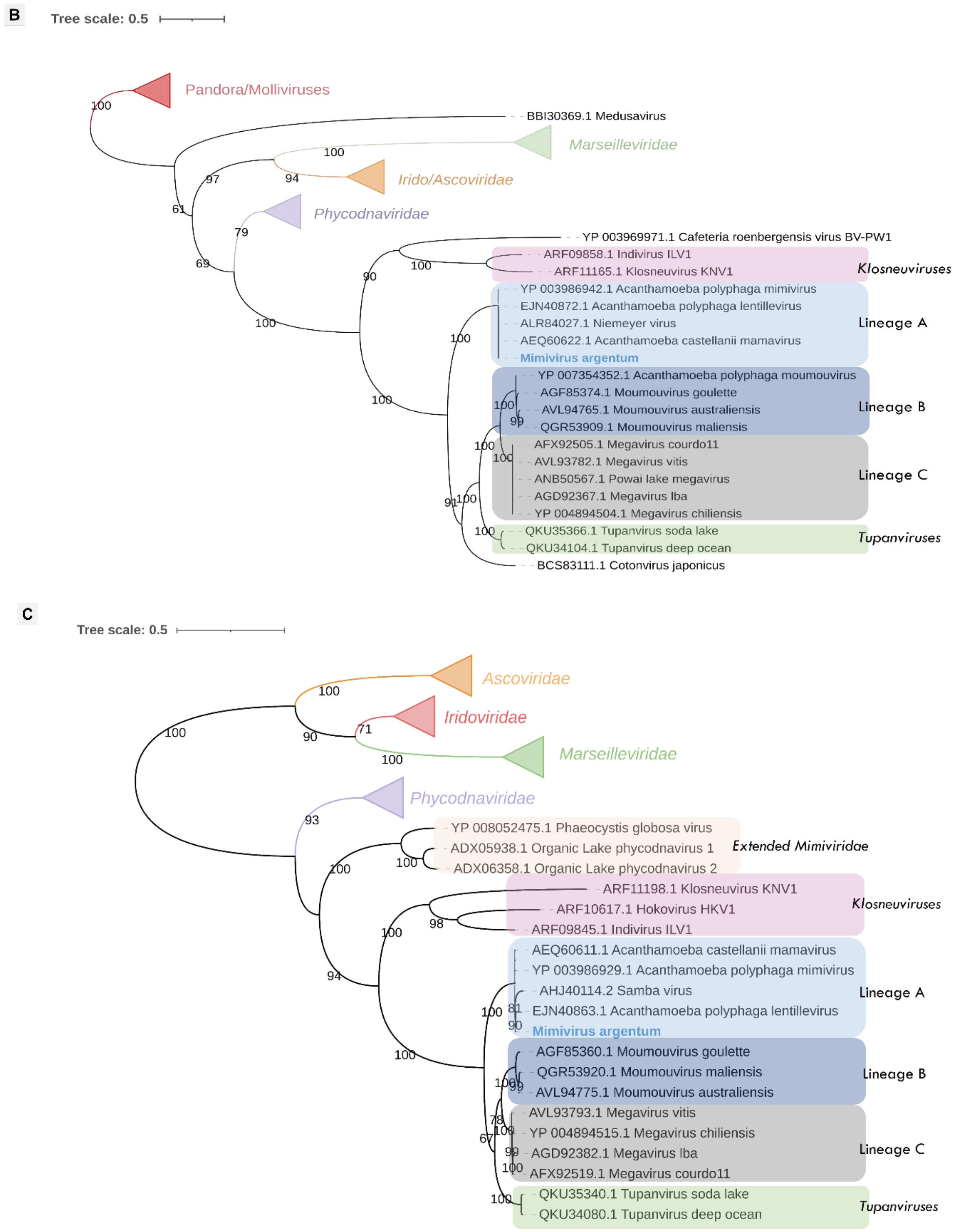

Figure S1. Phylogeny of mimivirus argentum using different data sets. **(A)** *Mimiviridae* maximum-likelihood tree based on DNA polymerase B (ORF678) amino acid sequence. **(B-C)** Maximum-likelihood trees based on VV A32 virion packaging ATPase (ORF539) and major capsid protein (ORF552), respectively. The best-fit models chosen with ModelFinder (implemented in IQtree) for trees A, B and C were LG+F+I+G4, LG+F+I+G4, and VT+F+I+G4 respectively. Mimivirus argentum sequences are labeled with bold and/or blue font. Scale bars represent the number of amino acid substitutions per site.

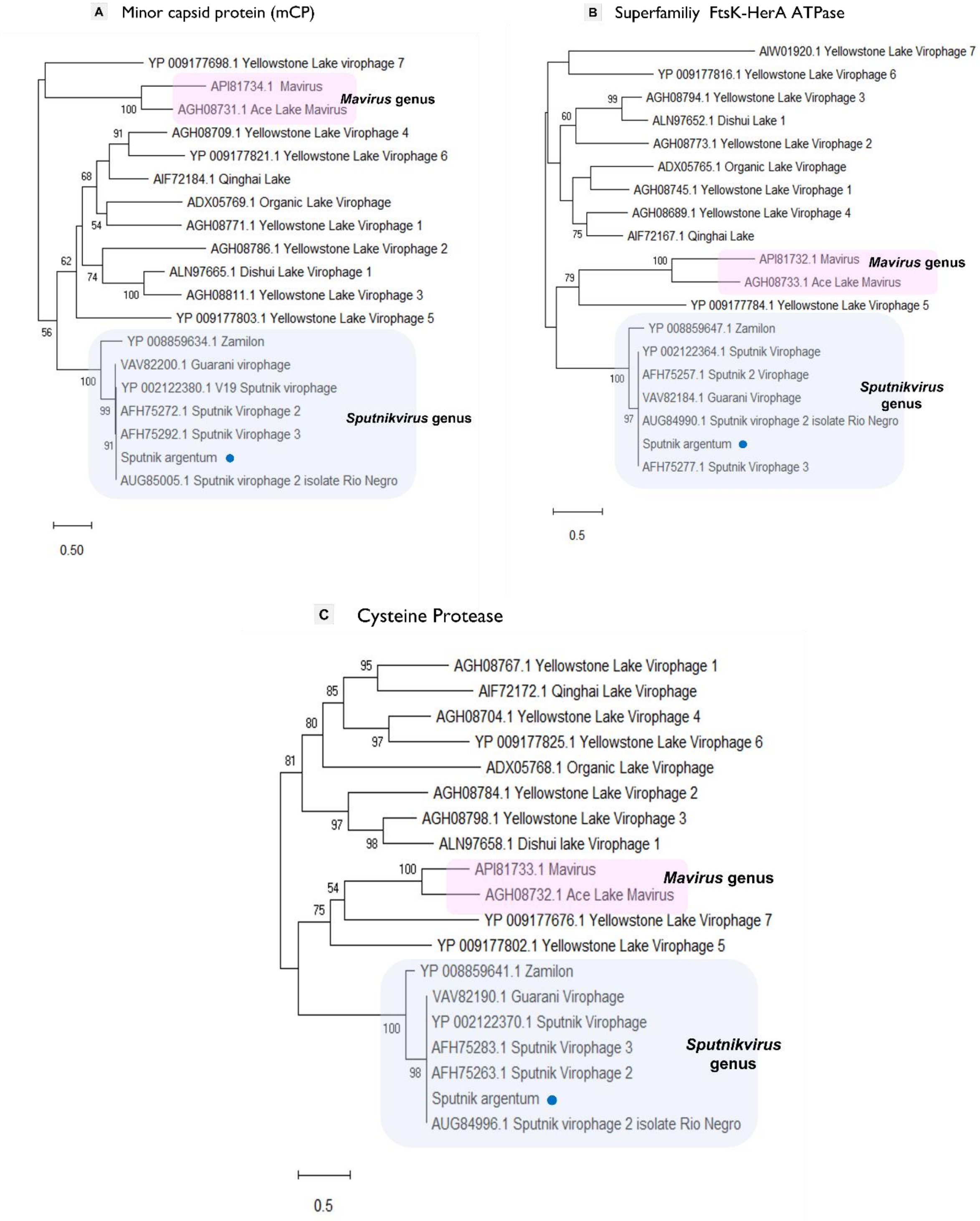

Figure S2. Phylogeny of three virophage core proteins. **(A-C)** Maximum-likelihood trees based on minor capsid "penton" protein (mCP) (ORF18), superfamily FtsK-HerA ATPase (ORF3) and putative cysteine protease (ORF9) amino acid sequences. The best-fit models chosen with ModelFinder (implemented in IQtree) for trees A, B and C were T+F+I+G4; rtREV+F+I+G4, and LG+F+R3, respectively. Sputnik argentum sequences are indicated with the blue circle. Scale bars represent the number of amino acid substitutions per site.

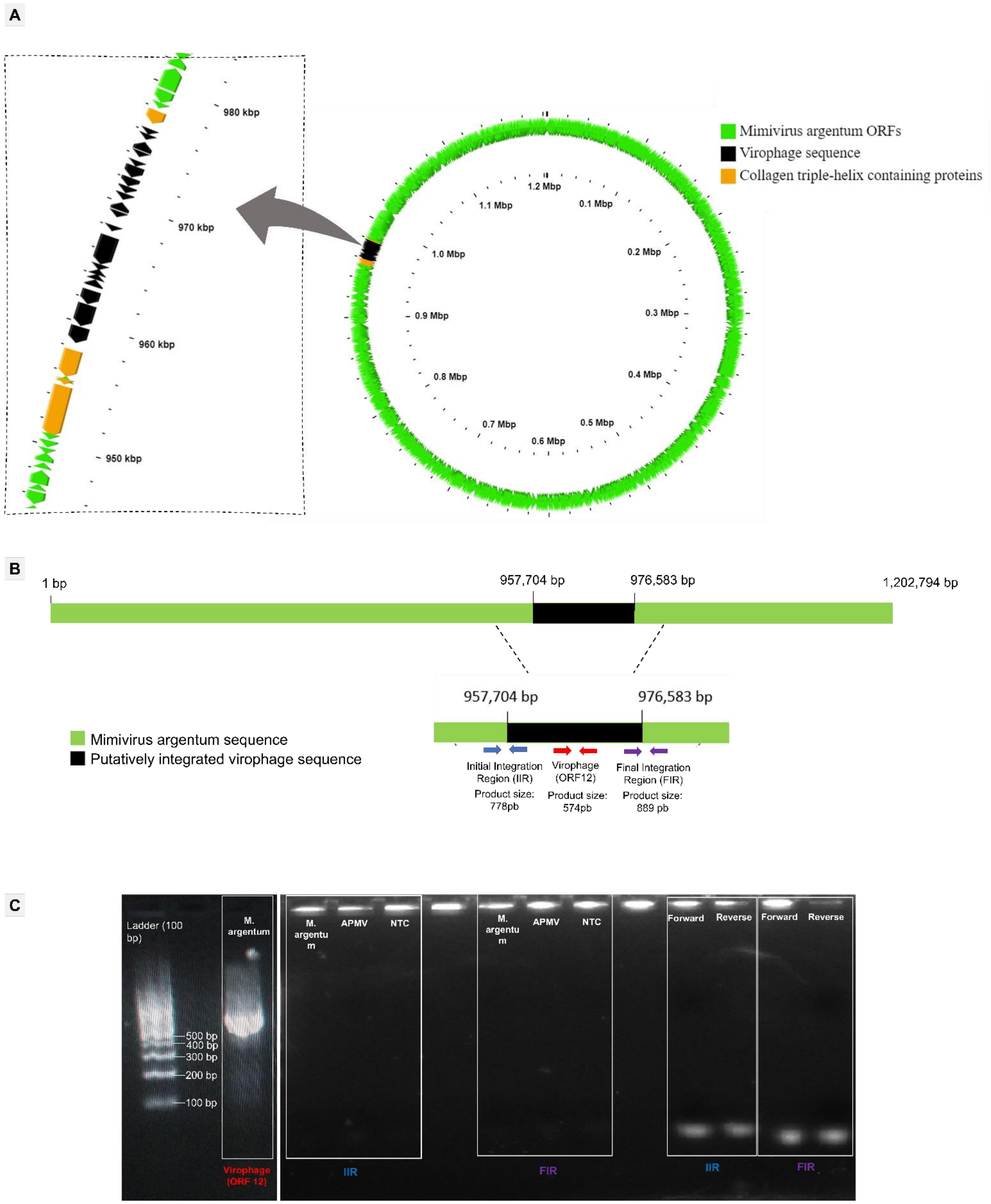

Figure S3. Detection of a provirophage associated with the mimivirus argentum genome only during bioinformatics analysis. **(A)** Representative map of the provirophage putative integration site in mimivirus argentum genome. **(B)** Scheme illustrating the mimivirus argentum genome region used as target in PCR analysis. The green block represents the mimivirus argentum genome, whereas the black block represents the putatively integrated virophage sequence. Arrows represent the targets used for primer design. Blue arrows indicate primers for the Initial Integration Region (IIR), purple arrows indicate primers for the Final

Integration Region (FIR) and red arrows indicate primers targeting ORF12 encoded by sputnik argentum. Right-directed arrows indicate forward primers and the left-directed ones indicate reverse primers. **(C)** Agarose electrophoresis gel after the PCR reaction using DNA extracted from the mimivirus-virophage containing sample. It is possible to observe an amplification with the primer targeted to the virophage's ORF12 and no amplifications with primers targeting both ends of the putative virophage integration site (IIR and FIR), suggesting the absence of a provirophage. The sample with diluted primer pairs (IIR and FIR) used in the PCR reaction was added to the gel as a reaction control, as observed in the amplifications at the bottom right. DNA extracted from Acanthamoeba polyphaga mimivirus (APMV) purified particles (known negative sample for provirophage) was used as the negative control. NTC: no template control.

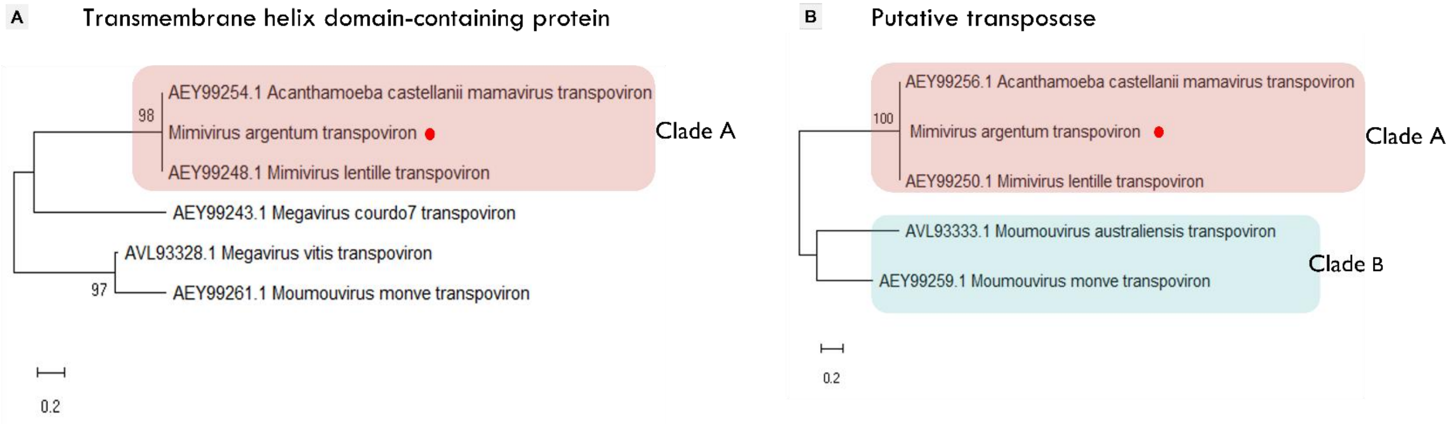

Figure S4. Phylogenetic trees based on two transpoviron proteins. **(A-B)** Maximum-likelihood based on mimivirus argentum-associated transpoviron transmembrane helix domain-containing protein (ORF2) and putative transposase (ORF4), respectively. The best-fit models chosen with ModelFinder (implemented in IQtree) for trees A and B were VT+F and Blosum62+F, respectively. Mimivirus argentum transpoviron sequences are labeled with a red circle. Scale bars represent the number of amino acid substitutions per site.
