# Supplementary material for: The Discovery of a New Mimivirus Isolate in Association with Virophage-Transpoviron Elements in Brazil Highlights the Main Genomic and Evolutionary Features of This Tripartite System
Source: Viruses. 2022 Jan 21;14(2):206. doi: 10.3390/v14020206 (PMC8877843; doi:10.3390/v14020206)
Supplement: Supplementary file 1 [file viruses-14-00206-s001.zip › viruses-1532156-supplementary tables.pdf]

Table S1 - Detailed annotation of mimivirus argentum genome through BLASTp analysis

ORF	Size (aa)	Start	Stop	Strand	Best hit BLASTp (nr)	Score	Query cover	e-value	Identity
1	231	2	694	-	hypothetical protein [Mimivirus reunion]	379	100%	5,00E-128	87.50%
2	218	1131	1787	-	hypothetical protein MIMI_gp0006 [Acanthamoeba polyphaga mimivirus]	444	100%	3,00E-157	100.00%
3	155	2764	3231	-	hypothetical protein MIMI_gp0007 [Acanthamoeba polyphaga mimivirus]	314	100%	5,00E-108	100.00%
4	1047	3383	6526	+	Helicase [Acanthamoeba castellanii mamavirus]	2117	100%	0.0	100.00%
5	376	6886	8016	+	hypothetical protein MIMI_gp0009 [Acanthamoeba polyphaga mimivirus]	762	100%	0.0	100.00%
6	376	8328	9458	+	T5orf172 domain-containing protein [Acanthamoeba castellanii mamavirus]	754	100%	0.0	100.00%
7	85	9633	9890	-	uncharacterized Bro-N domain-containing protein [Acanthamoeba polyphaga mimivirus]	171	100%	2,00E-53	100.00%
8	232	9930	10628	+	Uncharacterized protein R11 [Acanthamoeba polyphaga mimivirus]	469	100%	2,00E-166	100.00%
9	84	10755	11009	+	hypothetical protein MIMI_gp0013 [Acanthamoeba polyphaga mimivirus]	176	100%	2,00E-55	100.00%
10	90	11312	11584	+	putative replication origin-binding protein [Hirudovirus strain Sangsue]	180	100%	6,00E-57	98.89%
11	487	11773	13236	-	hypothetical protein MIMI_gp0014 [Acanthamoeba polyphaga mimivirus]	970	100%	0.0	100.00%
12	183	13598	14149	+	hypothetical protein MIMI_gp0015 [Acanthamoeba polyphaga mimivirus]	358	100%	1,00E-124	100.00%
13	67	14196	14399	+	hypothetical protein HIRU_S992 [Hirudovirus strain Sangsue]	134	100%	3,00E-39	100.00%
14	467	15021	16424	-	putative ankyrin repeat protein [Acanthamoeba polyphaga mimivirus]	927	100%	0.0	100.00%
15	60	16484	16666	-	hypothetical protein MIMI_gp0976 [Acanthamoeba polyphaga mimivirus]	122	100%	5,00E-35	100.00%
16	210	16738	17370	+	hypothetical protein MIMI_gp0975 [Acanthamoeba polyphaga mimivirus]	430	100%	3,00E-152	100.00%
17	431	17602	18897	+	hypothetical protein MIMI_gp0974 [Acanthamoeba polyphaga mimivirus]	874	100%	0.0	100.00%
18	62	18961	19149	-	hypothetical protein MIMI_gp0973 [Acanthamoeba polyphaga mimivirus]	114	100%	1,00E-31	100.00%
19	132	19306	19704	+	hypothetical protein MIMI_gp0972 [Acanthamoeba polyphaga mimivirus]	273	100%	1,00E-92	100.00%
20	196	19963	20553	-	hypothetical protein [Acanthamoeba castellanii mamavirus]	399	100%	3,00E-140	100.00%
21	579	20701	22440	+	Acetylcholinesterase [Acanthamoeba castellanii mamavirus]	1207	100%	0.0	100.00%
22	70	22566	22778	-	putative membrane protein [Acanthamoeba castellanii mamavirus]	135	100%	1,00E-39	100.00%
23	210	22837	23469	+	putative Fe2OG oxygenase family oxidoreductase [Acanthamoeba polyphaga mimivirus]	416	100%	3,00E-146	100.00%
24	343	23629	24660	-	KilA N-domain protein [Acanthamoeba castellanii mamavirus]	689	100%	0.0	100.00%
25	52	24775	24933	+	orphan [Mimivirus reunion]	104	100%	6,00E-28	100.00%
26	365	24988	26085	-	putative ankyrin repeat protein [Acanthamoeba polyphaga mimivirus]	741	100%	0.0	100.00%
27	213	26369	27010	-	hypothetical protein MIMI_gp0966 [Acanthamoeba polyphaga mimivirus]	414	100%	2,00E-145	100.00%
28	361	27552	28637	-	putative ankyrin repeat protein [Acanthamoeba polyphaga mimivirus]	720	100%	0.0	100.00%
29	109	28697	29026	-	hypothetical protein HIRU_S15 [Hirudovirus strain Sangsue]	215	100%	2,00E-70	99.08%
30	54	29203	29367	-	hypothetical protein [Acanthamoeba castellanii mimivirus]	114	100%	6,00E-32	100.00%
31	101	29457	29762	-	hypothetical protein MIMI_gp0964 [Acanthamoeba polyphaga mimivirus]	207	100%	3,00E-67	100.00%
32	161	29916	30401	+	hypothetical protein MIMI_gp0963 [Acanthamoeba polyphaga mimivirus]	319	100%	9,00E-110	100.00%
33	201	30491	31096	-	hypothetical protein [Acanthamoeba castellanii mamavirus]	402	100%	3,00E-141	100.00%
34	347	31154	32197	+	putative ankyrin repeat protein [Acanthamoeba polyphaga mimivirus]	637	92%	0.0	100.00%
35	332	32330	33328	-	putative ankyrin repeat protein [Acanthamoeba polyphaga mimivirus]	682	100%	0.0	100.00%
36	698	33440	35536	-	putative endonuclease/exonuclease/phosphatase [Acanthamoeba polyphaga mimivirus]	1450	100%	0.0	100.00%
37	702	35791	37899	+	GMC oxidoreductase [Acanthamoeba castellanii mamavirus]	1449	100%	0.0	100.00%
38	269	38058	38867	-	hypothetical protein MIMI_gp0957 [Acanthamoeba polyphaga mimivirus]	555	100%	0.0	100.00%
39	104	39054	39368	+	hypothetical protein MIMI_gp0956 [Acanthamoeba polyphaga mimivirus]	198	100%	9,00E-64	100.00%
40	180	39453	39995	-	hypothetical protein MIMI_gp0955 [Acanthamoeba polyphaga mimivirus]	357	100%	5,00E-124	100.00%
41	114	40091	40435	-	hypothetical protein MIMI_gp0954 [Acanthamoeba polyphaga mimivirus]	231	100%	2,00E-76	100.00%
42	193	40436	41017	-	ankyrin repeat-containing protein [Acanthamoeba castellanii mamavirus]	392	100%	8,00E-138	99.48%
43	271	41073	41888	+	hypothetical protein MIMI_gp0953 [Acanthamoeba polyphaga mimivirus]	541	100%	0.0	100.00%
44	155	41959	42426	+	Transcription factor jumonji [Acanthamoeba castellanii mamavirus]	312	100%	3,00E-107	100.00%
45	428	42492	43778	+	putative JmjC domain-containing protein [Acanthamoeba polyphaga mimivirus]	858	100%	0.0	100.00%
46	72	43867	44085	-	putative ankyrin repeat protein [Hirudovirus strain Sangsue]	140	100%	6,00E-42	98.61%
47	144	44235	44669	+	hypothetical protein MIMI_gp0951 [Acanthamoeba polyphaga mimivirus]	286	100%	2,00E-97	100.00%
48	323	45082	46053	+	hypothetical protein [Samba virus]	653	100%	0.0	100.00%
49	452	46125	47483	-	hypothetical protein MIMI_gp0949 [Acanthamoeba polyphaga mimivirus]	912	100%	0.0	100.00%
50	538	47530	49146	-	hypothetical protein MIMI_gp0948 [Acanthamoeba polyphaga mimivirus]	1066	100%	0.0	100.00%
51	187	49338	49901	+	hypothetical protein MIMI_gp0947 [Acanthamoeba polyphaga mimivirus]	384	100%	1,00E-134	100.00%
52	255	50193	50960	-	putative ankyrin repeat protein [Acanthamoeba polyphaga mimivirus]	520	100%	0.0	100.00%
53	483	51114	52565	-	KilA N-terminal domain, N1R/P28 DNA binding protein [Acanthamoeba polyphaga lentillevirus]	974	100%	0.0	100.00%
54	171	52672	53187	-	putative outer membrane lipoprotein [Acanthamoeba castellanii mamavirus]	342	100%	2,00E-118	100.00%
55	412	53317	54555	+	hypothetical protein [Acanthamoeba castellanii mamavirus]	820	100%	0.0	100.00%
56	317	54629	55582	-	ankyrin repeat-containing protein [Acanthamoeba castellanii mamavirus]	638	100%	0.0	100.00%
57	178	55861	56397	-	hypothetical protein [Acanthamoeba castellanii mamavirus]	366	100%	8,00E-128	100.00%
58	468	56480	57886	-	ankyrin repeat-containing protein [Acanthamoeba castellanii mamavirus]	942	100%	0.0	100.00%
59	340	58133	59155	+	hypothetical protein [Acanthamoeba castellanii mamavirus]	677	100%	0.0	100.00%
60	76	59233	59463	+	hypothetical protein [Acanthamoeba castellanii mamavirus]	154	100%	5,00E-47	100.00%
61	575	59567	61294	-	hypothetical protein [Acanthamoeba castellanii mamavirus]	1172	100%	0.0	100.00%
62	64	61369	61563	-	hypothetical protein [Acanthamoeba castellanii mamavirus]	125	100%	3,00E-36	100.00%
63	389	61712	62881	+	hypothetical protein [Acanthamoeba castellanii mamavirus]	758	98%	0.0	100.00%
64	426	62937	64217	-	ankyrin repeat-containing protein [Acanthamoeba castellanii mamavirus]	861	100%	0.0	100.00%
65	201	64309	64914	-	ankyrin repeat-containing protein [Acanthamoeba castellanii mamavirus]	415	100%	2,00E-146	100.00%
66	65	65139	65336	-	hypothetical protein [Mimivirus reunion]	133	100%	4,00E-39	98.46%
67	247	65783	66526	-	hypothetical protein [Acanthamoeba castellanii mamavirus]	499	100%	5,00E-178	100.00%
68	191	66590	67165	-	BTB super family protein [Acanthamoeba castellanii mamavirus]	379	100%	1,00E-132	100.00%
69	677	67211	69244	-	hypothetical protein HIRU_S57 [Hirudovirus strain Sangsue]	1365	100%	0.0	100.00%
70	299	70282	71181	+	putative ankyrin repeat protein [Acanthamoeba polyphaga mimivirus]	594	100%	0.0	100.00%
71	514	71284	72828	+	putative ankyrin repeat protein [Acanthamoeba polyphaga mimivirus]	1027	100%	0.0	100.00%
72	310	73244	74176	+	hypothetical protein [Samba virus]	624	100%	0.0	100.00%
73	212	74240	74878	-	hypothetical protein MIMI_gp0922 [Acanthamoeba polyphaga mimivirus]	428	100%	3,00E-151	100.00%
74	106	74963	75283	-	hypothetical protein MIMI_gp0921 [Acanthamoeba polyphaga mimivirus]	214	100%	7,00E-70	100.00%
75	315	75478	76425	+	hypothetical protein MIMI_gp0920 [Acanthamoeba polyphaga mimivirus]	658	100%	0.0	100.00%
76	424	76490	77764	-	ankyrin repeat-containing protein [Acanthamoeba castellanii mamavirus]	859	100%	0.0	100.00%
77	180	77840	78382	-	hypothetical protein MIMI_gp0918 [Acanthamoeba polyphaga mimivirus]	371	100%	9,00E-130	100.00%
78	342	78682	79710	-	putative TPR repeat-containing protein [Acanthamoeba polyphaga mimivirus]	693	100%	0.0	100.00%
79	123	79829	80200	-	hypothetical protein lvs_R749 [Acanthamoeba polyphaga lentillevirus]	254	100%	2,00E-85	100.00%
80	241	80314	81039	-	putative transposase [Niemeyer virus]	468	98%	1,00E-164	100.00%
81	393	81103	82284	-	putative deoxyribodipyrimidine photolyase-related protein [Acanthamoeba polyphaga mimivirus]	793	100%	0.0	100.00%
82	100	82315	82617	+	hypothetical protein MIMI_gp0913 [Acanthamoeba polyphaga mimivirus]	204	100%	3,00E-66	100.00%
83	513	82675	84216	-	putative sel1-like repeat-containing protein [Acanthamoeba polyphaga mimivirus]	1035	100%	0.0	100.00%
84	141	84289	84714	-	hypothetical protein MIMI_gp0911 [Acanthamoeba polyphaga mimivirus]	288	100%	3,00E-98	100.00%
85	519	84781	86340	-	ankyrin repeat-containing protein [Acanthamoeba castellanii mamavirus]	1047	100%	0.0	100.00%
86	100	86403	86705	-	ankyrin repeat protein [Samba virus]	201	100%	4,00E-65	100.00%
87	545	87035	88672	-	putative transposase [Acanthamoeba castellanii mamavirus]	1114	100%	0.0	100.00%
88	191	88779	89354	+	putative site-specific integrase-resolvase [Acanthamoeba castellanii mamavirus]	381	100%	3,00E-133	100.00%
89	141	89402	89827	-	ankyrin repeat-containing protein [Acanthamoeba castellanii mamavirus]	279	100%	1,00E-94	100.00%
90	440	89886	91208	-	putative ankyrin repeat protein [Acanthamoeba polyphaga mimivirus]	899	100%	0.0	100.00%
91	312	91261	92199	-	Esterase lipase superfamily protein [Acanthamoeba castellanii mamavirus]	642	100%	0.0	100.00%

92	85	92382	92639	-	hypothetical protein [Acanthamoeba castellanii mamavirus]	165	100%	3,00E-51	100.00%
93	504	92593	94107	-	putative BTB/POZ domain-containing protein [Acanthamoeba polyphaga mimivirus]	1000	100%	0.0	100.00%
94	601	94246	96051	-	putative ankyrin repeat protein [Acanthamoeba polyphaga mimivirus]	1197	100%	0.0	100.00%
95	526	96201	97781	-	ankyrin repeat-containing protein [Acanthamoeba castellanii mamavirus]	1058	100%	0.0	100.00%
96	457	97888	99261	-	hypothetical protein MIMI_gp0902 [Acanthamoeba polyphaga mimivirus]	927	100%	0.0	100.00%
97	276	99402	100232	-	ankyrin repeat-containing protein [Acanthamoeba castellanii mamavirus]	563	100%	0.0	100.00%
98	626	100349	102229	-	putative ankyrin repeat protein [Acanthamoeba polyphaga mimivirus]	1251	100%	0.0	100.00%
99	148	102311	102757	-	hypothetical protein [Acanthamoeba castellanii mamavirus]	297	100%	1,00E-101	100.00%
100	308	102812	103738	-	ankyrin repeat-containing protein [Acanthamoeba castellanii mamavirus]	613	100%	0.0	100.00%
101	262	103874	104662	+	putative BTB/POZ domain-containing protein [Acanthamoeba polyphaga mimivirus]	523	100%	0.0	100.00%
102	553	104787	106448	-	glucose-methanol-choline oxidoreductase [Acanthamoeba polyphaga mimivirus]	1139	100%	0.0	100.00%
103	1624	106701	111575	-	putative serine/threonine-protein kinase/receptor [Acanthamoeba polyphaga mimivirus]	3356	100%	0.0	100.00%
104	477	111752	113185	-	putativeBTB/POZ domain-containing protein [Acanthamoeba polyphaga mimivirus]	963	100%	0.0	100.00%
105	433	113308	114609	+	hypothetical protein [Acanthamoeba castellanii mamavirus]	900	100%	0.0	100.00%
106	78	114719	114955	+	hypothetical protein MIMI_gp0892 [Acanthamoeba polyphaga mimivirus]	165	100%	2,00E-51	100.00%
107	124	115013	115387	-	hypothetical protein [Acanthamoeba castellanii mamavirus]	249	100%	3,00E-83	100.00%
108	297	115539	116432	-	hypothetical protein MIMI_gp0890 [Acanthamoeba polyphaga mimivirus]	607	100%	0.0	100.00%
109	1638	116584	121500	-	putative serine/threonine-protein kinase/receptor [Acanthamoeba polyphaga mimivirus]	3401	100%	0.0	100.00%
110	311	121717	122652	-	putative ankyrin repeat protein [Acanthamoeba polyphaga mimivirus]	631	100%	0.0	100.00%
111	221	122712	123377	-	putative 5'(3')-deoxyribonucleotidase [Acanthamoeba polyphaga mimivirus]	452	100%	3,00E-160	100.00%
112	323	123527	124498	+	putative proliferating cell nuclear antigen [Acanthamoeba polyphaga mimivirus]	644	100%	0.0	100.00%
113	568	124599	126305	-	hypothetical protein MIMI_gp0885 [Acanthamoeba polyphaga mimivirus]	1183	100%	0.0	100.00%
114	556	126447	128117	-	hypothetical protein MIMI_gp0884 [Acanthamoeba polyphaga mimivirus]	1125	100%	0.0	100.00%
115	120	128237	128599	-	hypothetical protein MIMI_gp0883 [Acanthamoeba polyphaga mimivirus]	251	100%	5,00E-84	100.00%
116	438	128776	130092	-	hypothetical protein MIMI_R819 [Acanthamoeba polyphaga mimivirus]	902	100%	0.0	100.00%
117	1651	130241	135196	-	putative serine/threonine-protein kinase/receptor [Acanthamoeba polyphaga mimivirus]	3415	100%	0.0	100.00%
118	142	135477	135905	+	hypothetical protein [Samba virus]	286	100%	3,00E-97	100.00%
119	198	135967	136563	-	hypothetical protein [Acanthamoeba castellanii mamavirus]	404	100%	2,00E-142	100.00%
120	540	136647	138269	-	putative sel1-like repeat-containing protein [Acanthamoeba polyphaga mimivirus]	1104	100%	0.0	100.00%
121	305	138384	139301	-	hypothetical protein MIMI_gp0877 [Acanthamoeba polyphaga mimivirus]	628	100%	0.0	100.00%
122	102	139473	139781	+	hypothetical protein MIMI_gp0876 [Acanthamoeba polyphaga mimivirus]	192	100%	2,00E-61	100.00%
123	990	139958	142930	-	putative ariadne-like ring finger protein [Acanthamoeba polyphaga mimivirus]	2052	100%	0.0	100.00%
124	211	143016	143651	-	putative ankyrin repeat protein [Acanthamoeba polyphaga mimivirus]	421	100%	2,00E-148	100.00%
125	229	143733	144422	-	hypothetical protein MIMI_gp0872 [Acanthamoeba polyphaga mimivirus]	467	100%	6,00E-166	100.00%
126	169	144545	145054	+	cytochrome P450 family protein [Acanthamoeba castellanii mamavirus]	344	100%	2,00E-119	100.00%
127	543	145044	146675	+	probable lanosterol 14-alpha demethylase [Acanthamoeba polyphaga mimivirus]	1135	100%	0.0	99.82%
128	447	146804	148147	-	probable 7-dehydrocholesterol reductase [Acanthamoeba polyphaga mimivirus]	915	100%	0.0	100.00%
129	108	148286	148612	+	hypothetical protein [Acanthamoeba castellanii mamavirus]	223	100%	2,00E-73	100.00%
130	192	148664	149242	+	hypothetical protein MIMI_gp0868 [Acanthamoeba polyphaga mimivirus]	388	100%	5,00E-136	100.00%
131	186	149299	149859	-	hypothetical protein MIMI_gp0867 [Acanthamoeba polyphaga mimivirus]	364	100%	1,00E-126	100.00%
132	243	150000	150731	+	putative metal-dependent phosphohydrolase, HD domain [Acanthamoeba polyphaga mimivirus]	506	100%	0.0	100.00%
133	247	150807	151550	-	putative purine phosphorylase [Acanthamoeba polyphaga mimivirus]	512	100%	0.0	100.00%
134	131	151694	152089	+	hypothetical protein MIMI_gp0864 [Acanthamoeba polyphaga mimivirus]	262	100%	3,00E-88	100.00%
135	114	152227	152571	-	hypothetical protein MIMI_gp0863 [Acanthamoeba polyphaga mimivirus]	228	100%	2,00E-75	100.00%
136	201	152649	153254	-	hypothetical protein [Acanthamoeba castellanii mamavirus]	390	96%	1,00E-136	100.00%
137	269	153394	154203	+	hypothetical protein lvs_L700 [Acanthamoeba polyphaga lentillevirus]	552	100%	0.0	100.00%
138	445	154217	155554	-	putative ankyrin repeat protein [Acanthamoeba polyphaga mimivirus]	897	100%	0.0	100.00%
139	142	155682	156110	+	hypothetical protein MIMI_gp0859 [Acanthamoeba polyphaga mimivirus]	296	100%	4,00E-101	100.00%
140	1297	156205	160098	-	RING-finger-containing E3 ubiquitin ligase [Acanthamoeba polyphaga mimivirus]	2667	100%	0.0	99.92%
141	363	160316	161407	+	DNA primase small subunit [Acanthamoeba castellanii mamavirus]	729	100%	0.0	100.00%
142	224	161495	162169	-	hypothetical protein [Acanthamoeba castellanii mamavirus]	458	100%	1,00E-162	100.00%
143	228	162314	163000	+	hypothetical protein MIMI_gp0854 [Acanthamoeba polyphaga mimivirus]	465	100%	2,00E-165	100.00%
144	287	163067	163930	-	putative ankyrin repeat protein [Acanthamoeba polyphaga mimivirus]	582	100%	0.0	100.00%
145	118	164027	164383	-	hypothetical protein HIRU_S132 [Hirudovirus strain Sangsue]	236	100%	3,00E-78	100.00%
146	422	164594	165862	-	putative ankyrin repeat protein [Acanthamoeba polyphaga mimivirus]	859	100%	0.0	100.00%
147	498	166025	167521	+	WD40 family protein [Acanthamoeba castellanii mamavirus]	1001	100%	0.0	100.00%
148	437	167599	168912	-	putative ankyrin repeat protein [Acanthamoeba polyphaga mimivirus]	895	100%	0.0	100.00%
149	492	169053	170531	+	putative BTB/POZ domain and WD-repeat protein [Acanthamoeba polyphaga mimivirus]	979	100%	0.0	100.00%
150	108	170600	170926	-	hypothetical protein MIMI_gp0847 [Acanthamoeba polyphaga mimivirus]	211	100%	7,00E-69	100.00%
151	433	170972	172273	-	putative ankyrin repeat protein [Acanthamoeba polyphaga mimivirus]	871	100%	0.0	100.00%
152	524	172382	173956	+	WD40 family protein [Acanthamoeba castellanii mamavirus]	1063	100%	0.0	100.00%
153	126	174025	174405	+	hypothetical protein [Acanthamoeba castellanii mamavirus]	259	100%	2,00E-87	100.00%
154	151	174652	175107	+	hypothetical protein MIMI_gp0843 [Acanthamoeba polyphaga mimivirus]	313	100%	7,00E-108	100.00%
155	289	175198	176067	+	uncharacterized dTDP-4-dehydrorhamnose reductase domain protein [Acanthamoeba polyphaga mimivirus]	602	100%	0.0	100.00%
156	103	176176	176487	+	hypothetical protein MIMI_gp0841 [Acanthamoeba polyphaga mimivirus]	208	100%	8,00E-68	100.00%
157	257	176588	177361	+	hypothetical protein MIMI_gp0840 [Acanthamoeba polyphaga mimivirus]	521	100%	0.0	100.00%
158	409	177393	178622	-	ankyrin-containing protein [Hirudovirus strain Sangsue]	827	100%	0.0	100.00%
159	84	178834	179088	-	putative chaperon protein DnaJ [Acanthamoeba polyphaga mimivirus]	172	100%	5,00E-54	100.00%
160	342	179180	180208	-	putative AAA-type ATPase [Acanthamoeba polyphaga mimivirus]	698	100%	0.0	100.00%
161	203	180364	180975	+	putative PAN domain-containing protein [Acanthamoeba polyphaga mimivirus]	416	100%	9,00E-147	100.00%
162	633	181059	182960	+	putative ankyrin repeat protein [Acanthamoeba polyphaga mimivirus]	1260	100%	0.0	100.00%
163	489	183059	184528	-	putative BTB/POZ domain-containing protein [Acanthamoeba polyphaga mimivirus]	995	100%	0.0	100.00%
164	295	184684	185571	+	hypothetical protein MIMI_gp0833 [Acanthamoeba polyphaga mimivirus]	594	100%	0.0	100.00%
165	190	185632	186204	-	putative resolvase [Acanthamoeba polyphaga mimivirus]	385	100%	6,00E-135	100.00%
166	520	186309	187871	+	putative transposase [Acanthamoeba polyphaga mimivirus]	1044	100%	0.0	99.42%
167	77	188119	188352	+	hypothetical protein MIMI_gp0830 [Acanthamoeba polyphaga mimivirus]	158	100%	1,00E-48	100.00%
168	628	188442	190328	+	putative ankyrin repeat protein [Acanthamoeba polyphaga mimivirus]	1248	100%	0.0	100.00%
169	626	190452	192332	+	putative ankyrin repeat protein [Acanthamoeba polyphaga mimivirus]	1241	100%	0.0	100.00%
170	632	192407	194305	+	putative ankyrin repeat protein [Acanthamoeba polyphaga mimivirus]	1248	100%	0.0	100.00%
171	636	194414	196324	+	putative ankyrin repeat protein [Acanthamoeba polyphaga mimivirus]	1255	100%	0.0	100.00%
172	473	196498	197919	-	putative BTB/POZ domain-containing protein [Acanthamoeba polyphaga mimivirus]	949	100%	0.0	100.00%
173	647	198086	200029	+	hypothetical protein lvs_L667 [Acanthamoeba polyphaga lentillevirus]	1266	100%	0.0	100.00%
174	262	200140	200928	+	hypothetical protein [Acanthamoeba castellanii mamavirus]	535	100%	0.0	100.00%
175	251	201009	201764	-	hypothetical protein MIMI_gp0822 [Acanthamoeba polyphaga mimivirus]	521	100%	0.0	100.00%
176	159	201913	202392	+	hypothetical protein MIMI_gp0821 [Acanthamoeba polyphaga mimivirus]	322	100%	3,00E-111	100.00%
177	490	202446	203918	-	putative ankyrin repeat protein [Acanthamoeba polyphaga mimivirus]	997	100%	0.0	100.00%
178	216	204102	204752	+	hypothetical protein MIMI_gp0819 [Acanthamoeba polyphaga mimivirus]	444	100%	2,00E-157	100.00%
179	74	204784	205008	-	hypothetical protein [Acanthamoeba castellanii mimivirus]	150	100%	1,00E-45	100.00%
180	183	205108	205659	-	putative 5'(3')-deoxyribonucleotidase [Acanthamoeba polyphaga mimivirus]	372	100%	4,00E-130	100.00%
181	396	205794	206984	-	putative F-box protein [Acanthamoeba polyphaga mimivirus]	813	100%	0.0	100.00%
182	180	207178	207720	-	hypothetical protein [Acanthamoeba castellanii mamavirus]	368	100%	1,00E-128	100.00%
183	298	207783	208679	-	putative Fe-S-cluster redox enzyme [Acanthamoeba castellanii mamavirus]	619	100%	0.0	100.00%
184	130	208847	209239	+	hypothetical protein MIMI_gp0815 [Acanthamoeba polyphaga mimivirus]	258	100%	2,00E-86	100.00%
185	113	209205	209543	+	hypothetical protein [Acanthamoeba castellanii mamavirus]	236	100%	3,00E-75	100.00%

186	104	209647	209961	+	hypothetical protein [Acanthamoeba castellanii mamavirus]	194	100%	6,00E-59	100.00%
187	178	210002	210538	+	hypothetical protein [Acanthamoeba castellanii mamavirus]	343	100%	8,00E-119	99.44%
188	86	210616	210876	-	hypothetical protein [Acanthamoeba castellanii mamavirus]	168	100%	2,00E-52	100.00%
189	751	210985	213240	-	hypothetical protein [Acanthamoeba castellanii mamavirus]	1519	100%	0.0	100.00%
190	215	213341	213988	-	hypothetical protein MIMI_gp0811 [Acanthamoeba polyphaga mimivirus]	450	100%	8,00E-160	100.00%
191	149	214099	214548	-	hypothetical protein MIMI_gp0810 [Acanthamoeba polyphaga mimivirus]	293	100%	4,00E-100	100.00%
192	338	215751	216767	+	hypothetical protein MIMI_gp0809 [Acanthamoeba polyphaga mimivirus]	673	100%	0.0	100.00%
193	224	216867	217541	-	putative homeobox protein [Acanthamoeba polyphaga mimivirus]	440	100%	2,00E-155	100.00%
194	1058	217750	220926	-	ankyrin repeat-containing protein [Acanthamoeba castellanii mamavirus]	2130	100%	0.0	100.00%
195	215	221061	221708	-	hypothetical protein MIMI_gp0805 [Acanthamoeba polyphaga mimivirus]	446	100%	5,00E-158	100.00%
196	292	221815	222693	-	hypothetical protein [Acanthamoeba castellanii mamavirus]	582	100%	0.0	100.00%
197	194	222781	223365	+	hypothetical protein [Acanthamoeba castellanii mamavirus]	390	100%	6,00E-137	100.00%
198	449	223478	224827	+	putative BTB/POZ domain-containing protein [Acanthamoeba polyphaga mimivirus]	901	100%	0.0	100.00%
199	81	224901	225146	-	hypothetical protein [Acanthamoeba castellanii mamavirus]	159	100%	6,00E-49	100.00%
200	182	225209	225757	-	hypothetical protein MIMI_gp0800 [Acanthamoeba polyphaga mimivirus]	363	100%	2,00E-126	100.00%
201	187	225876	226439	-	putative mitochondrial protein [Acanthamoeba polyphaga mimivirus]	396	100%	2,00E-139	100.00%
202	490	226485	227957	-	WD40 family protein [Acanthamoeba castellanii mamavirus]	991	100%	0.0	100.00%
203	497	228028	229521	-	WD40 family protein [Acanthamoeba castellanii mamavirus]	1007	100%	0.0	100.00%
204	248	229844	230590	+	hypothetical protein MIMI_gp0796 [Acanthamoeba polyphaga mimivirus]	511	100%	0.0	100.00%
205	276	230673	231503	-	hypothetical protein [Acanthamoeba castellanii mamavirus]	554	100%	0.0	100.00%
206	446	231634	232974	-	hypothetical protein MIMI_gp0794 [Acanthamoeba polyphaga mimivirus]	920	100%	0.0	100.00%
207	652	233194	235152	-	hypothetical protein [Acanthamoeba castellanii mamavirus]	1292	100%	0.0	100.00%
208	524	235349	236923	+	ABC transporter ATP-binding domain protein [Acanthamoeba castellanii mamavirus]	1062	100%	0.0	100.00%
209	188	236976	237542	+	hypothetical protein [Acanthamoeba castellanii mamavirus]	392	100%	1,00E-137	100.00%
210	512	237515	239053	-	putative BTB/POZ domain and WD-repeat protein [Acanthamoeba polyphaga mimivirus]	1017	100%	0.0	100.00%
211	406	239146	240366	-	putative CfxQ-like protein [Acanthamoeba polyphaga mimivirus]	827	100%	0.0	100.00%
212	189	240503	241072	+	hypothetical protein HIRU_S197 [Hirudovirus strain Sangsue]	384	100%	2,00E-134	100.00%
213	567	241174	242877	+	hypothetical protein [Acanthamoeba castellanii mamavirus]	1185	100%	0.0	100.00%
214	284	242929	243783	-	hypothetical protein MIMI_gp0786 [Acanthamoeba polyphaga mimivirus]	554	100%	0.0	100.00%
215	251	243845	244600	-	peptide chain release factor erf1 [Samba virus]	500	100%	2,00E-178	99.60%
216	69	244638	244847	-	peptide chain release factor 1 N [Hirudovirus strain Sangsue]	136	100%	2,00E-40	98.55%
217	224	245141	245815	+	hypothetical protein MIMI_gp0784 [Acanthamoeba polyphaga mimivirus]	456	100%	9,00E-162	100.00%
218	218	245961	246617	+	hypothetical protein [Acanthamoeba castellanii mamavirus]	436	100%	5,00E-154	100.00%
219	221	246694	247359	+	hypothetical protein [Mimivirus reunion]	445	100%	1,00E-157	100.00%
220	872	247450	250068	-	putative bifunctional metalloprotease/ubiquitin-protein ligase [Acanthamoeba polyphaga mimivirus]	1790	100%	0.0	100.00%
221	270	250227	251039	-	putative chemotaxis protein CheD [Acanthamoeba polyphaga mimivirus]	553	100%	0.0	100.00%
222	274	251088	251912	+	endonuclease VIII-like protein [Acanthamoeba polyphaga mimivirus]	568	100%	0.0	100.00%
223	349	251953	253002	+	hypothetical protein lvs_L626 [Acanthamoeba polyphaga lentillevirus]	707	100%	0.0	100.00%
224	130	253030	253422	-	hypothetical protein [Acanthamoeba castellanii mamavirus]	269	100%	6,00E-91	100.00%
225	206	253540	254160	+	hypothetical protein MIMI_gp0776 [Acanthamoeba polyphaga mimivirus]	415	100%	3,00E-146	100.00%
226	205	254231	254848	+	putative glutamine amidotransferase-like protein [Acanthamoeba polyphaga mimivirus]	408	100%	1,00E-143	100.00%
227	275	254929	255756	+	putative ankyrin repeat protein [Acanthamoeba polyphaga mimivirus]	535	100%	0.0	100.00%
228	115	255795	256142	-	hypothetical protein MIMI_gp0773 [Acanthamoeba polyphaga mimivirus]	236	100%	3,00E-78	100.00%
229	214	256243	256887	+	putative membrane protein [Acanthamoeba castellanii mamavirus]	412	100%	5,00E-145	100.00%
230	976	257077	260007	-	hypothetical protein [Acanthamoeba castellanii mamavirus]	1904	100%	0.0	100.00%
231	530	260143	261735	+	hypothetical protein MIMI_gp0770 [Acanthamoeba polyphaga mimivirus]	1094	100%	0.0	100.00%
232	328	261857	262843	+	hypothetical protein [Acanthamoeba castellanii mamavirus]	661	100%	0.0	100.00%
233	194	262923	263507	-	hypothetical protein [Acanthamoeba castellanii mamavirus]	408	100%	7,00E-144	100.00%
234	388	263650	264816	+	probable ubiquitin-conjugating enzyme E2 [Acanthamoeba polyphaga mimivirus]	770	100%	0.0	100.00%
235	204	264942	265556	-	uncharacterized phosphoglycerate mutase family protein [Acanthamoeba polyphaga mimivirus]	419	100%	8,00E-148	100.00%
236	281	265627	266472	-	putative glycosyltransferase [Acanthamoeba castellanii mamavirus]	576	100%	0.0	100.00%
237	420	266518	267780	-	hypothetical protein [Acanthamoeba castellanii mamavirus]	845	100%	0.0	100.00%
238	227	267933	268616	-	hypothetical protein HIRU_S223 [Hirudovirus strain Sangsue]	420	100%	1,00E-147	98.68%
239	565	269108	270805	-	hypothetical protein [Acanthamoeba castellanii mamavirus]	1150	100%	0.0	100.00%
240	135	270850	271257	-	hypothetical protein MIMI_gp0761 [Acanthamoeba polyphaga mimivirus]	276	100%	2,00E-93	100.00%
241	764	271387	273681	+	putative protein kinase [Acanthamoeba castellanii mamavirus]	1540	100%	0.0	100.00%
242	203	273730	274341	+	hypothetical protein [Acanthamoeba castellanii mamavirus]	417	100%	4,00E-147	100.00%
243	401	274349	275554	-	Serpin [Acanthamoeba castellanii mamavirus]	816	100%	0.0	100.00%
244	455	275628	276995	-	putative procollagen-lysine,2-oxoglutarate dioxygenase [Acanthamoeba polyphaga mimivirus]	941	100%	0.0	100.00%
245	300	277218	278120	+	hypothetical protein [Acanthamoeba castellanii mamavirus]	607	100%	0.0	100.00%
246	375	278241	279368	+	hypothetical protein [Acanthamoeba castellanii mamavirus]	748	100%	0.0	100.00%
247	63	279424	279615	+	hypothetical protein MIMI_gp0754 [Acanthamoeba polyphaga mimivirus]	128	100%	3,00E-37	100.00%
248	313	279692	280633	+	hypothetical protein [Acanthamoeba castellanii mamavirus]	632	100%	0.0	100.00%
249	144	280646	281080	-	hypothetical protein [Acanthamoeba castellanii mamavirus]	291	100%	2,00E-99	100.00%
250	127	281146	281529	-	putative potassium channel protein [Hirudovirus strain Sangsue]	250	100%	1,00E-83	98.43%
251	149	281507	281956	-	probable methylated-DNA--protein-cysteine methyltransferase [Acanthamoeba polyphaga mimivirus]	307	100%	2,00E-105	100.00%
252	354	282029	283093	-	hypothetical protein MIMI_gp0749 [Acanthamoeba polyphaga mimivirus]	722	100%	0.0	100.00%
253	275	283164	283991	-	hypothetical protein [Acanthamoeba castellanii mamavirus]	560	100%	0.0	100.00%
254	374	284156	285280	+	hypothetical protein MIMI_gp0746 [Acanthamoeba polyphaga mimivirus]	761	100%	0.0	100.00%
255	255	285346	286113	-	probable UDP-N-acetylglucosamine pyrophosphorylase [Acanthamoeba polyphaga mimivirus]	508	100%	0.0	100.00%
256	236	286210	286920	+	hypothetical protein MIMI_gp0744 [Acanthamoeba polyphaga mimivirus]	464	100%	8,00E-165	100.00%
257	330	286979	287971	+	putative UV damage endonuclease [Acanthamoeba castellanii mamavirus]	681	100%	0.0	100.00%
258	219	288054	288713	+	hypothetical protein [Acanthamoeba castellanii mamavirus]	432	100%	1,00E-152	100.00%
259	149	289680	290129	-	hypothetical protein HIRU_S244 [Hirudovirus strain Sangsue]	289	100%	3,00E-98	99.33%
260	333	290405	291406	+	hypothetical protein [Acanthamoeba castellanii mamavirus]	659	100%	0.0	100.00%
261	192	291505	292083	+	hypothetical protein [Acanthamoeba castellanii mamavirus]	384	100%	1,00E-134	100.00%
262	498	292061	293557	+	Endonuclease/Exonuclease/phosphatase family protein [Acanthamoeba castellanii mamavirus]	991	100%	0.0	100.00%
263	222	293635	294303	+	hypothetical protein [Acanthamoeba castellanii mamavirus]	457	100%	2,00E-162	100.00%
264	114	294381	294725	+	hypothetical protein MIMI_gp0736 [Acanthamoeba polyphaga mimivirus]	230	100%	5,00E-76	100.00%
265	151	295002	295457	-	hypothetical protein MIMI_gp0734 [Acanthamoeba polyphaga mimivirus]	302	100%	2,00E-103	100.00%
266	111	295521	295856	-	hypothetical protein MIMI_gp0733 [Acanthamoeba polyphaga mimivirus]	215	100%	3,00E-70	100.00%
267	694	295961	298045	-	putative serine/threonine-protein kinase [Acanthamoeba polyphaga mimivirus]	1407	100%	0.0	100.00%
268	255	298176	298943	+	putative SET domain-containing protein [Acanthamoeba polyphaga mimivirus]	521	100%	0.0	100.00%
269	93	299071	299352	+	hypothetical protein [Samba virus]	180	100%	7,00E-57	100.00%
270	60	299396	299578	-	hypothetical protein MIMI_gp0729 [Acanthamoeba polyphaga mimivirus]	117	100%	6,00E-33	100.00%
271	165	299672	300169	+	hypothetical protein MIMI_gp0728 [Acanthamoeba polyphaga mimivirus]	330	100%	4,00E-114	100.00%
272	472	300203	301621	+	ankyrin repeat-containing protein [Acanthamoeba castellanii mamavirus]	967	100%	0.0	100.00%
273	488	301689	303155	+	hypothetical protein [Acanthamoeba castellanii mamavirus]	979	100%	0.0	100.00%
274	60	303476	303658	+	orphan [Mimivirus reunion]	124	100%	9,00E-36	100.00%
275	66	303803	304003	+	hypothetical protein MIMI_gp0725 [Acanthamoeba polyphaga mimivirus]	125	100%	5,00E-36	100.00%
276	96	304049	304339	+	hypothetical protein [Acanthamoeba polyphaga mimivirus]	188	100%	4,00E-60	100.00%
277	547	304472	306115	+	putative serine/threonine-protein kinase [Acanthamoeba polyphaga mimivirus]	1106	100%	0.0	99.27%
278	111	306189	306524	+	hypothetical protein MIMI_gp0723 [Acanthamoeba polyphaga mimivirus]	237	100%	5,00E-79	100.00%
279	152	306646	307104	+	hypothetical protein MIMI_gp0722 [Acanthamoeba polyphaga mimivirus]	307	100%	2,00E-105	100.00%

280	539	307200	308819	+	serine-threonine kinase [Acanthamoeba castellanii mamavirus]	1092	100%	0.0	100.00%
281	1916	308928	314678	+	Collagen triple helix repeat containing protein [Acanthamoeba castellanii mamavirus]	3575	100%	0.0	100.00%
282	1312	314810	318748	+	collagen triple helix repeat containing protein [Mimivirus Bombay]	2467	100%	0.0	98.78%
283	148	318810	319256	-	hypothetical protein [Acanthamoeba castellanii mamavirus]	302	100%	1,00E-103	100.00%
284	59	319331	319510	-	orphan [Mimivirus reunion]	120	100%	2,00E-34	100.00%
285	245	319603	320340	+	putative ATPase [Acanthamoeba castellanii mamavirus]	492	100%	1,00E-175	100.00%
286	467	320417	321820	-	putative oxidoreductase [Acanthamoeba polyphaga mimivirus]	978	100%	0.0	100.00%
287	162	321928	322416	-	putative ankyrin repeat protein [Acanthamoeba polyphaga mimivirus]	321	100%	2,00E-110	100.00%
288	600	322536	324338	-	Arginyl-tRNA synthetase [Acanthamoeba castellanii mamavirus]	1211	100%	0.0	100.00%
289	462	324487	325875	+	hypothetical protein [Acanthamoeba castellanii mamavirus]	951	100%	0.0	100.00%
290	73	325943	326164	-	hypothetical protein [Acanthamoeba polyphaga mimivirus]	136	100%	5,00E-40	100.00%
291	211	326186	326821	-	hypothetical protein [Acanthamoeba castellanii mamavirus]	421	100%	3,00E-148	100.00%
292	206	327097	327717	-	hypothetical protein [Acanthamoeba castellanii mamavirus]	419	100%	9,00E-148	100.00%
293	508	327861	329387	-	hypothetical protein [Acanthamoeba castellanii mamavirus]	1048	100%	0.0	100.00%
294	541	329536	331161	-	hypothetical protein [Acanthamoeba castellanii mamavirus]	1108	100%	0.0	100.00%
295	204	331202	331816	+	hypothetical protein [Acanthamoeba castellanii mamavirus]	403	100%	1,00E-141	100.00%
296	482	331906	333354	-	FAD dependent oxidoreductase family protein [Acanthamoeba castellanii mamavirus]	993	100%	0.0	100.00%
297	324	333573	334547	-	putative glycosyltransferase [Acanthamoeba polyphaga mimivirus]	667	100%	0.0	100.00%
298	600	334653	336455	-	glycosyltransferase family 10 [Acanthamoeba castellanii mamavirus]	1224	100%	0.0	100.00%
299	227	336526	337209	-	hypothetical protein [Acanthamoeba castellanii mamavirus]	471	100%	8,00E-168	100.00%
300	352	337243	338301	-	hypothetical protein [Acanthamoeba castellanii mamavirus]	706	100%	0.0	100.00%
301	493	338397	339878	-	hypothetical protein [Acanthamoeba castellanii mamavirus]	1009	100%	0.0	100.00%
302	422	340003	341271	-	hypothetical protein [Acanthamoeba castellanii mamavirus]	850	100%	0.0	100.00%
303	77	341330	341563	-	hypothetical protein MIMI_gp0699 [Acanthamoeba polyphaga mimivirus]	157	100%	3,00E-48	100.00%
304	281	341720	342565	+	hypothetical protein [Acanthamoeba castellanii mamavirus]	559	100%	0.0	100.00%
305	400	342647	343849	-	NHL repeat-containing protein [Acanthamoeba castellanii mamavirus]	815	100%	0.0	100.00%
306	188	344099	344665	+	hypothetical protein [Acanthamoeba polyphaga mimivirus]	357	100%	5,00E-124	98.94%
307	273	344746	345567	-	hypothetical protein MIMI_gp0695 [Acanthamoeba polyphaga mimivirus]	540	100%	0.0	100.00%
308	407	345682	346905	+	low complexity hypothetical protein [Acanthamoeba castellanii mamavirus]	797	100%	0.0	100.00%
309	143	346902	347333	-	phosphatidylethanolamine-binding protein [Acanthamoeba castellanii mamavirus]	292	100%	1,00E-99	100.00%
310	359	347417	348496	-	hypothetical protein [Acanthamoeba castellanii mamavirus]	683	100%	0.0	100.00%
311	526	348707	350287	-	hypothetical protein [Acanthamoeba castellanii mamavirus]	1070	100%	0.0	100.00%
312	481	350414	351859	-	hypothetical protein [Acanthamoeba castellanii mamavirus]	984	100%	0.0	100.00%
313	550	352222	353874	-	Methlonyl-tRNA synthetase [Acanthamoeba castellanii mamavirus]	1138	100%	0.0	100.00%
314	134	353884	354288	-	hypothetical protein [Acanthamoeba castellanii mamavirus]	270	100%	4,00E-91	100.00%
315	153	354353	354814	-	hypothetical protein [Acanthamoeba castellanii mamavirus]	311	100%	9,00E-107	100.00%
316	152	354955	355413	+	hypothetical protein [Acanthamoeba castellanii mamavirus]	315	100%	3,00E-108	100.00%
317	394	355394	356578	-	FNIP repeat-containing protein [Acanthamoeba castellanii mamavirus]	798	100%	0.0	100.00%
318	70	356638	356850	-	hypothetical protein MIMI_gp0684 [Acanthamoeba polyphaga mimivirus]	142	100%	1,00E-42	100.00%
319	408	356889	358115	-	hypothetical protein [Acanthamoeba castellanii mamavirus]	844	100%	0.0	100.00%
320	486	358217	359677	-	ankyrin repeat-containing protein [Acanthamoeba castellanii mamavirus]	982	100%	0.0	100.00%
321	58	359847	360023	-	No significant similarity found	-	-	-	-
322	172	360466	360984	+	Bulb-type mannose-specific lectin [Acanthamoeba castellanii mamavirus]	347	100%	5,00E-120	100.00%
323	51	361047	361202	-	orphan [Mimivirus reunion]	87.8	100%	2,00E-21	100.00%
324	321	361199	362164	-	putative N-acylsphingosine amidohydrolase [Acanthamoeba castellanii mamavirus]	656	100%	0.0	100.00%
325	227	362237	362920	+	hypothetical protein [Acanthamoeba castellanii mamavirus]	458	100%	1,00E-162	100.00%
326	200	362974	363576	+	uncharacterized transmembrane protein [Acanthamoeba polyphaga mimivirus]	408	100%	1,00E-143	100.00%
327	322	363646	364614	+	hypothetical protein MIMI_gp0676 [Acanthamoeba polyphaga mimivirus]	650	100%	0.0	100.00%
328	89	364766	365035	+	cyt-b5 [Acanthamoeba polyphaga mimivirus]	181	100%	2,00E-57	100.00%
329	643	365139	367070	-	hypothetical protein [Acanthamoeba castellanii mamavirus]	1256	100%	0.0	100.00%
330	599	367161	368960	-	hypothetical protein [Acanthamoeba castellanii mamavirus]	1228	100%	0.0	100.00%
331	166	369265	369765	-	GTP-binding protein-like protein [Acanthamoeba castellanii mamavirus]	337	100%	2,00E-116	100.00%
332	479	369850	371289	-	GTP binding elongation factor eF-Tu [Acanthamoeba castellanii mamavirus]	969	100%	0.0	100.00%
333	269	372066	372875	-	hypothetical protein [Acanthamoeba castellanii mamavirus]	543	100%	0.0	100.00%
334	212	373020	373658	-	putative tyrosine-protein phosphatase [Acanthamoeba polyphaga mimivirus]	439	100%	8,00E-156	100.00%
335	358	373807	374883	+	putative N-myristoyltransferase [Hirudovirus strain Sangsue]	724	100%	0.0	100.00%
336	368	375004	376110	+	Patatin phospholipase [Acanthamoeba castellanii mamavirus]	748	100%	0.0	100.00%
337	606	376146	377966	+	putative glucosamine-fructose-6-phosphate aminotransferase [Acanthamoeba castellanii mamavirus]	1254	100%	0.0	100.00%
338	592	377986	379764	-	putative GTPase [Acanthamoeba castellanii mamavirus]	1188	100%	0.0	100.00%
339	170	379846	380358	-	hypothetical protein MIMI_gp0663 [Acanthamoeba polyphaga mimivirus]	357	100%	1,00E-124	100.00%
340	353	380532	381593	-	hypothetical protein MIMI_gp0662 [Acanthamoeba polyphaga mimivirus]	728	100%	0.0	100.00%
341	701	381796	383901	+	Phosphoinositide 3-kinase [Acanthamoeba castellanii mamavirus]	1427	100%	0.0	100.00%
342	303	384097	385008	-	Stomatin family protein [Acanthamoeba castellanii mamavirus]	616	100%	0.0	100.00%
343	386	385134	386294	+	hypothetical protein [Acanthamoeba castellanii mamavirus]	788	100%	0.0	100.00%
344	160	386365	386847	+	Mannose-6P isomerase [Acanthamoeba castellanii mamavirus]	332	100%	4,00E-115	100.00%
345	953	387009	389870	+	P-loop ATPase/GTPase-containing domain protein [Acanthamoeba castellanii mamavirus]	1964	100%	0.0	100.00%
346	316	389947	390897	-	Proline rich protein [Acanthamoeba castellanii mamavirus]	624	100%	0.0	100.00%
347	318	391175	392131	+	hypothetical protein [Acanthamoeba castellanii mamavirus]	573	100%	0.0	93.71%
348	246	392133	392873	-	hypothetical protein [Acanthamoeba castellanii mamavirus]	495	100%	1,00E-176	100.00%
349	298	392927	393823	-	hypothetical protein [Acanthamoeba castellanii mamavirus]	605	100%	0.0	100.00%
350	170	394011	394523	+	hypothetical protein [Acanthamoeba castellanii mamavirus]	343	100%	3,00E-119	100.00%
351	234	394572	395276	+	Cyclophilin family protein [Acanthamoeba castellanii mamavirus]	478	100%	2,00E-170	100.00%
352	95	395341	395628	-	hypothetical protein [Acanthamoeba castellanii mamavirus]	196	100%	3,00E-63	100.00%
353	375	395715	396842	-	hypothetical protein [Acanthamoeba castellanii mamavirus]	760	100%	0.0	100.00%
354	309	396939	397868	-	ankyrin repeat-containing protein [Acanthamoeba castellanii mamavirus]	616	100%	0.0	100.00%
355	304	397970	398884	-	ankyrin repeat-containing protein [Acanthamoeba castellanii mamavirus]	614	100%	0.0	100.00%
356	303	398937	399848	-	ankyrin repeat-containing protein [Acanthamoeba castellanii mamavirus]	610	100%	0.0	100.00%
357	315	399940	400887	-	ankyrin repeat-containing protein [Acanthamoeba castellanii mamavirus]	630	100%	0.0	100.00%
358	358	400955	402031	-	ankyrin repeat-containing protein [Acanthamoeba castellanii mamavirus]	696	100%	0.0	99.72%
359	304	402025	402939	-	ankyrin repeat-containing protein [Acanthamoeba castellanii mamavirus]	601	100%	0.0	100.00%
360	276	402998	403828	-	hypothetical protein [Acanthamoeba castellanii mamavirus]	546	100%	0.0	100.00%
361	292	403888	404766	-	Thiol oxidoreductase E10R [Acanthamoeba castellanii mamavirus]	612	100%	0.0	100.00%
362	70	404884	405096	-	hypothetical protein MIMI_gp0639 [Acanthamoeba polyphaga mimivirus]	147	100%	1,00E-44	100.00%
363	294	405210	406094	-	putative phospholipase/carboxylesterase family protein [Hirudovirus strain Sangsue]	594	100%	0.0	100.00%
364	390	406246	407418	+	hypothetical protein MIMI_L594 [Acanthamoeba polyphaga mimivirus]	797	100%	0.0	100.00%
365	242	407470	408198	+	putative prolyl 4-hydroxylase [Acanthamoeba polyphaga mimivirus]	503	100%	8,00E-180	100.00%
366	841	408301	410826	-	RING-finger-containing E3 ubiquitin ligase [Acanthamoeba castellanii mamavirus]	1712	100%	0.0	100.00%
367	259	410955	411734	+	hypothetical protein MIMI_gp0634 [Acanthamoeba polyphaga mimivirus]	531	100%	0.0	100.00%
368	190	411850	412422	+	hypothetical protein MIMI_L590 [Acanthamoeba polyphaga mimivirus]	395	100%	7,00E-139	100.00%
369	272	412554	413372	+	ankyrin repeat-containing protein [Acanthamoeba castellanii mamavirus]	555	100%	0.0	100.00%
370	397	413459	414652	-	hypothetical protein [Acanthamoeba castellanii mamavirus]	825	100%	0.0	100.00%
371	185	414792	415349	-	hypothetical protein [Acanthamoeba castellanii mamavirus]	389	100%	1,00E-136	100.00%
372	103	415495	415806	+	hypothetical protein MIMI_gp0629 [Acanthamoeba polyphaga mimivirus]	205	100%	2,00E-66	100.00%
373	107	415877	416200	+	hypothetical protein [Acanthamoeba castellanii mamavirus]	210	100%	3,00E-68	100.00%

374	411	416248	417483	-	hypothetical protein [Acanthamoeba castellanii mamavirus]	848	100%	0.0	100.00%
375	104	417581	417895	+	hypothetical protein MIMI_gp0626 [Acanthamoeba polyphaga mimivirus]	207	100%	3,00E-67	100.00%
376	179	417918	418457	+	hypothetical protein MIMI_gp0625 [Acanthamoeba polyphaga mimivirus]	350	100%	3,00E-121	100.00%
377	741	418724	420949	+	ATP-dependent helicase HrpA [Acanthamoeba castellanii mamavirus]	1517	100%	0.0	100.00%
378	470	421003	422415	-	ankyrin repeat-containing protein [Acanthamoeba castellanii mamavirus]	947	100%	0.0	100.00%
379	463	422516	423907	-	hypothetical protein [Acanthamoeba castellanii mamavirus]	932	100%	0.0	100.00%
380	419	424005	425264	-	ankyrin repeat-containing protein [Acanthamoeba castellanii mamavirus]	847	100%	0.0	100.00%
381	104	425430	425744	+	hypothetical protein [Acanthamoeba castellanii mamavirus]	208	100%	1,00E-67	100.00%
382	296	425811	426701	+	hypothetical protein [Acanthamoeba castellanii mamavirus]	595	100%	0.0	100.00%
383	285	426746	427603	+	hypothetical protein [Acanthamoeba castellanii mamavirus]	570	100%	0.0	100.00%
384	273	427655	428476	+	hypothetical protein [Acanthamoeba castellanii mamavirus]	547	100%	0.0	100.00%
385	281	428514	429359	+	hypothetical protein [Acanthamoeba castellanii mamavirus]	562	100%	0.0	100.00%
386	485	429449	430906	+	AAA family ATPase [Acanthamoeba castellanii mamavirus]	988	100%	0.0	100.00%
387	297	430951	431844	-	Pat_ExoU_VipD-like protein [Acanthamoeba castellanii mamavirus]	603	100%	0.0	100.00%
388	247	431902	432645	-	hypothetical protein [Acanthamoeba castellanii mamavirus]	493	100%	9,00E-176	100.00%
389	1031	432770	435865	-	UvrD/REP helicase family protein [Acanthamoeba castellanii mamavirus]	2103	100%	0.0	100.00%
390	320	435982	436944	+	hypothetical protein [Acanthamoeba castellanii mamavirus]	671	100%	0.0	100.00%
391	52	437066	437224	-	hypothetical protein [Acanthamoeba polyphaga mimivirus]	67.4	69%	2,00E-11	91.67%
392	88	437199	437465	-	hypothetical protein MIMI_gp0610 [Acanthamoeba polyphaga mimivirus]	176	98%	5,00E-54	97.70%
393	126	437576	437956	-	hypothetical protein [Acanthamoeba castellanii mamavirus]	254	100%	2,00E-85	100.00%
394	75	437937	438164	-	hypothetical protein [Acanthamoeba polyphaga mimivirus]	136	96%	8,00E-36	91.67%
395	144	438594	439028	+	hypothetical protein [Acanthamoeba castellanii mamavirus]	299	100%	2,00E-102	100.00%
396	353	439124	440185	-	glutamine synthetase [Acanthamoeba castellanii mamavirus]	739	100%	0.0	100.00%
397	137	440255	440668	-	hypothetical protein [Acanthamoeba castellanii mamavirus]	279	100%	1,00E-94	100.00%
398	573	440721	442442	-	putative ATP-dependent RNA helicase [Acanthamoeba polyphaga mimivirus]	1181	100%	0.0	100.00%
399	321	442554	443519	-	hypothetical protein MIMI_gp0605 [Acanthamoeba polyphaga mimivirus]	659	100%	0.0	100.00%
400	256	443772	444542	-	hypothetical protein MIMI_gp0604 [Acanthamoeba polyphaga mimivirus]	511	100%	0.0	100.00%
401	187	444671	445234	+	Numod4 NHN endonuclease [Acanthamoeba castellanii mamavirus]	384	100%	1,00E-134	100.00%
402	226	445390	446070	-	Helix-turn-helix XRE-family like protein [Acanthamoeba castellanii mamavirus]	459	100%	3,00E-163	100.00%
403	68	446134	446340	-	putative membrane protein [Acanthamoeba castellanii mamavirus]	128	100%	3,00E-37	100.00%
404	465	446404	447801	-	hypothetical protein [Acanthamoeba castellanii mamavirus]	911	100%	0.0	100.00%
405	160	447876	448358	-	hypothetical protein [Acanthamoeba castellanii mamavirus]	329	100%	1,00E-113	100.00%
406	277	448502	449335	-	hypothetical protein [Acanthamoeba castellanii mamavirus]	561	100%	0.0	100.00%
407	53	449310	449471	-	No significant similarity found	-	-	-	-
408	1351	449461	453516	-	putative DNA repair protein [Acanthamoeba castellanii mamavirus]	2723	100%	0.0	100.00%
409	402	453659	454867	-	hypothetical protein [Acanthamoeba castellanii mamavirus]	801	100%	0.0	100.00%
410	518	454964	456520	-	hypothetical protein [Acanthamoeba castellanii mamavirus]	1061	100%	0.0	100.00%
411	308	456560	457486	+	hypothetical protein HIRU_S392 [Hirudovirus strain Sangsue]	620	100%	0.0	100.00%
412	285	457545	458402	-	hypothetical protein [Acanthamoeba castellanii mamavirus]	573	100%	0.0	100.00%
413	167	458629	459132	+	hypothetical protein MIMI_gp0592 [Acanthamoeba polyphaga mimivirus]	301	100%	1,00E-102	100.00%
414	335	459739	460746	-	hypothetical protein lvs_R476 [Acanthamoeba polyphaga lentillevirus]	668	100%	0.0	100.00%
415	141	460823	461248	-	Thioredoxin [Acanthamoeba castellanii mamavirus]	286	100%	1,00E-97	100.00%
416	122	461408	461776	-	hypothetical protein lvs_R474 [Acanthamoeba polyphaga lentillevirus]	253	100%	6,00E-85	100.00%
417	213	461939	462580	-	MAMA_R546 [Acanthamoeba castellanii mamavirus]	406	100%	1,00E-142	100.00%
418	282	462675	463523	-	hypothetical protein [Acanthamoeba castellanii mamavirus]	510	100%	0.0	100.00%
419	385	463668	464825	+	hypothetical protein [Acanthamoeba castellanii mamavirus]	766	100%	0.0	99.48%
420	330	464863	465855	+	ADP-ribosyltransferase [Acanthamoeba castellanii mamavirus]	688	100%	0.0	100.00%
421	547	465926	467569	-	hypothetical protein lvs_R470 [Acanthamoeba polyphaga lentillevirus]	1095	100%	0.0	100.00%
422	306	467637	468557	-	BTB family protein [Acanthamoeba castellanii mamavirus]	606	100%	0.0	99.67%
423	159	468536	469015	-	C-terminal divergent BTB domain-containing protein [Acanthamoeba castellanii mamavirus]	321	100%	1,00E-110	100.00%
424	695	469153	471240	+	VVI8 helicase [Acanthamoeba polyphaga lentillevirus]	1432	100%	0.0	100.00%
425	298	471259	472155	+	hypothetical protein [Acanthamoeba castellanii mamavirus]	594	100%	0.0	99.66%
426	74	472211	472435	-	putative membrane protein [Acanthamoeba castellanii mamavirus]	150	100%	1,00E-45	100.00%
427	60	472509	472691	-	hypothetical protein MIMI_gp0579 [Acanthamoeba polyphaga mimivirus]	114	100%	8,00E-32	100.00%
428	445	472780	474117	+	putative ATP-dependent RNA helicase [Acanthamoeba polyphaga mimivirus]	900	100%	0.0	100.00%
429	473	474224	475645	+	hypothetical protein [Acanthamoeba castellanii mamavirus]	959	100%	0.0	100.00%
430	448	475739	476248	+	hypothetical protein [Acanthamoeba castellanii mamavirus]	342	100%	1,00E-118	100.00%
431	507	476225	477748	-	hypothetical protein [Acanthamoeba castellanii mamavirus]	998	100%	0.0	100.00%
432	307	477916	478839	+	nudix hydrolase [Acanthamoeba castellanii mamavirus]	623	100%	0.0	100.00%
433	508	478960	480486	+	hypothetical protein [Acanthamoeba castellanii mamavirus]	1038	100%	0.0	100.00%
434	468	480581	481987	+	Cytochrome P450 [Acanthamoeba castellanii mamavirus]	947	100%	0.0	100.00%
435	294	482009	482893	+	hypothetical protein [Acanthamoeba castellanii mamavirus]	609	100%	0.0	100.00%
436	494	482978	484462	-	putative DNA helicase [Acanthamoeba polyphaga mimivirus]	1002	100%	0.0	100.00%
437	164	484606	485100	+	hypothetical protein MIMI_gp0569 [Acanthamoeba polyphaga mimivirus]	333	100%	2,00E-115	100.00%
438	785	485169	487526	-	XRN 5'-3' exonuclease [Acanthamoeba castellanii mamavirus]	1593	100%	0.0	100.00%
439	342	487592	488620	-	putative triacylglycerol lipase [Acanthamoeba castellanii mamavirus]	704	100%	0.0	100.00%
440	557	488696	490369	-	hypothetical protein [Acanthamoeba castellanii mamavirus]	1109	100%	0.0	100.00%
441	379	490556	491695	+	putative divergent NUDIX hydrolase [Acanthamoeba castellanii mamavirus]	771	100%	0.0	100.00%
442	118	491946	492302	+	Integral membrane protein [Acanthamoeba castellanii mamavirus]	235	100%	3,00E-77	100.00%
443	264	492292	493086	-	hypothetical protein [Acanthamoeba castellanii mamavirus]	520	100%	0.0	100.00%
444	869	493187	495796	-	ubiquitin-conjugating enzyme E2 [Acanthamoeba castellanii mamavirus]	1798	100%	0.0	100.00%
445	546	495930	497570	-	hypothetical protein lvs_R450 [Acanthamoeba polyphaga lentillevirus]	1118	99%	0.0	100.00%
446	828	497699	500185	+	BTB family protein [Acanthamoeba castellanii mamavirus]	1648	100%	0.0	100.00%
447	615	500214	502061	-	putative peptidase [Acanthamoeba castellanii mamavirus]	1243	100%	0.0	100.00%
448	453	502253	503614	-	Serine/Threonine protein kinase [Acanthamoeba castellanii mamavirus]	919	100%	0.0	100.00%
449	221	503706	504371	+	hypothetical protein MIMI_gp0557 [Acanthamoeba polyphaga mimivirus]	432	100%	1,00E-152	100.00%
450	650	504414	506366	+	hypothetical protein [Acanthamoeba castellanii mamavirus]	1282	100%	0.0	100.00%
451	729	506448	508637	+	hypothetical protein [Acanthamoeba castellanii mamavirus]	1443	100%	0.0	100.00%
452	209	508640	509269	-	hypothetical protein MIMI_gp0554 [Acanthamoeba polyphaga mimivirus]	397	100%	3,00E-139	100.00%
453	93	509341	509622	-	hypothetical protein MIMI_gp0553 [Acanthamoeba polyphaga mimivirus]	192	100%	1,00E-61	100.00%
454	168	509754	510260	-	hypothetical protein MIMI_gp0552 [Acanthamoeba polyphaga mimivirus]	332	100%	8,00E-115	100.00%
455	193	510356	510937	-	Deoxynucleotide monophosphate kinase [Acanthamoeba castellanii mamavirus]	405	100%	9,00E-143	100.00%
456	788	511077	513443	+	ftsJ methyltransferase domain-containing protein 1 [Acanthamoeba castellanii mamavirus]	1599	100%	0.0	100.00%
457	363	513487	514578	-	putative replication factor C small subunit [Acanthamoeba polyphaga mimivirus]	736	100%	0.0	100.00%
458	280	514729	515571	+	putative metallo-dependent phosphatase [Acanthamoeba polyphaga mimivirus]	560	100%	0.0	100.00%
459	335	515640	516647	-	putative SWIB domain-containing protein [Acanthamoeba polyphaga mimivirus]	647	100%	0.0	100.00%
460	259	516754	517533	+	hypothetical protein MIMI_gp0546 [Acanthamoeba polyphaga mimivirus]	520	100%	0.0	100.00%
461	320	517597	518559	-	hypothetical protein MIMI_gp0545 [Acanthamoeba polyphaga mimivirus]	650	100%	0.0	100.00%
462	405	518745	519962	-	hypothetical protein [Acanthamoeba castellanii mamavirus]	811	100%	0.0	100.00%
463	406	520185	521405	+	hypothetical protein [Acanthamoeba castellanii mamavirus]	811	100%	0.0	100.00%
464	232	521523	522221	-	hypothetical protein [Acanthamoeba castellanii mamavirus]	465	100%	3,00E-165	100.00%
465	967	522334	525237	-	DNA-directed RNA polymerase subunit 1 [Acanthamoeba polyphaga mimivirus]	1982	100%	0.0	99.90%
466	256	525569	526339	-	Group I intron endonuclease [Acanthamoeba castellanii mamavirus]	532	100%	0.0	100.00%
467	514	526660	528204	-	DNA directed RNA polymerase (II) subunit 1 [Acanthamoeba castellanii mamavirus]	811	100%	0.0	100.00%

468	83	528337	528588	+	hypothetical protein HIRU_S447 [Hirudovirus strain Sangsue]	166	100%	1,00E-51	100.00%
469	183	528585	529136	-	hypothetical protein [Acanthamoeba polyphaga mimivirus]	373	100%	2,00E-130	100.00%
470	110	529238	529570	-	hypothetical protein [Acanthamoeba castellanii mamavirus]	215	100%	2,00E-70	100.00%
471	344	529781	530815	+	putative replication factor C small subunit [Acanthamoeba polyphaga mimivirus]	700	100%	0.0	100.00%
472	422	530914	532182	+	probable zinc-type alcohol dehydrogenase-like protein [Acanthamoeba polyphaga mimivirus]	875	100%	0.0	100.00%
473	563	532249	533940	-	bifunctional dihydrofolate reductase-thymidylate synthase [Acanthamoeba polyphaga mimivirus]	1169	100%	0.0	100.00%
474	264	534100	534894	+	Translation initiation factor 4E [Acanthamoeba castellanii mamavirus]	540	100%	0.0	100.00%
475	140	534910	535332	-	hypothetical protein [Acanthamoeba castellanii mamavirus]	279	100%	9,00E-95	100.00%
476	273	535425	536246	+	hypothetical protein [Acanthamoeba castellanii mamavirus]	550	100%	0.0	100.00%
477	464	536297	537691	-	probable DNA polymerase sliding clamp [Acanthamoeba polyphaga mimivirus]	928	100%	0.0	99.78%
478	179	537852	538391	+	hypothetical protein MIMI_gp0531 [Acanthamoeba polyphaga mimivirus]	368	100%	1,00E-128	100.00%
479	247	538437	539180	+	hypothetical protein [Acanthamoeba castellanii mamavirus]	492	100%	2,00E-175	100.00%
480	511	539260	540795	+	hypothetical protein MIMI_gp0529 [Acanthamoeba polyphaga mimivirus]	1020	100%	0.0	100.00%
481	82	540872	541120	+	hypothetical protein MIMI_gp0528 [Acanthamoeba polyphaga mimivirus]	164	100%	5,00E-51	100.00%
482	221	541195	541860	-	hypothetical protein MIMI_gp0527 [Acanthamoeba polyphaga mimivirus]	456	100%	7,00E-162	100.00%
483	559	542094	543773	+	hypothetical protein [Acanthamoeba castellanii mamavirus]	1135	100%	0.0	100.00%
484	93	543919	544200	+	hypothetical protein MIMI_gp0525 [Acanthamoeba polyphaga mimivirus]	182	100%	7,00E-58	100.00%
485	330	544298	545290	+	hypothetical protein [Acanthamoeba castellanii mamavirus]	650	100%	0.0	100.00%
486	223	545342	546013	-	putative PAN domain-containing protein [Acanthamoeba polyphaga mimivirus]	458	100%	7,00E-163	100.00%
487	170	546434	546946	+	hypothetical protein MIMI_gp0521 [Acanthamoeba polyphaga mimivirus]	350	100%	6,00E-122	100.00%
488	1908	547116	552842	+	ankyrin repeat-containing protein [Acanthamoeba castellanii mamavirus]	3835	100%	0.0	100.00%
489	408	552972	554198	+	putative ankyrin repeat protein [Acanthamoeba polyphaga mimivirus]	816	100%	0.0	100.00%
490	82	554249	554497	+	hypothetical protein MIMI_gp0518 [Acanthamoeba polyphaga mimivirus]	156	100%	1,00E-47	100.00%
491	100	554899	555201	+	ankyrin repeat-containing protein [Acanthamoeba castellanii mamavirus]	201	100%	8,00E-65	99.00%
492	902	555261	557969	-	hypothetical protein [Acanthamoeba castellanii mamavirus]	1780	100%	0.0	100.00%
493	1263	558062	561853	-	Topoisomerase II [Acanthamoeba castellanii mamavirus]	2583	100%	0.0	100.00%
494	251	562073	562828	+	Phosphoribosyl-ATP pyrophosphohydrolase-domain containing protein (two domains) [Acanthamoeba castellanii mamavirus]	513	100%	0.0	100.00%
495	370	562959	564071	+	putative replication factor C small subunit [Acanthamoeba polyphaga mimivirus]	751	100%	0.0	100.00%
496	311	564216	565151	+	Cathepsin B, cystein protease [Acanthamoeba castellanii mamavirus]	644	100%	0.0	100.00%
497	91	565216	565491	-	hypothetical protein MIMI_gp0511 [Acanthamoeba polyphaga mimivirus]	170	100%	7,00E-53	100.00%
498	855	565712	568279	-	AAA family ATPase [Acanthamoeba castellanii mamavirus]	1730	100%	0.0	100.00%
499	550	568402	570054	-	asparagine synthase [Acanthamoeba castellanii mamavirus]	1136	100%	0.0	100.00%
500	417	570142	571395	-	hypothetical protein [Acanthamoeba castellanii mamavirus]	842	100%	0.0	100.00%
501	236	571441	572151	-	hypothetical protein [Acanthamoeba castellanii mamavirus]	476	100%	3,00E-169	100.00%
502	1700	572265	577367	-	hypothetical protein [Acanthamoeba castellanii mamavirus]	3431	100%	0.0	100.00%
503	401	577551	578756	+	hypothetical protein [Acanthamoeba castellanii mamavirus]	804	100%	0.0	100.00%
504	357	578840	579913	-	DNA directed RNA polymerase subunit L [Acanthamoeba castellanii mamavirus]	725	100%	0.0	100.00%
505	403	580042	581253	+	Polynucleotide phosphatase/kinase [Acanthamoeba castellanii mamavirus]	823	100%	0.0	100.00%
506	212	581256	581894	-	hypothetical protein HIRU_S485 [Hirudovirus strain Sangsue]	431	100%	1,00E-152	100.00%
507	321	581959	582924	-	hypothetical protein MIMI_gp0501 [Acanthamoeba polyphaga mimivirus]	649	100%	0.0	100.00%
508	395	583069	584256	-	hypothetical protein MIMI_gp0500 [Acanthamoeba polyphaga mimivirus]	791	100%	0.0	100.00%
509	180	584371	584913	-	hypothetical protein MIMI_gp0499 [Acanthamoeba polyphaga mimivirus]	366	100%	9,00E-128	100.00%
510	106	584979	585299	-	protein translation factor SUI1-like protein [Acanthamoeba polyphaga mimivirus]	215	100%	3,00E-70	100.00%
511	293	585339	586220	-	hypothetical protein MIMI_gp0497 [Acanthamoeba polyphaga mimivirus]	582	100%	0.0	100.00%
512	200	586407	587009	+	hypothetical protein MIMI_gp0496 [Acanthamoeba polyphaga mimivirus]	404	100%	6,00E-142	100.00%
513	209	587092	587721	+	hypothetical protein MIMI_gp0495 [Acanthamoeba polyphaga mimivirus]	432	100%	4,00E-153	100.00%
514	158	587849	588325	+	probable ubiquitin-conjugating enzyme E2 [Acanthamoeba polyphaga mimivirus]	317	100%	5,00E-109	100.00%
515	194	588408	588992	-	hypothetical protein MIMI_gp0493 [Acanthamoeba polyphaga mimivirus]	405	100%	5,00E-143	100.00%
516	524	589157	590731	-	putative translation initiation factor 4a [Acanthamoeba polyphaga mimivirus]	1085	100%	0.0	100.00%
517	189	590850	591419	-	hypothetical protein MIMI_gp0491 [Acanthamoeba polyphaga mimivirus]	400	100%	8,00E-141	100.00%
518	175	591535	592062	+	hypothetical protein MIMI_gp0490 [Acanthamoeba polyphaga mimivirus]	348	100%	5,00E-121	100.00%
519	230	592089	592781	-	hypothetical protein MIMI_gp0489 [Acanthamoeba polyphaga mimivirus]	467	100%	6,00E-166	100.00%
520	1257	592906	596679	+	hypothetical protein [Acanthamoeba castellanii mamavirus]	2579	100%	0.0	100.00%
521	367	596774	597877	-	TATA-box-binding protein-like protein [Acanthamoeba polyphaga mimivirus]	734	100%	0.0	100.00%
522	502	598015	599523	+	hypothetical protein [Acanthamoeba polyphaga mimivirus]	966	100%	0.0	100.00%
523	410	599644	600876	+	Holliday junction resolvase [Acanthamoeba castellanii mamavirus]	827	100%	0.0	100.00%
524	355	600937	602004	-	putative viral transcription factor [Acanthamoeba polyphaga mimivirus]	723	100%	0.0	100.00%
525	661	602089	604074	-	hypothetical protein MIMI_gp0482 [Acanthamoeba polyphaga mimivirus]	1344	100%	0.0	99.85%
526	57	604084	604257	+	orphan [Mimivirus reunion]	112	100%	5,00E-31	98.25%
527	222	604306	604974	+	hypothetical protein MIMI_gp0481 [Acanthamoeba polyphaga mimivirus]	442	100%	2,00E-156	100.00%
528	123	604975	605346	-	hypothetical protein MIMI_gp0480 [Acanthamoeba polyphaga mimivirus]	255	100%	1,00E-85	100.00%
529	332	605496	606494	+	putative patatin-like phospholipase [Acanthamoeba polyphaga mimivirus]	669	100%	0.0	100.00%
530	368	606549	607655	-	Chaperone protein DnaJ [Acanthamoeba castellanii mamavirus]	751	100%	0.0	100.00%
531	513	607757	609298	+	putative ADP-ribosyl glycohydrolase [Acanthamoeba polyphaga mimivirus]	1059	100%	0.0	100.00%
532	164	609392	609886	-	thioredoxin domain-containing protein [Acanthamoeba polyphaga mimivirus]	334	100%	1,00E-115	100.00%
533	1259	610059	613838	+	hypothetical protein [Acanthamoeba castellanii mamavirus]	2583	100%	0.0	100.00%
534	512	613935	615473	-	probable capsid protein 4 [Acanthamoeba polyphaga mimivirus]	1063	100%	0.0	100.00%
535	2156	615566	622036	-	Capsid protein [Acanthamoeba castellanii mamavirus]	4319	100%	0.0	100.00%
536	591	622108	623883	-	Capsid protein [Acanthamoeba castellanii mamavirus]	1206	100%	0.0	100.00%
537	200	624005	624607	+	membrane protein [Acanthamoeba castellanii mamavirus]	400	100%	1,00E-140	100.00%
538	134	624660	625064	-	hypothetical protein [Acanthamoeba castellanii mamavirus]	269	100%	7,00E-91	100.00%
539	284	625133	625987	+	VV A32 virion packaging ATPase [Acanthamoeba polyphaga mimivirus]	590	100%	0.0	100.00%
540	276	625977	626807	-	Serine/Threonine protein kinase [Acanthamoeba castellanii mamavirus]	543	100%	0.0	100.00%
541	88	626887	627153	-	hypothetical protein MIMI_gp0466 [Acanthamoeba polyphaga mimivirus]	176	100%	1,00E-55	100.00%
542	407	627176	628399	-	hypothetical protein MIMI_gp0465 [Acanthamoeba polyphaga mimivirus]	837	100%	0.0	100.00%
543	159	628540	629019	+	hypothetical protein MIMI_gp0464 [Acanthamoeba polyphaga mimivirus]	321	100%	1,00E-110	100.00%
544	76	629109	629339	-	hypothetical protein [Acanthamoeba polyphaga mimivirus]	155	100%	2,00E-47	100.00%
545	57	629317	629490	-	hypothetical protein [Acanthamoeba polyphaga mimivirus]	114	100%	9,00E-32	100.00%
546	192	629624	630202	+	putative HD phosphohydrolase [Acanthamoeba castellanii mamavirus]	393	100%	2,00E-137	100.00%
547	399	630189	631388	-	3'-5' exonuclease [Acanthamoeba castellanii mamavirus]	800	100%	0.0	100.00%
548	256	631468	632238	-	poxvirus late transcription factor VLTF3-like protein [Acanthamoeba castellanii mamavirus]	503	100%	2,00E-179	100.00%
549	431	632298	633593	-	putative transcription factor [Acanthamoeba polyphaga mimivirus]	883	100%	0.0	100.00%
550	194	633702	634286	-	hypothetical protein [Acanthamoeba castellanii mamavirus]	397	100%	1,00E-139	100.00%
551	120	634349	634711	-	hypothetical protein [Acanthamoeba castellanii mamavirus]	241	100%	3,00E-80	100.00%
552	581	635646	637391	+	capsid protein D13L [Hirudovirus strain Sangsue]	1215	100%	0.0	100.00%
553	342	637509	638537	-	hypothetical protein MIMI_gp0454 [Acanthamoeba polyphaga mimivirus]	690	100%	0.0	100.00%
554	530	638624	640216	-	Zn finger-containing protein [Acanthamoeba castellanii mamavirus]	1077	100%	0.0	100.00%
555	232	640374	641072	-	hypothetical protein [Acanthamoeba castellanii mamavirus]	471	100%	1,00E-167	100.00%
556	209	641257	641886	+	hypothetical protein MIMI_gp0451 [Acanthamoeba polyphaga mimivirus]	426	100%	2,00E-150	100.00%
557	244	641883	642617	-	hypothetical protein [Acanthamoeba castellanii mamavirus]	490	100%	1,00E-174	100.00%
558	342	642670	643698	-	uncharacterized short-chain type dehydrogenase/reductase [Acanthamoeba polyphaga mimivirus]	700	100%	0.0	100.00%
559	137	643940	644353	-	nucleoside diphosphate kinase [Acanthamoeba polyphaga mimivirus]	285	100%	4,00E-97	100.00%
560	491	644475	645950	+	hypothetical protein MIMI_gp0447 [Acanthamoeba polyphaga mimivirus]	1011	100%	0.0	100.00%
561	105	646023	646340	+	hypothetical protein MIMI_gp0446 [Acanthamoeba polyphaga mimivirus]	206	100%	8,00E-67	100.00%

562	415	646387	647634	+	FNIP repeat-containing protein [Acanthamoeba castellanii mamavirus]	830	100%	0.0	100.00%
563	73	647699	647920	+	putative F-box and FNIP repeat-containing protein [Hirudovirus strain Sangsue]	140	100%	6,00E-42	93.15%
564	355	647930	648997	-	hypothetical protein [Acanthamoeba castellanii mamavirus]	719	100%	0.0	100.00%
565	284	649097	649951	+	hypothetical protein [Acanthamoeba castellanii mamavirus]	575	100%	0.0	100.00%
566	533	650028	651629	-	replication factor C large subunit [Acanthamoeba castellanii mamavirus]	1071	100%	0.0	100.00%
567	661	651899	653884	+	putative core protein [Acanthamoeba polyphaga mimivirus]	1360	100%	0.0	100.00%
568	177	653972	654505	-	hypothetical protein [Acanthamoeba castellanii mamavirus]	350	100%	2,00E-121	100.00%
569	223	654584	655255	-	putative papain-like cysteine peptidase [Acanthamoeba castellanii mamavirus]	451	100%	4,00E-160	100.00%
570	356	655373	656443	-	putative RNA methyltransferase [Acanthamoeba polyphaga mimivirus]	725	100%	0.0	100.00%
571	191	656512	657087	-	Alkylated DNA repair protein [Acanthamoeba castellanii mamavirus]	401	100%	3,00E-141	100.00%
572	389	657153	658322	-	FtsJ-like methyltransferase family protein [Acanthamoeba castellanii mamavirus]	790	100%	0.0	100.00%
573	263	658396	659187	+	Esterase lipase superfamily protein [Acanthamoeba castellanii mamavirus]	531	100%	0.0	100.00%
574	754	659275	661539	-	hypothetical protein MIMI_gp0433 [Acanthamoeba polyphaga mimivirus]	1551	100%	0.0	100.00%
575	798	661536	663932	-	Low complexity hypothetical protein [Acanthamoeba castellanii mamavirus]	1602	100%	0.0	100.00%
576	226	664048	664728	-	hypothetical protein MIMI_gp0431 [Acanthamoeba polyphaga mimivirus]	449	100%	4,00E-159	100.00%
577	466	664769	666169	-	putative serine/threonine-protein kinase [Acanthamoeba polyphaga mimivirus]	958	100%	0.0	100.00%
578	352	666397	667455	+	hypothetical protein [Acanthamoeba castellanii mamavirus]	686	100%	0.0	100.00%
579	362	667535	668623	-	hypothetical protein MIMI_R398 [Acanthamoeba polyphaga mimivirus]	748	100%	0.0	100.00%
580	589	668827	670596	+	hypothetical protein [Acanthamoeba castellanii mamavirus]	1216	100%	0.0	100.00%
581	519	670739	672298	+	DNA or RNA helicase of superfamily II [Acanthamoeba castellanii mamavirus]	1049	100%	0.0	100.00%
582	319	672370	673329	-	replication factor C small subunit [Acanthamoeba castellanii mamavirus]	640	100%	0.0	100.00%
583	448	673472	674818	+	putative HD domain-containing protein [Acanthamoeba polyphaga mimivirus]	922	100%	0.0	100.00%
584	634	674935	676839	+	HSP70 [Acanthamoeba castellanii mamavirus]	1308	100%	0.0	100.00%
585	617	676890	678743	-	hypothetical protein [Acanthamoeba castellanii mamavirus]	1216	100%	0.0	100.00%
586	763	678760	681051	-	hypothetical protein [Acanthamoeba castellanii mamavirus]	1504	100%	0.0	100.00%
587	1083	681092	684343	+	hypothetical protein [Acanthamoeba castellanii mamavirus]	2191	100%	0.0	100.00%
588	268	684469	685275	+	Endonuclease/exonuclease/phosphatase family protein [Acanthamoeba castellanii mamavirus]	541	100%	0.0	100.00%
589	265	685342	686139	-	hypothetical protein [Acanthamoeba castellanii mamavirus]	519	100%	0.0	100.00%
590	473	686389	687810	+	putative endonuclease of the XPG family [Acanthamoeba castellanii mamavirus]	952	100%	0.0	100.00%
591	141	687880	688305	+	hypothetical protein MIMI_gp0416 [Acanthamoeba polyphaga mimivirus]	283	100%	6,00E-96	100.00%
592	132	688370	688768	+	hypothetical protein MIMI_gp0415 [Acanthamoeba polyphaga mimivirus]	265	100%	3,00E-89	100.00%
593	348	688785	689831	-	divergent methyltransferase [Acanthamoeba castellanii mamavirus]	694	100%	0.0	100.00%
594	1170	689917	693429	-	probable mRNA-capping enzyme [Acanthamoeba polyphaga mimivirus]	2353	100%	0.0	100.00%
595	283	693636	694487	+	hypothetical protein [Acanthamoeba castellanii mamavirus]	570	100%	0.0	100.00%
596	421	694484	695749	-	hypothetical protein [Acanthamoeba castellanii mamavirus]	835	100%	0.0	100.00%
597	304	695817	696731	-	hypothetical protein MIMI_gp0410 [Acanthamoeba polyphaga mimivirus]	619	100%	0.0	100.00%
598	195	696829	697416	-	hypothetical protein MIMI_gp0409 [Acanthamoeba polyphaga mimivirus]	376	100%	4,00E-131	100.00%
599	1144	697634	701068	+	putative NTPase [Acanthamoeba polyphaga mimivirus]	2332	100%	0.0	100.00%
600	199	701129	701728	+	putative DNA-directed RNA polymerase subunit [Acanthamoeba polyphaga mimivirus]	394	100%	6,00E-138	100.00%
601	360	701874	702956	+	putative diphosphoinositol polyphosphate phosphohydrolase [Acanthamoeba polyphaga mimivirus]	731	100%	0.0	100.00%
602	225	702993	703670	+	putative N-acetylglucosaminyl phosphatidylinositol deacetylase [Acanthamoeba polyphaga mimivirus]	458	100%	1,00E-162	100.00%
603	251	703688	704443	+	putative glycosyltransferase [Acanthamoeba polyphaga mimivirus]	513	100%	0.0	99.60%
604	250	704411	705163	-	hypothetical protein MIMI_gp0404 [Acanthamoeba polyphaga mimivirus]	508	100%	0.0	100.00%
605	761	705249	707534	+	ankyrin repeat-containing protein [Acanthamoeba castellanii mamavirus]	1524	100%	0.0	100.00%
606	144	707603	708037	-	hypothetical protein MIMI_gp0401 [Acanthamoeba polyphaga mimivirus]	288	100%	5,00E-98	100.00%
607	445	708134	709471	-	hypothetical protein MIMI_gp0400 [Acanthamoeba polyphaga mimivirus]	893	100%	0.0	100.00%
608	143	709551	709982	-	probable FAD-linked sulfhydryl oxidase [Acanthamoeba polyphaga mimivirus]	293	100%	4,00E-100	100.00%
609	483	710061	711512	-	hypothetical protein MIMI_gp0398 [Acanthamoeba polyphaga mimivirus]	969	100%	0.0	100.00%
610	1766	711590	716890	-	putative ATP-dependent RNA helicase [Acanthamoeba polyphaga mimivirus]	3584	100%	0.0	100.00%
611	393	716981	718162	-	hypothetical protein lvs_R301 [Acanthamoeba polyphaga lentillevirus]	774	100%	0.0	100.00%
612	532	718315	719913	+	DEAD/SNF2 helicase [Acanthamoeba castellanii mamavirus]	1099	100%	0.0	100.00%
613	256	719975	720745	-	putative glycosyltransferase [Acanthamoeba castellanii mamavirus]	528	100%	0.0	100.00%
614	346	720822	721862	-	Thioredoxin [Acanthamoeba castellanii mamavirus]	716	100%	0.0	100.00%
615	262	721989	722777	+	hypothetical protein MIMI_gp0391 [Acanthamoeba polyphaga mimivirus]	536	100%	0.0	100.00%
616	177	722795	723328	+	hypothetical protein [Acanthamoeba castellanii mamavirus]	351	100%	8,00E-122	100.00%
617	1107	723410	726733	+	putative DNA mismatch repair protein MutS-like protein [Acanthamoeba polyphaga mimivirus]	2256	100%	0.0	100.00%
618	272	726773	727591	-	hypothetical protein [Acanthamoeba polyphaga mimivirus]	549	100%	0.0	100.00%
619	73	727679	727900	-	putative DNA-directed RNA polymerase II subunit N [Acanthamoeba polyphaga mimivirus]	151	100%	3,00E-46	100.00%
620	496	728045	729535	+	Low complexity hypothetical protein [Acanthamoeba castellanii mamavirus]	993	100%	0.0	100.00%
621	617	729635	731488	+	hypothetical protein HIRU_S602 [Hirudovirus strain Sangsue]	1188	100%	0.0	98.71%
622	302	731472	732380	-	putative thiol protease [Acanthamoeba castellanii mamavirus]	627	100%	0.0	100.00%
623	549	732487	734136	-	YqaJ viral recombinase family [Acanthamoeba castellanii mamavirus]	1120	100%	0.0	100.00%
624	178	734255	734791	-	hypothetical protein MIMI_gp0382 [Acanthamoeba polyphaga mimivirus]	354	100%	4,00E-123	100.00%
625	292	734920	735798	+	hypothetical protein MIMI_gp0381 [Acanthamoeba polyphaga mimivirus]	593	100%	0.0	100.00%
626	382	735839	736987	-	hypothetical protein [Acanthamoeba castellanii mamavirus]	784	100%	0.0	100.00%
627	817	737069	739522	-	SNF2 family helicase [Acanthamoeba castellanii mamavirus]	1686	100%	0.0	100.00%
628	968	739639	742545	-	hypothetical protein lvs_R284 [Acanthamoeba polyphaga lentillevirus]	1968	100%	0.0	100.00%
629	705	742597	744714	-	hypothetical protein MIMI_gp0377 [Acanthamoeba polyphaga mimivirus]	1408	100%	0.0	100.00%
630	485	744815	746272	-	hypothetical protein MIMI_gp0376 [Acanthamoeba polyphaga mimivirus]	1002	100%	0.0	100.00%
631	195	746363	746950	-	hypothetical protein MIMI_gp0375 [Acanthamoeba polyphaga mimivirus]	386	100%	4,00E-135	100.00%
632	973	747175	750096	-	putative regulator of chromosome condensation [Acanthamoeba polyphaga mimivirus]	1990	100%	0.0	100.00%
633	83	750503	750754	-	hypothetical protein MIMI_gp0373 [Acanthamoeba polyphaga mimivirus]	165	100%	2,00E-51	100.00%
634	526	750869	752449	+	WD-repeat family protein [Acanthamoeba castellanii mamavirus]	1060	100%	0.0	100.00%
635	538	752493	754109	-	probable ribonuclease 3 [Acanthamoeba polyphaga mimivirus]	1090	100%	0.0	100.00%
636	255	754206	754973	-	hypothetical protein lvs_R277 [Acanthamoeba polyphaga lentillevirus]	519	100%	0.0	100.00%
637	584	755054	756808	-	poxvirus poly(A) polymerase catalytic subunit-like protein [Acanthamoeba castellanii mamavirus]	1196	100%	0.0	100.00%
638	324	756982	757956	+	hypothetical protein [Acanthamoeba castellanii mamavirus]	657	100%	0.0	100.00%
639	173	757962	758483	-	transcription factor S-II-related protein [Acanthamoeba polyphaga mimivirus]	353	100%	6,00E-123	100.00%
640	895	758581	761268	-	hypothetical protein [Acanthamoeba castellanii mamavirus]	1818	100%	0.0	100.00%
641	607	761541	763364	-	hypothetical protein lvs_R273 [Acanthamoeba polyphaga lentillevirus]	1205	100%	0.0	100.00%
642	341	763458	764483	-	hypothetical protein [Acanthamoeba castellanii mamavirus]	639	100%	0.0	100.00%
643	187	764647	765210	+	hypothetical protein MIMI_gp0363 [Acanthamoeba polyphaga mimivirus]	363	100%	2,00E-126	100.00%
644	203	765289	765900	+	hypothetical protein [Acanthamoeba castellanii mamavirus]	397	100%	6,00E-139	100.00%
645	208	765989	766615	+	hypothetical protein [Acanthamoeba castellanii mamavirus]	411	100%	1,00E-144	100.00%
646	374	766652	767776	-	hypothetical protein [Acanthamoeba castellanii mamavirus]	770	100%	0.0	100.00%
647	265	767963	768760	+	hypothetical protein [Acanthamoeba castellanii mamavirus]	531	100%	0.0	100.00%
648	184	768856	769410	-	hypothetical protein [Acanthamoeba castellanii mamavirus]	366	100%	1,00E-127	100.00%
649	85	769421	769678	-	hypothetical protein MIMI_gp0357 [Acanthamoeba polyphaga mimivirus]	175	100%	4,00E-55	100.00%
650	361	769730	770815	-	hypothetical protein [Acanthamoeba castellanii mamavirus]	707	100%	0.0	100.00%
651	2064	771062	777256	-	poxvirus early transcription factor-like protein [Acanthamoeba castellanii mamavirus]	4184	100%	0.0	100.00%
652	197	777374	777967	-	putative metallopeptidase WLM [Acanthamoeba castellanii mamavirus]	395	100%	9,00E-139	100.00%
653	348	778116	779162	+	hypothetical protein [Acanthamoeba castellanii mamavirus]	712	100%	0.0	100.00%
654	283	779200	780051	+	Myristylated IMV envelope protein [Acanthamoeba castellanii mamavirus]	558	100%	0.0	100.00%
655	56	780100	780270	+	orphan [Mimivirus reunion]	105	100%	2,00E-28	94.64%

656	1740	780364	785586	-	DNA polymerase [Acanthamoeba polyphaga mimivirus]	3563	100%	0.0	100.00%	
657	184	785692	786246	-	hypothetical protein [Acanthamoeba castellanii mamavirus]	368	100%	3,00E-128	100.00%	
658	253	786375	787136	+	putative methyltransferase [Acanthamoeba polyphaga mimivirus]	518	100%	0.0	100.00%	
659	468	787207	788613	-	putative ubiquitin-specific protease [Acanthamoeba castellanii mamavirus]	979	100%	0.0	100.00%	
660	354	788751	789815	+	probable DNA polymerase family X [Acanthamoeba polyphaga mimivirus]	717	100%	0.0	100.00%	
661	230	789881	790573	-	hypothetical protein [Acanthamoeba castellanii mamavirus]	467	100%	4,00E-166	100.00%	
662	148	790786	791232	+	probable glucosamine 6-phosphate N-acetyltransferase [Acanthamoeba polyphaga mimivirus]	301	100%	3,00E-103	100.00%	
663	287	791261	792124	+	Formamidopyrimidine-DNA glycosylase [Acanthamoeba castellanii mamavirus]	583	100%	0.0	100.00%	
664	138	792209	792625	-	hypothetical protein [Acanthamoeba castellanii mamavirus]	286	100%	1,00E-97	100.00%	
665	881	792755	795400	-	ribonucleoside-diphosphate reductase large subunit [Acanthamoeba polyphaga mimivirus]	1825	100%	0.0	100.00%	
666	417	795555	796808	+	ribonucleotide reductase small subunit [Acanthamoeba castellanii mamavirus]	850	100%	0.0	100.00%	
667	659	796898	798877	-	RING domain-containing protein [Acanthamoeba castellanii mamavirus]	1296	100%	0.0	100.00%	
668	164	799011	799505	+	hypothetical protein MIMI_gp0339 [Acanthamoeba polyphaga mimivirus]	330	100%	5,00E-114	100.00%	
669	95	799568	799855	+	hypothetical protein MIMI_gp0338 [Acanthamoeba polyphaga mimivirus]	192	100%	1,00E-61	100.00%	
670	71	799884	800099	+	hypothetical protein MIMI_gp0337 [Acanthamoeba polyphaga mimivirus]	149	100%	4,00E-45	100.00%	
671	325	800243	801220	+	hypothetical protein [Acanthamoeba polyphaga mimivirus]	660	100%	0.0	100.00%	
672	322	801290	802258	+	putative mRNA capping enzyme [Acanthamoeba polyphaga mimivirus]	649	100%	0.0	100.00%	
673	292	802374	803252	-	putative protein phosphatase 2C [Acanthamoeba polyphaga mimivirus]	607	100%	0.0	100.00%	
674	169	803412	803921	-	hypothetical protein [Acanthamoeba castellanii mamavirus]	339	100%	2,00E-117	100.00%	
675	273	803996	804817	-	hypothetical protein [Acanthamoeba castellanii mamavirus]	545	100%	0.0	100.00%	
676	698	804888	806798	-	NAD-dependent DNA ligase [Acanthamoeba castellanii mamavirus]	1277	100%	0.0	100.00%	
677	114	806870	807214	-	hypothetical protein [Acanthamoeba polyphaga mimivirus]	226	100%	2,00E-74	100.00%	
678	397	807385	808578	-	putative serine/threonine-protein kinase [Acanthamoeba polyphaga mimivirus]	797	100%	0.0	100.00%	
679	166	808612	809112	+	uncharacterized endo/excinuclease amino terminal domain protein [Acanthamoeba polyphaga mimivirus]	338	100%	3,00E-117	100.00%	
680	70	809207	809419	+	hypothetical protein MIMI_gp0327 [Acanthamoeba polyphaga mimivirus]	136	100%	2,00E-40	100.00%	
681	306	809494	810414	-	probable ribonuclease H protein [Acanthamoeba polyphaga mimivirus]	623	100%	0.0	100.00%	
682	284	810475	811329	-	hypothetical protein [Acanthamoeba castellanii mamavirus]	574	100%	0.0	100.00%	
683	295	811396	812283	-	apurinic endonuclease IV [Acanthamoeba castellanii mamavirus]	612	100%	0.0	100.00%	
684	189	812282	812851	+	hypothetical protein [Acanthamoeba polyphaga mimivirus]	370	100%	4,00E-129	99.47%	189
685	2959	812901	821780	+	hypothetical protein [Acanthamoeba castellanii mamavirus]	5943	100%	0.0	100.00%	
686	452	821771	823129	+	peptidase C19 subfamily protein [Acanthamoeba castellanii mamavirus]	908	100%	0.0	100.00%	
687	191	823184	823759	-	hypothetical protein MIMI_gp0320 [Acanthamoeba polyphaga mimivirus]	377	100%	7,00E-132	100.00%	
688	581	823949	825694	+	putative low complexity protein [Acanthamoeba castellanii mamavirus]	1134	100%	0.0	99.66%	
689	548	825822	827468	-	DEAD/SNF2-like helicase [Acanthamoeba castellanii mamavirus]	1128	100%	0.0	100.00%	
690	559	827594	829273	+	ankyrin containing protein [Acanthamoeba polyphaga lentillevirus]	1135	100%	0.0	100.00%	
691	210	829431	830063	+	Ricin-type lectin protein [Acanthamoeba castellanii mamavirus]	425	100%	5,00E-150	100.00%	
692	242	830135	830863	-	hypothetical protein [Acanthamoeba castellanii mamavirus]	487	100%	2,00E-173	100.00%	
693	250	830962	831714	-	hypothetical protein [Acanthamoeba castellanii mamavirus]	503	100%	1,00E-179	100.00%	
694	205	831937	832554	+	hypothetical protein lvs_L234 [Acanthamoeba polyphaga lentillevirus]	434	100%	1,00E-153	100.00%	
695	356	832691	833761	+	hypothetical protein [Acanthamoeba castellanii mamavirus]	729	100%	0.0	100.00%	
696	553	833848	835509	+	hypothetical protein [Acanthamoeba castellanii mamavirus]	1145	100%	0.0	100.00%	
697	152	835621	836079	+	hypothetical protein [Acanthamoeba castellanii mamavirus]	313	100%	2,00E-107	100.00%	
698	292	836167	837045	+	hypothetical protein [Acanthamoeba castellanii mamavirus]	587	100%	0.0	100.00%	
699	197	837138	837731	+	putative acetyltransferase [Acanthamoeba castellanii mamavirus]	402	100%	4,00E-141	100.00%	
700	470	838125	839537	+	ankyrin repeat-containing protein [Acanthamoeba castellanii mamavirus]	960	100%	0.0	100.00%	
701	126	839634	840014	+	hypothetical protein [Acanthamoeba castellanii mamavirus]	261	100%	8,00E-88	100.00%	
702	138	840027	840443	-	hypothetical protein [Acanthamoeba castellanii mamavirus]	280	100%	3,00E-95	100.00%	
703	237	840530	841243	+	putative oxoglutarate/malate carrier protein [Acanthamoeba castellanii mamavirus]	482	100%	6,00E-172	100.00%	
704	79	841700	841939	+	hypothetical protein [Acanthamoeba castellanii mamavirus]	161	100%	5,00E-50	100.00%	
705	435	842047	843354	+	divergent BTB domain-protein [Acanthamoeba castellanii mamavirus]	877	100%	0.0	100.00%	
706	64	843381	843575	-	hypothetical protein [Acanthamoeba castellanii mamavirus]	133	100%	4,00E-39	100.00%	
707	212	843808	844446	+	hypothetical protein MIMI_gp0299 [Acanthamoeba polyphaga mimivirus]	446	100%	4,00E-158	100.00%	
708	446	844662	846002	+	hypothetical protein [Acanthamoeba castellanii mamavirus]	900	100%	0.0	100.00%	
709	771	846095	848410	+	hypothetical protein [Acanthamoeba castellanii mamavirus]	1513	100%	0.0	100.00%	
710	86	848441	848701	+	hypothetical protein [Acanthamoeba castellanii mamavirus]	172	100%	7,00E-54	100.00%	
711	725	848823	851000	+	ankyrin repeat-containing protein [Acanthamoeba castellanii mamavirus]	172	100%	7,00E-54	100.00%	
712	112	851178	851516	+	hypothetical protein lvs_L221 [Acanthamoeba polyphaga lentillevirus]	232	100%	7,00E-77	100.00%	
713	264	851616	852410	+	hypothetical protein [Acanthamoeba castellanii mamavirus]	525	100%	0.0	100.00%	
714	546	852543	854183	+	serine-threonine kinase [Acanthamoeba castellanii mamavirus]	1107	100%	0.0	100.00%	
715	433	854242	855543	-	ankyrin repeat-containing protein [Acanthamoeba castellanii mamavirus]	874	100%	0.0	100.00%	
716	181	855626	856171	-	Dnaj-like protein [Acanthamoeba castellanii mamavirus]	356	100%	8,00E-124	100.00%	
717	63	856375	856566	-	No significant similarity found	-	-	-	-	
718	232	856566	857264	+	hypothetical protein [Samba virus]	458	100%	4,00E-161	100.00%	
719	1396	857370	861560	+	putative WD repeat-containing protein [Hirudovirus strain Sangsue]	2817	100%	0.0	98.87%	
720	583	861609	863360	+	hypothetical protein [Acanthamoeba castellanii mamavirus]	1178	100%	0.0	100.00%	
721	216	863421	864071	+	hypothetical protein MIMI_gp0286 [Acanthamoeba polyphaga mimivirus]	436	100%	2,00E-154	100.00%	
722	181	864080	864625	-	hypothetical protein [Acanthamoeba castellanii mamavirus]	372	100%	6,00E-130	100.00%	
723	398	864719	865915	-	Dnaj-like protein [Acanthamoeba castellanii mamavirus]	828	100%	0.0	100.00%	
724	292	866011	866889	-	putative tRNA(His) guanylyltransferase [Acanthamoeba polyphaga mimivirus]	602	100%	0.0	100.00%	
725	216	867130	867780	+	thymidine kinase [Acanthamoeba polyphaga mimivirus]	435	100%	9,00E-154	100.00%	
726	335	867861	868868	-	hypothetical protein MIMI_gp0281 [Acanthamoeba polyphaga mimivirus]	684	100%	0.0	100.00%	
727	213	869061	869702	+	hypothetical protein MIMI_gp0280 [Acanthamoeba polyphaga mimivirus]	420	100%	4,00E-148	100.00%	
728	312	869770	870708	-	hypothetical protein MIMI_gp0279 [Acanthamoeba polyphaga mimivirus]	634	100%	0.0	100.00%	
729	941	870861	873686	+	heat shock protein 70-like protein [Acanthamoeba polyphaga mimivirus]	1915	100%	0.0	100.00%	
730	58	873766	873942	-	hypothetical protein MIMI_gp0277 [Acanthamoeba polyphaga mimivirus]	117	100%	5,00E-33	100.00%	
731	367	874010	875113	-	hypothetical protein HIRU_S710 [Hirudovirus strain Sangsue]	739	100%	0.0	99.73%	
732	268	875141	875947	-	hypothetical protein MIMI_gp0275 [Acanthamoeba polyphaga mimivirus]	517	100%	0.0	100.00%	
733	1024	876232	879306	+	lon protease-like protein [Acanthamoeba polyphaga mimivirus]	2066	100%	0.0	100.00%	
734	80	879330	879572	+	hypothetical protein MIMI_gp0273 [Acanthamoeba polyphaga mimivirus]	165	100%	2,00E-51	100.00%	
735	461	879701	881086	+	putative transcription initiation factor IIb-like protein [Acanthamoeba polyphaga mimivirus]	939	100%	0.0	100.00%	
736	370	881159	882271	+	putative uracil-DNA glycosylase [Acanthamoeba castellanii mamavirus]	760	100%	0.0	100.00%	
737	406	882371	883591	+	hypothetical protein MIMI_gp0270 [Acanthamoeba polyphaga mimivirus]	813	100%	0.0	100.00%	
738	524	883737	885311	+	DNA directed RNA polymerase subunit 2 [Acanthamoeba castellanii mamavirus]	1085	100%	0.0	100.00%	
739	394	885460	886644	+	HNH endonuclease [Acanthamoeba castellanii mamavirus]	801	100%	0.0	100.00%	
740	640	887053	888975	+	DNA directed RNA polymerase subunit 2 [Acanthamoeba castellanii mamavirus]	1336	100%	0.0	100.00%	
741	330	888975	889967	+	uncharacterized HNH endonuclease [Acanthamoeba polyphaga mimivirus]	666	100%	0.0	100.00%	
742	109	890251	890580	+	DNA-directed RNA polymerase subunit 2 [Hirudovirus strain Sangsue]	228	100%	2,00E-75	100.00%	
743	168	890639	891145	+	hypothetical protein [Acanthamoeba castellanii mamavirus]	346	100%	3,00E-120	100.00%	
744	163	891215	891706	+	hypothetical protein [Acanthamoeba castellanii mamavirus]	331	100%	3,00E-114	100.00%	
745	65	891749	891946	+	orphan [Mimivirus reunion]	48.5	66%	1,00E-05	56.90%	
746	812	891934	894372	-	collagen triple helix repeat containing protein [Acanthamoeba polyphaga lentillevirus]	1412	100%	0.0	100.00%	
747	817	894450	896903	-	Collagen triple helix repeat containing protein [Acanthamoeba castellanii mamavirus]	1469	100%	0.0	100.00%	
748	915	897035	899782	-	Collagen triple helix repeat containing protein [Acanthamoeba castellanii mamavirus]	1631	1631	100%	0.0	99.03%
749	441	899811	901136	-	Collagen triple helix repeat containing protein [Acanthamoeba castellanii mamavirus]	866	100%	0.0	100.00%	

750	289	901304	902173	+	hypothetical protein [Acanthamoeba castellanii mamavirus]	566	100%	0.0	100.00%
751	148	902211	902657	+	hypothetical protein [Acanthamoeba castellanii mamavirus]	303	100%	7,00E-104	100.00%
752	205	902731	903348	+	RNA polymerase subunit 5 [Acanthamoeba castellanii mamavirus]	414	100%	8,00E-146	100.00%
753	288	903456	904322	+	hypothetical protein [Acanthamoeba castellanii mamavirus]	584	100%	0.0	100.00%
754	440	904407	905729	+	putative Zn-dependent peptidase [Acanthamoeba castellanii mamavirus]	893	100%	0.0	100.00%
755	633	905831	907732	+	Serine/Threonine protein kinase [Acanthamoeba castellanii mamavirus]	1266	100%	0.0	100.00%
756	191	907795	908370	+	hypothetical protein [Acanthamoeba castellanii mamavirus]	380	100%	3,00E-133	100.00%
757	895	908540	911227	+	Procollagen-lysine,2-oxoglutarate 5-dioxygenase [Acanthamoeba castellanii mamavirus]	1857	100%	0.0	100.00%
758	290	911358	912230	-	ankyrin repeat-containing protein [Acanthamoeba castellanii mamavirus]	593	100%	0.0	100.00%
759	633	912374	914275	+	hypothetical protein [Acanthamoeba castellanii mamavirus]	1270	100%	0.0	100.00%
760	170	914405	914917	+	hypothetical protein [Acanthamoeba castellanii mamavirus]	344	100%	3,00E-119	100.00%
761	691	915007	917082	+	ankyrin repeat-containing protein [Acanthamoeba castellanii mamavirus]	1390	100%	0.0	100.00%
762	531	917154	918749	-	N-terminal divergent BTB domain-containing protein [Acanthamoeba castellanii mamavirus]	1066	100%	0.0	100.00%
763	540	918859	920481	-	putative BTB/POZ domain-containing protein [Acanthamoeba polyphaga mimivirus]	1072	100%	0.0	100.00%
764	156	920619	921089	+	hypothetical protein [Acanthamoeba castellanii mamavirus]	317	100%	3,00E-109	100.00%
765	308	921158	922084	+	Protein lysine methyltransferase [Acanthamoeba castellanii mamavirus]	626	100%	0.0	100.00%
766	854	922182	924746	+	Topoisomerase I [Acanthamoeba castellanii mamavirus]	1727	100%	0.0	100.00%
767	340	924850	925872	-	hypothetical protein MIMI_gp0242 [Acanthamoeba polyphaga mimivirus]	691	100%	0.0	100.00%
768	663	925921	927912	-	putative ankyrin repeat protein [Acanthamoeba polyphaga mimivirus]	1335	100%	0.0	100.00%
769	281	928008	928853	+	hypothetical protein MIMI_gp0240 [Acanthamoeba polyphaga mimivirus]	577	100%	0.0	100.00%
770	732	928912	931110	-	hypothetical protein [Acanthamoeba castellanii mamavirus]	1481	100%	0.0	100.00%
771	226	931292	931972	+	putative ankyrin repeat protein [Acanthamoeba polyphaga mimivirus]	449	100%	2,00E-159	100.00%
772	215	932021	932668	-	Rab family GTPase [Acanthamoeba castellanii mamavirus]	436	100%	2,00E-154	100.00%
773	174	932841	933365	-	hypothetical protein [Acanthamoeba castellanii mamavirus]	352	100%	2,00E-122	100.00%
774	275	933445	934272	-	hypothetical protein [Acanthamoeba castellanii mamavirus]	551	100%	0.0	99.64%
775	262	934371	935159	+	hypothetical protein lvs_L164 [Acanthamoeba polyphaga lentillevirus]	514	100%	0.0	100.00%
776	383	935134	936285	-	hypothetical protein [Acanthamoeba castellanii mamavirus]	766	100%	0.0	100.00%
777	387	936423	937586	-	hypothetical protein [Acanthamoeba castellanii mamavirus]	781	100%	0.0	100.00%
778	192	937730	938308	+	hypothetical protein [Acanthamoeba castellanii mamavirus]	394	100%	2,00E-138	100.00%
779	960	938441	941323	+	primase/helicase [Acanthamoeba castellanii mamavirus]	1994	100%	0.0	100.00%
780	531	941570	943165	+	Protein kinase family protein [Acanthamoeba castellanii mamavirus]	1088	100%	0.0	100.00%
781	64	943418	943612	-	hypothetical protein MIMI_gp0227 [Acanthamoeba polyphaga mimivirus]	129	100%	2,00E-37	100.00%
782	441	943800	945125	+	hypothetical protein [Acanthamoeba castellanii mamavirus]	874	100%	0.0	100.00%
783	337	945202	946215	-	hypothetical protein [Acanthamoeba castellanii mamavirus]	687	100%	0.0	100.00%
784	151	946371	946826	+	hypothetical protein MIMI_gp0224 [Acanthamoeba polyphaga mimivirus]	308	100%	6,00E-106	100.00%
785	373	946835	947956	+	hypothetical protein HIRU_S765 [Hirudovirus strain Sangsue]	699	100%	0.0	99.73%
786	158	948109	948585	+	hypothetical protein [Acanthamoeba castellanii mamavirus]	310	100%	2,00E-106	100.00%
787	234	948666	949370	+	hypothetical protein [Acanthamoeba castellanii mamavirus]	470	100%	4,00E-167	100.00%
788	115	949342	949689	-	hypothetical protein [Acanthamoeba castellanii mamavirus]	227	100%	6,00E-75	100.00%
789	187	949800	950363	-	putative cytidine deaminase [Acanthamoeba castellanii mamavirus]	377	100%	7,00E-132	100.00%
790	1360	950724	954806	-	collagen triple helix repeat containing protein [Acanthamoeba polyphaga lentillevirus]	2402	100%	0.0	100.00%
791	77	955042	955275	-	collagen triple helix repeat-containing protein [Acanthamoeba polyphaga mimivirus]	169	100%	2,00E-46	100.00%
792	564	955559	957253	-	collagen triple helix repeat-containing protein [Acanthamoeba castellanii mamavirus]	2144	99%	0.0	99.82%
793	436	957475	958785	-	Collagen triple helix repeat containing protein [Acanthamoeba castellanii mamavirus]	1384	85%	0.0	100.00%
794	102	959102	959410	-	probable glutaredoxin [Acanthamoeba polyphaga mimivirus]	211	100%	5,00E-69	100.00%
795	336	959483	960493	-	DNA topoisomerase 1b [Acanthamoeba polyphaga mimivirus]	688	100%	0.0	100.00%
796	601	960651	962456	+	putative glycosyl-transferase [Acanthamoeba castellanii mamavirus]	1247	100%	0.0	100.00%
797	255	962557	963324	+	hypothetical protein [Acanthamoeba castellanii mamavirus]	515	100%	0.0	100.00%
798	54	963383	963547	-	hypothetical protein [Acanthamoeba polyphaga mimivirus]	110	100%	2,00E-30	100.00%
799	78	963566	963802	-	hypothetical protein MIMI_gp0212 [Acanthamoeba polyphaga mimivirus]	155	100%	2,00E-47	100.00%
800	276	964023	964853	+	hypothetical protein [Acanthamoeba castellanii mamavirus]	574	100%	0.0	100.00%
801	359	964924	966003	-	ankyrin repeat-containing protein [Acanthamoeba castellanii mamavirus]	716	100%	0.0	100.00%
802	120	966357	966719	+	hypothetical protein MIMI_gp0209 [Acanthamoeba polyphaga mimivirus]	230	100%	8,00E-76	100.00%
803	509	966772	968301	-	Flavin-containing amine oxidoreductase [Acanthamoeba castellanii mamavirus]	1044	100%	0.0	100.00%
804	246	968351	969091	-	hypothetical protein [Acanthamoeba castellanii mamavirus]	508	100%	0.0	100.00%
805	482	970099	971547	-	putative transposase [Acanthamoeba castellanii mamavirus]	983	100%	0.0	100.00%
806	392	971698	972876	+	hypothetical protein MIMI_gp0205 [Acanthamoeba polyphaga mimivirus]	762	100%	0.0	100.00%
807	382	972936	974084	+	hypothetical protein [Acanthamoeba castellanii mamavirus]	757	100%	0.0	100.00%
808	408	974105	975331	+	hypothetical protein [Acanthamoeba castellanii mamavirus]	810	100%	0.0	100.00%
809	389	975406	976575	+	hypothetical protein [Acanthamoeba castellanii mamavirus]	784	100%	0.0	100.00%
810	379	976640	977779	+	hypothetical protein HIRU_S787 [Hirudovirus strain Sangsue]	752	100%	0.0	100.00%
811	367	977847	978950	+	hypothetical protein [Acanthamoeba castellanii mamavirus]	718	100%	0.0	100.00%
812	379	979020	980159	+	hypothetical protein [Acanthamoeba castellanii mamavirus]	748	100%	0.0	100.00%
813	378	980227	981363	+	hypothetical protein [Acanthamoeba castellanii mamavirus]	751	100%	0.0	100.00%
814	245	981515	982252	+	hypothetical protein [Acanthamoeba castellanii mamavirus]	479	100%	2,00E-170	100.00%
815	148	982315	982761	+	hypothetical protein MIMI_L174a [Acanthamoeba polyphaga mimivirus]	213	100%	7,00E-67	81.17%
816	97	982803	983096	+	hypothetical protein HIRU_S792 [Hirudovirus strain Sangsue]	198	100%	4,00E-64	98.97%
817	205	983209	983826	+	putative ATP-dependent RNA helicase [Acanthamoeba castellanii mamavirus]	413	100%	2,00E-145	100.00%
818	391	983907	985082	+	hypothetical protein [Acanthamoeba castellanii mamavirus]	786	100%	0.0	100.00%
819	151	985191	985646	+	hypothetical protein [Acanthamoeba castellanii mamavirus]	306	100%	5,00E-105	100.00%
820	436	985791	987101	+	FNIP repeat-containing protein [Acanthamoeba castellanii mamavirus]	882	100%	0.0	100.00%
821	140	987175	987597	+	hypothetical protein [Acanthamoeba castellanii mamavirus]	290	100%	6,00E-99	100.00%
822	304	987769	988683	+	hypothetical protein [Acanthamoeba castellanii mamavirus]	604	100%	0.0	100.00%
823	251	988774	989529	+	hypothetical protein [Acanthamoeba castellanii mamavirus]	506	100%	1,00E-180	100.00%
824	271	989653	990468	+	hypothetical protein lvs_L124 [Acanthamoeba polyphaga lentillevirus]	544	100%	0.0	100.00%
825	509	990758	992287	+	Cysteinyl-tRNA synthetase [Acanthamoeba castellanii mamavirus]	1053	100%	0.0	100.00%
826	136	992422	992832	+	hypothetical protein MIMI_gp0181 [Acanthamoeba polyphaga mimivirus]	286	100%	2,00E-97	100.00%
827	521	992900	994465	+	putative FNIP repeat-containing protein [Acanthamoeba polyphaga mimivirus]	1048	100%	0.0	100.00%
828	433	994538	995839	-	hypothetical protein MIMI_gp0179 [Acanthamoeba polyphaga mimivirus]	874	100%	0.0	100.00%
829	433	995900	997201	-	hypothetical protein [Acanthamoeba castellanii mamavirus]	885	100%	0.0	100.00%
830	229	997587	998273	-	putative transposase [Acanthamoeba polyphaga lentillevirus]	452	100%	2,00E-160	100.00%
831	138	998717	999133	-	hypothetical protein [Acanthamoeba castellanii mamavirus]	286	100%	2,00E-97	100.00%
832	370	999243	1000355	-	hypothetical protein [Acanthamoeba castellanii mamavirus]	739	100%	0.0	100.00%
833	504	1000505	1002019	+	hypothetical protein [Acanthamoeba castellanii mamavirus]	998	100%	0.0	100.00%
834	253	1002132	1002893	-	hypothetical protein [Acanthamoeba castellanii mamavirus]	527	100%	0.0	100.00%
835	142	1002905	1003333	-	hypothetical protein [Acanthamoeba castellanii mamavirus]	287	100%	7,00E-98	100.00%
836	467	1003371	1004774	-	WD40 superfamily protein [Acanthamoeba castellanii mamavirus]	945	100%	0.0	100.00%
837	152	1004827	1005285	+	hypothetical protein MIMI_gp0171 [Acanthamoeba polyphaga mimivirus]	307	100%	3,00E-105	100.00%
838	566	1005385	1007085	+	hypothetical protein [Acanthamoeba castellanii mamavirus]	1150	100%	0.0	100.00%
839	206	1007198	1007818	-	N-acetyltransferase [Acanthamoeba castellanii mamavirus]	421	100%	1,00E-148	100.00%
840	266	1008012	1008812	-	hypothetical protein [Acanthamoeba castellanii mamavirus]	553	100%	0.0	100.00%
841	173	1009091	1009612	+	hypothetical protein [Acanthamoeba castellanii mamavirus]	343	100%	4,00E-119	100.00%
842	229	1009679	1010368	+	ankyrin repeat-containing protein [Acanthamoeba castellanii mamavirus]	461	100%	9,00E-164	100.00%
843	279	1010413	1011252	+	hypothetical protein MIMI_gp0165 [Acanthamoeba polyphaga mimivirus]	568	100%	0.0	100.00%

844	346	1011308	1012348	+	hypothetical protein MIMI_gp0164 [Acanthamoeba polyphaga mimivirus]	685	100%	0.0	100.00%
845	274	1012431	1013255	+	hypothetical protein MIMI_gp0163 [Acanthamoeba polyphaga mimivirus]	556	100%	0.0	100.00%
846	233	1013314	1014015	+	hypothetical protein MIMI_gp0162 [Acanthamoeba polyphaga mimivirus]	470	100%	4,00E-167	100.00%
847	287	1014121	1014984	+	putative exoV-like protein [Acanthamoeba polyphaga mimivirus]	594	100%	0.0	100.00%
848	490	1015076	1016548	+	putative WbbJ Acetyltransferase [Acanthamoeba polyphaga mimivirus]	998	100%	0.0	100.00%
849	323	1016706	1017677	-	putative dTDP-D-glucose 4,6-dehydratase [Acanthamoeba polyphaga mimivirus]	669	100%	0.0	100.00%
850	304	1017829	1018743	+	hypothetical protein MIMI_gp0157 [Acanthamoeba polyphaga mimivirus]	626	100%	0.0	100.00%
851	248	1018691	1019437	-	uncharacterized glycosyltransferase [Acanthamoeba polyphaga mimivirus]	517	100%	0.0	100.00%
852	883	1019466	1022117	+	hypothetical protein MIMI_gp0156 [Acanthamoeba polyphaga mimivirus]	1798	100%	0.0	100.00%
853	721	1022220	1024385	+	hypothetical protein [Acanthamoeba castellanii mamavirus]	1485	100%	0.0	100.00%
854	352	1024404	1025462	+	Aminotransferase [Acanthamoeba castellanii mamavirus]	722	100%	0.0	100.00%
855	702	1025546	1027654	-	choline dehydrogenase-like protein [Acanthamoeba castellanii mamavirus]	1448	100%	0.0	100.00%
856	193	1027786	1028367	+	hypothetical protein [Acanthamoeba castellanii mamavirus]	396	100%	2,00E-139	100.00%
857	127	1028438	1028821	-	hypothetical protein MIMI_gp0151 [Acanthamoeba polyphaga mimivirus]	252	100%	2,00E-84	100.00%
858	221	1028928	1029593	-	Methyltransferase family protein [Acanthamoeba castellanii mamavirus]	454	100%	2,00E-161	100.00%
859	178	1029667	1030203	-	hypothetical protein lvs_R89 [Acanthamoeba polyphaga lentillevirus]	358	100%	2,00E-124	100.00%
860	421	1030331	1031596	-	hypothetical protein [Acanthamoeba castellanii mamavirus]	832	100%	0.0	100.00%
861	70	1031702	1031914	+	hypothetical protein MIMI_gp0146 [Acanthamoeba polyphaga mimivirus]	144	100%	4,00E-43	100.00%
862	563	1032054	1033745	+	Choline dehydrogenase [Acanthamoeba castellanii mamavirus]	1159	100%	0.0	100.00%
863	269	1033876	1034685	+	putative F-box protein [Acanthamoeba polyphaga mimivirus]	555	100%	0.0	100.00%
864	255	1034781	1035548	+	putative F-box protein [Acanthamoeba polyphaga mimivirus]	524	100%	0.0	100.00%
865	196	1035545	1036135	-	BRO-M fanily protein [Acanthamoeba castellanii mamavirus]	398	100%	8,00E-140	100.00%
866	346	1036256	1037296	+	Tyrosyl-tRNA synthetase [Acanthamoeba castellanii mamavirus]	720	100%	0.0	100.00%
867	83	1037458	1037709	+	hypothetical protein MIMI_gp0140 [Acanthamoeba polyphaga mimivirus]	177	100%	6,00E-56	98.80%
868	627	1037777	1039660	+	ankyrin repeat-containing protein [Acanthamoeba castellanii mamavirus]	1273	100%	0.0	100.00%
869	324	1039774	1040748	+	ankyrin repeat-containing protein [Acanthamoeba castellanii mamavirus]	651	100%	0.0	100.00%
870	339	1040816	1041835	+	ankyrin repeat-containing protein [Acanthamoeba castellanii mamavirus]	682	100%	0.0	100.00%
871	75	1041807	1042034	-	hypothetical protein HIRU_S850 [Hirudovirus strain Sangsue]	155	100%	1,00E-47	100.00%
872	165	1042074	1042571	-	hypothetical protein [Acanthamoeba castellanii mamavirus]	335	100%	6,00E-116	100.00%
873	356	1042653	1043723	-	hypothetical protein MIMI_gp0135 [Acanthamoeba polyphaga mimivirus]	741	100%	0.0	100.00%
874	97	1043829	1044122	+	hypothetical protein [Acanthamoeba castellanii mamavirus]	201	100%	3,00E-65	100.00%
875	535	1044303	1045910	+	hypothetical protein [Acanthamoeba castellanii mamavirus]	1097	100%	0.0	100.00%
876	1261	1046011	1049796	+	Helicase, SNF2 family [Acanthamoeba castellanii mamavirus]	2581	100%	0.0	99.92%
877	67	1049875	1050078	+	hypothetical protein [Acanthamoeba castellanii mamavirus]	142	100%	1,00E-42	100.00%
878	183	1051229	1051780	+	hypothetical protein [Acanthamoeba castellanii mamavirus]	366	100%	9,00E-128	100.00%
879	458	1051857	1053233	+	ankyrin repeat-containing protein [Acanthamoeba castellanii mamavirus]	932	100%	0.0	100.00%
880	374	1053303	1054427	+	hypothetical protein [Acanthamoeba castellanii mamavirus]	766	100%	0.0	100.00%
881	150	1054717	1055169	+	hypothetical protein [Acanthamoeba castellanii mamavirus]	302	100%	1,00E-103	100.00%
882	150	1055306	1055758	+	hypothetical protein [Acanthamoeba castellanii mamavirus]	299	100%	3,00E-102	100.00%
883	251	1055745	1056500	+	ankyrin repeat-containing protein [Acanthamoeba castellanii mamavirus]	504	100%	6,00E-180	100.00%
884	279	1056586	1057425	+	Proliferating cell nuclear antigen [Acanthamoeba castellanii mamavirus]	549	100%	0.0	100.00%
885	498	1057558	1059054	+	Proliferating cell nuclear antigen [Acanthamoeba castellanii mamavirus]	549	100%	0.0	100.00%
886	420	1059093	1060355	-	BTB domain-containing protein [Acanthamoeba castellanii mamavirus]	1009	100%	0.0	100.00%
887	415	1060521	1061768	+	BTB domain-containing protein [Acanthamoeba castellanii mamavirus]	834	100%	0.0	100.00%
888	344	1061917	1062951	+	hypothetical protein [Acanthamoeba castellanii mamavirus]	707	100%	0.0	100.00%
889	599	1063141	1064940	+	ankyrin repeat-containing protein [Acanthamoeba castellanii mamavirus]	1221	100%	0.0	100.00%
890	272	1065100	1065918	+	ankyrin repeat-containing protein [Acanthamoeba castellanii mamavirus]	555	100%	0.0	100.00%
891	503	1065987	1067498	+	BTB domain-containing protein [Acanthamoeba castellanii mamavirus]	1008	100%	0.0	100.00%
892	279	1067561	1068400	-	hypothetical protein [Acanthamoeba castellanii mamavirus]	572	100%	0.0	100.00%
893	1025	1068497	1071574	-	ankyrin repeat-containing protein [Acanthamoeba castellanii mamavirus]	2080	100%	0.0	100.00%
894	327	1071740	1072723	+	ankyrin repeat-containing protein [Acanthamoeba castellanii mamavirus]	666	100%	0.0	100.00%
895	175	1072810	1073337	+	hypothetical protein [Acanthamoeba castellanii mamavirus]	362	100%	3,00E-126	100.00%
896	808	1073460	1075886	+	ankyrin repeat-containing protein [Acanthamoeba castellanii mamavirus]	1640	100%	0.0	100.00%
897	764	1076015	1078309	+	putative ankyrin repeat protein [Acanthamoeba polyphaga mimivirus]	1551	100%	0.0	100.00%
898	502	1078441	1079949	+	putative BTB/POZ domain-containing protein [Acanthamoeba polyphaga mimivirus]	1007	100%	0.0	100.00%
899	879	1080548	1083187	+	ankyrin repeat-containing protein [Acanthamoeba castellanii mamavirus]	1767	100%	0.0	100.00%
900	171	1083306	1083821	+	hypothetical protein MIMI_gp0105 [Acanthamoeba polyphaga mimivirus]	355	100%	7,00E-124	100.00%
901	576	1083952	1085682	+	putative ankyrin repeat protein [Acanthamoeba polyphaga mimivirus]	1162	100%	0.0	100.00%
902	559	1085800	1087479	+	putative BTB/POZ domain-containing protein [Mimivirus reunion]	1123	100%	0.0	99.64%
903	133	1087802	1088203	-	hypothetical protein MIMI_gp0100 [Acanthamoeba polyphaga mimivirus]	273	100%	3,00E-92	100.00%
904	181	1088477	1089022	+	hypothetical protein MIMI_gp0099 [Acanthamoeba polyphaga mimivirus]	374	100%	8,00E-131	100.00%
905	200	1089141	1089743	+	ankyrin repeat-containing protein [Acanthamoeba castellanii mamavirus]	422	100%	5,00E-149	100.00%
906	99	1090221	1090520	+	hypothetical protein [Acanthamoeba castellanii mamavirus]	198	100%	5,00E-64	100.00%
907	103	1090588	1090899	+	hypothetical protein MIMI_gp0095 [Acanthamoeba polyphaga mimivirus]	208	100%	9,00E-68	100.00%
908	87	1091005	1091268	+	hypothetical protein MIMI_gp0094 [Acanthamoeba polyphaga mimivirus]	174	100%	1,00E-54	100.00%
909	242	1091525	1092253	+	hypothetical protein MIMI_gp0093 [Acanthamoeba polyphaga mimivirus]	493	100%	4,00E-176	100.00%
910	553	1092365	1094026	+	hypothetical protein lvs_L46 [Acanthamoeba polyphaga lentillevirus]	1090	100%	0.0	100.00%
911	378	1094144	1095280	-	ankyrin repeat-containing protein [Acanthamoeba castellanii mamavirus]	751	100%	0.0	100.00%
912	143	1095388	1095819	+	hypothetical protein [Acanthamoeba castellanii mamavirus]	278	100%	5,00E-94	100.00%
913	308	1095940	1096866	+	hypothetical protein [Acanthamoeba castellanii mamavirus]	607	100%	0.0	100.00%
914	238	1097571	1098287	+	hypothetical protein [Acanthamoeba castellanii mamavirus]	489	100%	2,00E-174	100.00%
915	227	1098502	1099185	-	hypothetical protein [Acanthamoeba castellanii mamavirus]	449	100%	4,00E-159	100.00%
916	384	1099345	1100499	+	ankyrin repeat-containing protein [Acanthamoeba castellanii mamavirus]	764	100%	0.0	100.00%
917	894	1100851	1103535	+	Collagen triple helix repeat containing protein [Acanthamoeba castellanii mamavirus]	1563	100%	0.0	100.00%
918	312	1103635	1104573	-	hypothetical protein MIMI_gp0085 [Acanthamoeba polyphaga mimivirus]	640	100%	0.0	100.00%
919	231	1104755	1105450	+	hypothetical protein MIMI_gp0084 [Acanthamoeba polyphaga mimivirus]	468	100%	3,00E-166	100.00%
920	243	1105710	1106441	+	ankyrin repeat-containing protein [Hirudovirus strain Sangsue]	487	100%	1,00E-173	100.00%
921	498	1106595	1108091	+	hypothetical protein [Acanthamoeba castellanii mamavirus]	994	100%	0.0	100.00%
922	587	1108250	1110013	+	Putative ankyrin repeat protein L66 [Acanthamoeba polyphaga mimivirus]	1148	100%	0.0	100.00%
923	355	1110167	1111234	+	uncharacterized virion-associated membrane protein [Acanthamoeba polyphaga mimivirus]	724	100%	0.0	100.00%
924	565	1111466	1113163	+	hypothetical protein [Acanthamoeba castellanii mamavirus]	1147	100%	0.0	100.00%
925	580	1113281	1115023	+	ankyrin repeat-containing protein [Acanthamoeba castellanii mamavirus]	1172	100%	0.0	100.00%
926	119	1115076	1115435	+	hypothetical protein [Acanthamoeba castellanii mamavirus]	234	100%	2,00E-77	100.00%
927	282	1115529	1116377	+	ankyrin-containing protein [Acanthamoeba polyphaga mimivirus]	739	100%	0.0	93.62%
928	62	1116467	1116655	+	hypothetical protein [Acanthamoeba castellanii mamavirus]	115	100%	2,00E-32	100.00%
929	146	1116731	1117171	-	hypothetical protein [Acanthamoeba castellanii mamavirus]	301	100%	3,00E-103	99.32%
930	496	1117346	1118836	-	WD-repeat family protein [Acanthamoeba castellanii mamavirus]	998	100%	0.0	100.00%
931	621	1119273	1121138	+	putative F-box and FNIP repeat-containing protein [Acanthamoeba polyphaga mimivirus]	1244	100%	0.0	100.00%
932	307	1121230	1122153	+	putative ankyrin repeat protein [Acanthamoeba polyphaga mimivirus]	630	100%	0.0	100.00%
933	131	1122262	1122657	-	hypothetical protein [Acanthamoeba castellanii mamavirus]	253	100%	2,00E-84	100.00%
934	182	1122833	1123381	+	hypothetical protein [Acanthamoeba castellanii mamavirus]	367	100%	5,00E-128	100.00%
935	604	1123498	1125312	+	ankyrin repeat-containing protein [Acanthamoeba castellanii mamavirus]	1221	100%	0.0	100.00%
936	513	1125499	1127040	+	hypothetical protein [Acanthamoeba castellanii mamavirus]	1034	100%	0.0	100.00%
937	182	1127119	1127667	+	hypothetical protein [Acanthamoeba castellanii mamavirus]	367	100%	7,00E-128	100.00%

938	100	1127740	1128042	+	hypothetical protein MIMI_gp0065 [Acanthamoeba polyphaga mimivirus]	172	100%	1,00E-53	100.00%
939	127	1128124	1128507	+	hypothetical protein HIRU_S925 [Hirudovirus strain Sangsue]	254	100%	3,00E-85	100.00%
940	159	1128637	1129116	+	hypothetical protein [Acanthamoeba castellanii mamavirus]	327	100%	5,00E-113	100.00%
941	113	1129164	1129505	-	hypothetical protein [Acanthamoeba castellanii mamavirus]	233	100%	2,00E-77	100.00%
942	186	1129664	1130224	+	hypothetical protein [Acanthamoeba castellanii mamavirus]	373	100%	3,00E-130	100.00%
943	120	1130378	1130740	+	hypothetical protein [Acanthamoeba castellanii mamavirus]	245	100%	6,00E-82	100.00%
944	100	1130764	1131066	-	No significant similarity found	-	-	-	-
945	201	1131282	1131887	-	hypothetical protein MIMI_gp0058 [Acanthamoeba polyphaga mimivirus]	405	100%	2,00E-142	100.00%
946	164	1132095	1132589	+	hypothetical protein MIMI_gp0057 [Acanthamoeba polyphaga mimivirus]	343	100%	4,00E-119	100.00%
947	227	1132878	1133561	+	putative ankyrin repeat protein [Acanthamoeba polyphaga mimivirus]	458	100%	1,00E-162	100.00%
948	215	1133623	1134270	-	hypothetical protein MIMI_gp0055 [Acanthamoeba polyphaga mimivirus]	433	100%	4,00E-153	100.00%
949	212	1134405	1135043	+	hypothetical protein MIMI_gp0054 [Acanthamoeba polyphaga mimivirus]	430	100%	7,00E-152	100.00%
950	51	1135083	1135238	+	hypothetical protein MIMI_gp0053 [Acanthamoeba polyphaga mimivirus]	105	100%	2,00E-28	100.00%
951	357	1135393	1136466	+	putative ankyrin repeat protein [Acanthamoeba polyphaga mimivirus]	726	100%	0.0	100.00%
952	478	1136557	1137993	+	hypothetical protein MIMI_gp0051 [Acanthamoeba polyphaga mimivirus]	971	100%	0.0	100.00%
953	58	1138077	1138253	+	hypothetical protein MIMI_gp0050 [Acanthamoeba polyphaga mimivirus]	118	100%	2,00E-33	100.00%
954	91	1138463	1138738	+	hypothetical protein MIMI_gp0049 [Acanthamoeba polyphaga mimivirus]	177	100%	8,00E-56	100.00%
955	101	1138950	1139255	-	hypothetical protein MIMI_gp0048 [Acanthamoeba polyphaga mimivirus]	194	100%	4,00E-62	100.00%
956	150	1139539	1139991	+	hypothetical protein MIMI_gp0047 [Acanthamoeba polyphaga mimivirus]	312	100%	5,00E-107	100.00%
957	113	1140122	1140463	+	hypothetical protein [Acanthamoeba castellanii mamavirus]	222	100%	6,00E-73	99.12%
958	97	1140417	1140710	+	putative ankyrin repeat protein [Acanthamoeba polyphaga mimivirus]	196	100%	3,00E-63	100.00%
959	189	1140938	1141507	+	putative ankyrin repeat protein [Acanthamoeba polyphaga mimivirus]	375	100%	5,00E-131	100.00%
960	90	1141553	1141825	+	hypothetical protein [Acanthamoeba polyphaga mimivirus]	171	100%	1,00E-53	98.89%
961	487	1142008	1143471	+	putative Kila-N domain-containing protein [Acanthamoeba polyphaga mimivirus]	979	100%	0.0	100.00%
962	116	1143612	1143962	+	hypothetical protein [Acanthamoeba castellanii mamavirus]	229	100%	1,00E-75	99.14%
963	187	1144016	1144579	+	ankyrin repeat-containing protein [Acanthamoeba castellanii mamavirus]	376	100%	2,00E-131	100.00%
964	493	1144682	1146163	+	hypothetical protein [Acanthamoeba castellanii mamavirus]	982	100%	0.0	100.00%
965	84	1146191	1146445	+	hypothetical protein [Acanthamoeba castellanii mamavirus]	171	100%	9,00E-54	100.00%
966	409	1146549	1147778	-	hypothetical protein [Acanthamoeba castellanii mamavirus]	810	100%	0.0	100.00%
967	73	1147953	1148174	+	hypothetical protein [Acanthamoeba castellanii mamavirus]	143	100%	8,00E-43	100.00%
968	545	1148340	1149977	+	KilA N-domain-containing protein [Acanthamoeba castellanii mamavirus]	1105	100%	0.0	100.00%
969	108	1150168	1150494	-	hypothetical protein MIMI_gp0032 [Acanthamoeba polyphaga mimivirus]	227	100%	4,00E-75	100.00%
970	94	1150552	1150836	+	hypothetical protein [Acanthamoeba castellanii mamavirus]	189	100%	1,00E-60	100.00%
971	104	1150794	1151108	+	hypothetical protein MIMI_gp0031 [Acanthamoeba polyphaga mimivirus]	207	100%	4,00E-67	100.00%
972	146	1151255	1151695	+	hypothetical protein MIMI_gp0030 [Acanthamoeba polyphaga mimivirus]	295	100%	8,00E-101	100.00%
973	104	1151863	1152177	+	hypothetical protein MIMI_gp0029 [Acanthamoeba polyphaga mimivirus]	216	100%	5,00E-71	100.00%
974	326	1152184	1153164	+	ankyrin repeat-containing protein [Acanthamoeba castellanii mamavirus]	648	100%	0.0	100.00%
975	102	1153381	1153689	-	hypothetical protein [Acanthamoeba castellanii mamavirus]	212	100%	3,00E-69	100.00%
976	63	1153688	1153879	+	No significant similarity found	-	-	-	-
977	236	1154152	1154862	+	ankyrin repeat-containing protein [Acanthamoeba castellanii mamavirus]	473	100%	4,00E-168	100.00%
978	122	1154988	1155356	+	putative ankyrin repeat protein [Acanthamoeba polyphaga mimivirus]	238	100%	6,00E-79	100.00%
979	533	1155490	1157091	+	putative sel1-like repeat-containing protein [Acanthamoeba polyphaga mimivirus]	1085	100%	0.0	100.00%
980	92	1157276	1157554	+	hypothetical protein MIMI_gp0023 [Acanthamoeba polyphaga mimivirus]	188	100%	8,00E-60	100.00%
981	123	1157695	1158066	+	hypothetical protein MIMI_gp0022 [Acanthamoeba polyphaga mimivirus]	245	100%	8,00E-82	100.00%
982	584	1158186	1159940	+	putative sel1-like repeat-containing protein [Acanthamoeba polyphaga mimivirus]	1154	100%	0.0	98.12%
983	359	1160414	1161493	+	hypothetical protein [Acanthamoeba castellanii mamavirus]	717	100%	0.0	100.00%
984	269	1161606	1162415	+	hypothetical protein MIMI_gp0019 [Acanthamoeba polyphaga mimivirus]	541	100%	0.0	100.00%
985	91	1162626	1162901	+	hypothetical protein MIMI_gp0018 [Acanthamoeba polyphaga mimivirus]	193	100%	4,00E-62	100.00%
986	143	1163334	1163765	+	hypothetical protein [Acanthamoeba castellanii mamavirus]	295	100%	7,00E-101	100.00%
987	338	1164199	1165215	+	poly(A) polymerase small subunit [Megavirus chiliensis]	600	99%	0.0	90.56%
988	213	1166940	1167581	+	hypothetical protein [Acanthamoeba castellanii mamavirus]	353	81%	6,00E-122	100.00%
989	181	1170371	1170916	+	hypothetical protein [Cotonvirus japonicus]	194	62%	9,00E-61	85.09%
990	53	1171816	1171977	+	No significant similarity found	-	-	-	-
991	67	1172586	1172789	-	putative membrane protein [Acanthamoeba castellanii mamavirus]	58.5	62%	2,00E-09	69.05%
992	73	1174120	1174341	+	No significant similarity found	-	-	-	-
993	312	1176386	1177324	-	hypothetical protein [Acanthamoeba castellanii mamavirus]	630	100%	0.0	100.00%
994	147	1177517	1177960	+	hypothetical protein [Acanthamoeba castellanii mamavirus]	295	100%	9,00E-101	100.00%
995	408	1178190	1179416	+	hypothetical protein [Mimivirus reunion]	636	100%	0.0	84.31%
996	481	1179711	1181156	+	KilA N-terminal domain, N1R/P28 DNA binding protein [Acanthamoeba polyphaga lentillevirus]	586	58%	0.0	100.00%
997	393	1181804	1182985	+	KilA N-terminal domain, N1R/P28 DNA binding protein [Acanthamoeba polyphaga lentillevirus]	785	100%	0.0	100.00%
998	242	1183101	1183829	+	KilA N family protein [Acanthamoeba castellanii mamavirus]	462	98%	2,00E-163	97.48%
999	669	1184167	1186176	+	hypothetical protein [Acanthamoeba castellanii mamavirus]	1337	100%	0.0	100.00%
1000	110	1186259	1186591	+	putative Bro-N domain-containing protein [Hirudovirus strain Sangsue]	209	92%	8,00E-67	99.02%
1001	93	1186866	1187147	-	hypothetical protein [Acanthamoeba castellanii mamavirus]	190	100%	6,00E-61	100.00%
1002	1052	1187472	1190630	-	Helicase [Acanthamoeba castellanii mamavirus]	2130	100%	0.0	100.00%
1003	155	1190825	1191292	+	hypothetical protein [Acanthamoeba castellanii mamavirus]	308	100%	2,00E-105	100.00%
1004	125	1191676	1192053	-	hypothetical protein [Acanthamoeba castellanii mamavirus]	235	100%	1,00E-77	100.00%
1005	311	1192047	1192982	-	BRO-N domain containing protein [Acanthamoeba castellanii mamavirus]	636	100%	0.0	100.00%
1006	311	1193931	1194866	+	BRO-N domain containing protein [Acanthamoeba castellanii mamavirus]	636	100%	0.0	100.00%
1007	125	1194860	1195237	+	hypothetical protein [Acanthamoeba castellanii mamavirus]	235	100%	1,00E-77	100.00%
1008	155	1195621	1196088	-	hypothetical protein [Acanthamoeba castellanii mamavirus]	308	100%	2,00E-105	100.00%
1009	1052	1196283	1199441	+	Helicase [Acanthamoeba castellanii mamavirus]	2130	100%	0.0	100.00%
1010	93	1199766	1200047	+	hypothetical protein [Acanthamoeba castellanii mamavirus]	190	100%	6,00E-61	100.00%
1011	110	1200322	1200654	-	putative Bro-N domain-containing protein [Hirudovirus strain Sangsue]	209	92%	8,00E-67	99.02%
1012	669	1200737	1202746	-	hypothetical protein [Acanthamoeba castellanii mamavirus]	1337	100%	0.0	100.00%

Note: aa: amino acids nr: non reduntant protein sequences database

Table S2 - Detailed annotation of sputnik argentum genome through BLASTp and HHpred analysis

ORF	Size (aa)	Start	Stop	Strand	Best hit Blastp (nr)	Score	Query cover	e-value	Identity	HHpred best hit	Probability (%)	e-value
1	144	369	803	+	V1 [Sputnik virophage]	277	100%	1,00E-93	100.00%	C4-type zinc ribbon domain [Chlamydia trachomatis 434/Bu]	87.68	0.54
2	114	859	1203	+	V2 [Sputnik virophage]	226	100%	2,00E-74	100.00%	No hit	-	-
3	245	1457	2194	-	V3 [Sputnik virophage]	499	100%	2,00E-178	100.00%	FtsK-HerA superfamily, P-loop ATPase [Sulfolobus turreted icosahedral virus 2]	99.53	2.9e-12
4	139	2285	2704	+	V4 [Sputnik virophage]	291	100%	2,00E-99	100.00%	C2H2-type zinc finger [*Drosophila melanogaster*]	97.86	0.000017
5	119	3113	3472	+	V5 [Sputnik virophage]	228	100%	5,00E-75	100.00%	No hit	-	-
6	310	3523	4455	+	V6 [Sputnik virophage]	595	100%	0.0	100.00%	Collagen alpha-2(I) chain [*Rattus norvegicus*]	97.66	0.0031
7	236	4493	5203	+	V7 [Sputnik virophage]	457	100%	9,00E-162	100.00%	COLLAGEN-LIKE PROTEIN [*Bacillus anthracis*]	98.62	0.000032
8	184	5225	5779	+	V8 [Sputnik virophage]	361	100%	1,00E-125	100.00%	No hit	-	-
9	175	5815	6342	+	V9 [Sputnik virophage]	357	100%	2,00E-124	100.00%	Cysteine proteinases superfamily [Mastadenovirus H2]	99.82	2.2e-19
10	226	6550	7230	-	V10 [Sputnik virophage]	459	100%	4,00E-163	100.00%	Site-specific recombinase [Escherichia coli]	99.88	6.7e-21
11	162	7317	7805	+	hypothetical protein Sputnik2_R11 [Sputnik virophage 2]	317	100%	1,00E-108	100.00%	No hit	-	-
12	152	8509	8967	+	V12 [Sputnik virophage]	292	100%	2,00E-99	100.00%	No hit	-	-
13	779	9409	11748	+	V13 [Sputnik virophage]	1592	100%	0.0	100.00%	Primase; helicase [Nitratiruptor phage NrS-1]	99.95	8.5e-27
14	114	11809	12153	+	V14 [Sputnik virophage]	218	100%	2,00E-71	100.00%	Zinc motif and AAA domain [*Homo sapiens*]	96.56	0.053
15	109	12203	12532	-	V15 [Sputnik virophage]	216	100%	2,00E-70	100.00%	No hit	-	-
16	130	12570	12962	-	V16 [Sputnik virophage]	262	100%	2,00E-88	100.00%	No hit	-	-
17	187	12996	13559	-	putative IS3 family transposase A protein [Sputnik virophage 2]	369	100%	7,00E-129	100.00%	Transcriptional regulator (TetR-type HTH domain) [*Corynebacterium glutamicum*]	97.24	0.0016
18	378	13912	15048	+	minor virion protein [Sputnik virophage 2]	763	100%	0.0	100.00%	Minor virion protein [Sputnik virophage]	100	8.4e-112
19	595	15072	16859	+	V20 [Sputnik virophage]	1214	100%	0.0	100.00%	capsid protein V20 [Sputnik virophage]	100	6.8e-139
20	442	16919	18247	+	V21 [Sputnik virophage]	902	100%	0.0	100.00%	L544; Transferase [Acanthamoeba polyphaga mimivirus]	100	1.4e-40

Note: aa: amino acids nr: non reduntant protein sequences database

Table S3 - Detailed annotation of mimivirus argentum transpoviron through BLASTp and HHpred analysis

ORF	Size (aa)	Start	Stop	Strand	Best hit BLASTp (nr)	Score	Query cover	e-value	Identity
1	201	252	857	+	hypothetical protein tv_R1 [Mimivirus lentille]	404	100%	6,00E-142	100.00%
2	140	854	1276	-	hypothetical protein tv_L2 [Mimivirus lentille]	272	100%	5,00E-92	100.00%
3	136	1309	1719	-	hypothetical protein tv_L3 [Mimivirus lentille]	261	100%	2,00E-87	100.00%
4	112	2112	2450	-	hypothetical protein tv_L4 [Mimivirus lentille]	221	100%	1,00E-72	100.00%
5	999	2924	5923	-	integrase zinc-binding and helicase domain-containing protein [Acanthamoeba castellanii mamavirus]	2033	100%	0.0	100.00%
6	89	6018	6287	+	zinc finger C2H2-type domain-containing protein [Mimivirus lentille]	179	100%	1,00E-56	100.00%

Note: aa: amino acids nr: non reduntant protein sequences database

**HHpred best hit**	**Probability (%)**	**e-value**
No hit	-	-
Transmembrane protein: alpha-helical [*Escherichia coli*]	91.44	1
No hit	-	-
Transposase [*Corynebacterium glutamicum*]	98.52	9.4e-7
Exodeoxyribonuclease v alpha chain; recombination, helicase, nuclease [*E. coli*]	100	1.9e-31
Zinc finger homeobox protein [*Homo sapiens*]	95.21	0.055
